# An Overview of Lipid Biomarkers in Terrestrial Extreme Environments with Relevance for Mars Exploration

**DOI:** 10.1089/ast.2022.0083

**Published:** 2023-04-28

**Authors:** Pablo L. Finkel, Daniel Carrizo, Victor Parro, Laura Sánchez-García

**Affiliations:** ^1^ Centro de Astrobiología (CAB), CSIC-INTA, Madrid, Spain.; ^2^ Department of Physics and Mathematics and Department of Automatics, University of Alcalá, Madrid, Spain.

**Keywords:** Lipid biomarker, Diagenesis, Preservation, Microorganism, Stable carbon, Isotopes, Mars analogs

## Abstract

Lipid molecules are organic compounds, insoluble in water, and based on carbon-carbon chains that form an integral part of biological cell membranes. As such, lipids are ubiquitous in life on Earth, which is why they are considered useful biomarkers for life detection in terrestrial environments. These molecules display effective membrane-forming properties even under geochemically hostile conditions that challenge most of microbial life, which grants lipids a universal biomarker character suitable for life detection beyond Earth, where a putative biological membrane would also be required. What discriminates lipids from nucleic acids or proteins is their capacity to retain diagnostic information about their biological source in their recalcitrant hydrocarbon skeletons for thousands of millions of years, which is indispensable in the field of astrobiology given the time span that the geological ages of planetary bodies encompass. This work gathers studies that have employed lipid biomarker approaches for paleoenvironmental surveys and life detection purposes in terrestrial environments with extreme conditions: hydrothermal, hyperarid, hypersaline, and highly acidic, among others; all of which are analogous to current or past conditions on Mars. Although some of the compounds discussed in this review may be abiotically synthesized, we focus on those with a biological origin, namely lipid biomarkers. Therefore, along with appropriate complementary techniques such as bulk and compound-specific stable carbon isotope analysis, this work recapitulates and reevaluates the potential of lipid biomarkers as an additional, powerful tool to interrogate whether there is life on Mars, or if there ever was.

## 1. Introduction

The surface of Mars has been subjected to robotic exploration since July and August 1976, when two identical NASA landers under the *Viking* program attained the first ever successful soft landing on the Red Planet. Since then, Mars has motivated a process of continuous technological revitalization and as of today, there is a total of 14 missions (4 of them still operative) with objects on its surface (Huidobro *et al.*, 2022).

Although the *Viking* probes were directly targeted to find extant life, the increasing complexity of the scientific payloads over the years reflects an evolving suite of scientific goals from proof-of-concept payloads (Mars Pathfinder—Sojourner rover) to geochemical examinations of the surface (Phoenix lander and Mars Exploration Rovers [MER]—*Spirit* and *Opportunity*), including deep geological surveys (*Insight* probe) and eventually complex, multi-purpose astrobiological missions (Mars Science Laboratory—*Curiosity*, Mars 2020—*Perseverance* and Tianwen-1—*Zhurong* rovers).

The latter and the launch pending ExoMars *Rosalind Franklin* rover (Huidobro *et al.*, 2022) conclude the transition of astrobiology from an attempt to search for extant life to a more meticulous search for molecular evidence of ancient life. Tackling an interplanetary endeavor of this sort demands not only the exploration of regions of Mars that once held a window of habitability, but also the existence of recalcitrant biosignatures that can resist degradation by enduring hostile martian conditions and aided by mineral matrices that can enhance their preservation over time. Conveniently, lipids are among the most geostable biomolecules on Earth (Vinnichenko *et al.*, 2020), and so, they have the applicability and potential to meet these requirements.

At a first glance, however, Mars seems like a cold, barren, and apparently sterile planet with no magnetic field nor tectonic activity. Nonetheless, the exploratory analyses of the first landers yielded results that have progressively been disclosing extraordinary data regarding the planet's history and evolution. We now know that there are three very broad intervals that circumscribe the geological ages of Mars. First came the Noachian period (∼4.5 Gya), where evidence from the aforementioned missions pointed toward a world sculpted by liquid water and ice; with large lakes and a transient ocean in the Northern hemisphere accompanied by volcanic activity, a magnetic field, a thicker atmosphere, and ultimately a warmer climatology (Acuña *et al.*, 1998; Cabrol and Grin, 2001; Fairén *et al.*, 2003).

These conditions allowed us to envision the first ∼700–900 million years of martian history as the most habitable. The Noachian was followed by the Hesperian, or the snowball Mars (∼3.7–2.9 Gya) (Kereszturi, 2012; Michael, 2013), where the hydrosphere transitioned into a global cryosphere possibly due to the thinning of the magnetic field (Fairén *et al.*, 2003); however, volcanic activity was still present. In fact, the Olympus Mons volcano probably arose during this time (Fairén *et al.*, 2010).

Mars is currently at its Amazonian period (∼2.9 Gya to present), defined by the hyperarid and cold conditions that could render the planet completely devoid of any form of life, if there ever was. Nevertheless, microfossil remnants of such putative Noachian or Hesperian life could remain in certain martian refugia (caves, deep subsurface, salts, or ices) where conditions seem significantly more hospitable than average (Carrier *et al.*, 2020). It is now an acknowledged consensus in the space exploration community that the subsurface of Mars is one of the better targets for the search for extant or past life.

The highly oxidizing chemistry and the powerful ultraviolet (UV) incidence combined with the intense muon showers make the martian surface a true extreme environment like no other (Parro *et al*., 2011; Fernández-Remolar *et al*., 2013; Cheng *et al.*, 2017). Thus, penetrating a few centimeters into the martian subsurface appears to be a fundamental strategy in the search for signs of a given hypothetical life. The moderate tectonic activity and volcanism of the Amazonian period (compared with other periods), along with its low weathering and erosion rates (Carr and Head, 2010) are factors that may contribute to the stability and preservation of putative life and/or biosignatures over time.

### 1.1. Terrestrial analogs of Mars

Since crewed missions to Mars are not expected to be viable in the immediate future, human contribution to the field remains on terrestrial analogs. These are Earth environments that hold an array of geomorphological and geochemical characteristics that can be catalogued as analogous to past or present settings on Mars (Preston *et al.*, 2012). The early window of habitability that characterized the Noachian period implies the past existence of environments with liquid water that had potential to harbor putative martian life.

Examples of these environments include primitive hydrothermal systems (either subaerial or submarine) and subaqueous environments such as perennial and transient lakes, playa systems, deltaic formations, and iron-sulfate rich hot springs and shallow seas (Hays *et al.*, 2017). Eventually, the subsequent geological periods of Mars (*i.e.*, Hesperian and Amazonian) led to a global extremization of the conditions where perhaps only certain isolated niches could be deemed as habitable (Fairén *et al.*, 2010). Extreme environments of current Mars are cold and hyperarid, and they comprise subaerial systems such as iron-rich regolith, evaporitic deposits, or glaciers (polar caps of frozen carbon dioxide) that are exposed to high UV incidence, but also more protected subsurface environments such as water ice permafrost, frozen aquifers, deep igneous crust, lava tubes, and sedimentary deposits (Hays *et al.*, 2017). Terrestrial analogs of these martian environments must take into consideration the billion-year timescales that separate primitive and potentially habitable conditions from extreme Amazonian settings, and thus, the presence of geochemical/mineralogical features that favor the long-term preservation of biological fossils—or biosignatures—is fundamental, as the time for extant life on Mars has most likely already passed.

On Earth, most environments with analogy to past (hydrothermal and iron-sulfate rich systems) or present (hyperarid and hypersaline) settings on Mars are expected to host some degree of hostile physicochemical conditions to life, and although not always, terrestrial analogs are usually not well suited to harbor biological diversity. Instead, the prevalent form of life in these environments is extremophilic (*i.e.*, lovers of extreme conditions). Excluding certain species of extremophilic microorganisms that belong to the more biologically complex domain of Eukarya, most extremophiles fall in the domains of Bacteria and Archaea (Prokaryota).

Therefore, this work assembles lipid biomarkers produced in the framework of three main types of terrestrial analogs of Mars: hydrothermal, hypersaline, and acidic iron sulfate-rich environments, including polyextreme environments (combining several extreme conditions), as well as the geochemical suitability of these analogs to preserve biomolecules.

#### 1.1.1. Hydrothermal systems

These environments fall in the category of temperature extremes. Hot springs on Earth contain mineral deposits that are remarkably similar, for example, to the ones found on a formation of volcaniclastic origin on Mars called the Home Plate (Lewis *et al.*, 2008). This pentagon-shaped plateau is located by the Columbia Hills, a set of eroded mountains within the impressive 166 km-wide Gusev Crater, the best known example of ancient hydrothermal activity on Mars (Barbieri and Cavalazzi, 2014), and the landing site for one of the NASA MER *Spirit* in 2004.

Silica outcrops around the Home Plate have a characteristic nodular shape with millimeter-size, sponge-like protuberances known as digitate structures, whose morphology has a striking resemblance to those at El Tatio geyser field, a hydrothermal geyser system situated at 4320 meters above sea level (masl) in the Atacama altitude plains. Although digitate structures at El Tatio are mostly shaped by aeolian and geothermal environmental dynamics, microbial inputs might have had subtle templating effects in the resulting morphology (Gong *et al.*, 2022). On Mars, these structures are cautiously assumed to be a result of aeolian abrasion and hot spring discharge channels (Barbieri and Cavalazzi, 2014; Cady *et al.*, 2018; Ruff *et al.*, 2020).

#### 1.1.2. Aqueous acid systems

Also in 2004, the MER *Opportunity* landed on Eagle crater, in *Meridiani Planum*, a flat evaporitic site that might have formed due to an event of massive groundwater upwelling driven by the rise of the *Tharsis* bulge, a colossal volcanic plateau in the Western equatorial regions of Mars (Christensen and Ruff, 2004; Andrews-Hanna *et al.*, 2007). In other words, *Opportunity* landed in a system of plains that was likely flooded billions of years ago. Interestingly, *Meridiani Planum* is rich in iron and sulfate minerals, which suggests an ancient aqueous acidic system on Mars. An example of an appropriate analog site on Earth to *Meridiani Planum*, given its highly acidic conditions and mineralogy, would be the Río Tinto basin in Spain.

#### 1.1.3. Hyperarid and hypersaline

The Mars Science Laboratory *Curiosity* and the Mars 2020 *Perseverance* rovers landed at Gale and Jezero craters, respectively, and there is powerful evidence that proposes these sites as martian paleolakes with records of ancient aqueous environments (Schon *et al.*, 2012; Grotzinger *et al*., 2014; Vaniman *et al.*, 2014). In fact, recent data from *Perseverance* confirm the presence of influent valley networks toward Jezero crater, a delta with boulder conglomerates that advances into the lake and an outlet channel (Schon *et al*., 2012; Mangold *et al.*, 2021).

These dry basins, but especially Gale Crater, contain evaporitic deposits that suggest saline waters when they were once flooded. On Earth, this kind of environments are well characterized, and the literature describing microbial communities, lipid biomarkers, and stable carbon isotopic compositions in hypersaline environments is plentiful (Grice *et al*., 1998; Oldenburg *et al*., 2000; Antón *et al*., 2002; Jahnke *et al*., 2008, 2014; Ben-David *et al*., 2011; Crognale *et al*., 2013; Ziolkowski *et al.*, 2013; Wilhelm *et al*., 2017; Sánchez-García *et al*., 2018; Cockell *et al*., 2020; Carrizo *et al*., 2022), and a detailed description can be found in Section 3. This work will examine such studies in several aqueous hypersaline bodies, and some of them as notorious as the Dead Sea.

Then, the focus will shift into the transition of aqueous saline environments into drier evaporitic basins and eventually hyperarid deserts with subsurface halite units suggestive of past aqueous conditions, such as the Atacama or the Negev deserts. The former has long been a testing ground for scientific instrumentation related to planetary exploration (Moreno-Paz *et al.*, 2022). Layered outcrops in the Atacama have been scrutinized with laser Raman spectroscopy to inspect the analogous scenario that the ChemCam instrument from *Curiosity* would encounter by Aeolis Mons, the central peak within Gale Crater (Sobron *et al.*, 2013). Similarly, an emulator of the Raman Laser Spectrometer onboard the ExoMars *Rosalind Franklin* rover (Lopez Reyes *et al.*, 2019) was tested in the quartz- and phyllosilicate-rich region of Antofagasta (Veneranda *et al.*, 2021).

### 1.2. Other environments

This study covers terrestrial environments with specific analog counterparts on Mars that are—or have been—under robotic exploration. Hydrothermal systems, relict aqueous basins, hyperarid saline deposits, sulfur and iron-rich deposits, and acidic environments are only a subset of the analog settings that we can find on Earth. Other systems such as cold deserts, high-pressure submarine environments, or low-pressure environments are also relevant for biosignature identification in the framework of space exploration.

However, even though characterizing biosignatures from cold deserts and low-pressure systems is relevant and applicable to Mars, high-pressure settings and submarine environments are not. Instead, these remaining cold and/or marine environments where the pressure factor must be accounted for are suitable terrestrial analogs to study the icy worlds of the Solar System, which is a domain that extends beyond the scope of this review.

Meteorites, on the other hand, are peculiar microenvironments where hydrocarbons have been detected in the past (Nagy, 1966; Sephton *et al.*, 2001; Huang *et al.*, 2005). Regarding lipid-like molecules, the delivery of organic material within meteorites has oriented the discussion toward the study of terrestrial contamination on impact (Sephton *et al.*, 2001) and toward the likely abiotic generation of hydrocarbon chains in asteroids, comets, or dust under interstellar conditions (Huang *et al.*, 2005). Indeed, the focus of these studies lies on the discrimination between abiotic chain synthesis, where the carbons are incorporated one by one (McCollom *et al.*, 1999)—and enzyme-driven biosynthesis on Earth, where carbon atoms are added in pairs (Batsale *et al.*, 2021) generating a distinguishable lipid profile.

Despite the striking abundance of organic matter (whether biogenic or abiogenic) detected in certain carbonaceous meteorites, the microenvironments within these impactors are unlikely to have experienced a window of habitability comparable to that of Mars. For that reason, and due to the inescapable delving into the astrochemistry of the interstellar medium, the study of lipid biomarkers in meteorites is also considered to diverge from the main areas covered by this review, which is the molecular (lipid biomarkers) and geochemical (mechanisms of preservation) characterization of terrestrial analogs of Mars.

### 1.3. Lipid biomarkers

#### 1.3.1. Biosignatures and types of biomarkers

The interdisciplinary nature of astrobiology demands multiple perspectives when it comes to the characterization of terrestrial analogs such as El Tatio, Río Tinto, or Atacama, and among the many fields that intervene in the process, here we focus on the biological perspective. In particular, the search for biologically derived molecules, also known as biomarkers, has the diagnostic potential to indicate the presence of—most likely—microbial life.

This work discerns the concept of *biosignature* from *biomarker*; the former is a wider term defined as any measurable feature that indicates the presence of extant or extinct life (*e.g.*, isotopic composition, texture, morphological or structural patterns, or molecular distributions), whereas *biomarker* is a more restricted term used for specific biological molecules, which are, in turn, a particular (molecular) type of *biosignature*. There are several types of biomarkers that can be employed as targets, such as DNA, RNA, or proteins.

These biopolymers are useful when tracing their own biological source at a specific taxonomic level, but they are easily hydrolyzed macromolecules. Under ideal conditions, DNA alone cannot be kept intact more than 1 million years (Willerslev *et al.*, 2004) and polypeptide chains can resist up to ∼3.8 million years (Rybczynski *et al.*, 2013), which imposes severe limitations on their preservation and restricts the study of their ancient counterparts (Peterson *et al.*, 2007). This represents a meaningful drawback on Mars, where soil chemistry is highly oxidizing (Qu *et al.*, 2022) and the surface has been receiving a persistent UV incidence for billions of years (Cockell, 2000).

Biomolecules of lower molecular weight such as individual amino acids could be less prone to degradation, mostly shielded from radiation in the subsurface. Previous experimental work suggests that less complex amino acids such as alanine or glycine could, in theory, remain intact or at least detectable in the billion-year timescale (Kanavarioti and Mancinelli, 1990; Kminek and Bada, 2006). A disadvantage is that degradation into amines and short hydrocarbons would hinder the determination of isomerism to assess any potential biogenicity (l-amino acids vs. d-isomers of abiotic origin).

Although more limited in taxonomic information, the hydrocarbon cores of cell membrane lipids represent a powerful alternative for the search for extraterrestrial life due to their extreme recalcitrance, which is maximized if entombed within the sedimentary record. In fact, the oldest known biomarkers on Earth are of lipidic nature (hopanoids and isoprenoids) and are reported to be preserved within rock formations in basins of Northern Australia that are as old as 1.64 and 1.73 billion years (Brocks and Schaeffer, 2008; Vinnichenko *et al.*, 2020).

#### 1.3.2. Lipid classes and biosource allocation

*Lipid* is the general term used to refer to biogenic organic residues derived from cell membranes. They can represent up to 7% of the cell dry weight in microorganisms (Langworthy *et al.*, 1983), and they are involved in a number of functions in the cell (energy storage, transport of nutrients into the cell, stabilization of proteins, or maintenance of the proton-motive force), where the barrier function is central (Dowhan and Bogdanov, 2002; van Meer *et al.*, 2008).

In addition, lipids are also able to regulate membrane fluidity by modifying their polar head group or hydrophilic tail in response to external conditions such as temperature, pressure, or pH (*e.g.*, Russell *et al.*, 1995), or by adding rigidifying compounds such as hopanoids in the case of prokaryotes and steroids in the case of eukaryotes (Ourisson *et al.*, 1987). Structurally, they are composed of a hydrophilic (Greek: water-loving) polar head group and a hydrophobic (Greek: water-fearing) tail, where the head group is typically phosphate-based (although glycosidic-, amino-, or sulfate-based head groups are also found) (Hölzl and Dörmann, 2007) and the tail often consists of two hydrophobic chains connected to a glycerol backbone through ester and/or ether bonds.

Operationally, lipids are defined by their insolubility in water and solubility in non-polar solvents (Moss *et al.*, 1995). Thus, organic solvent-soluble molecules comprise a diverse suit of compounds from intact, complex molecules (*e.g.*, hopanoids, sterols, or pigments), to aliphatic chains resulting from the cleavage of the membrane tails. Based on this operational criterion, the main classes of lipid compounds are described next, in an ascending scale of complexity. Aliphatic hydrocarbons, on the other hand, are linear chains composed solely of carbon and hydrogen that can be saturated (*i.e.*, joined by single bonds) or unsaturated (*i.e.*, with double or triple bonds) and linear or branched (with or without alkyl branches, respectively). Although aliphatic hydrocarbons can be abiotically generated through Fischer-Tropsch reactions (Holm and Charlou, 2001), they can also be biogenic (Simoneit, 2002). They can be synthesized by microorganisms (from 0.005% to 2.69% of their dry mass) whose composition may be specific of certain physiological groups (Ladygina *et al.*, 2006).

For example, Cyanobacteria synthetize hydrocarbons through enzymatic modification of an elongated fatty acid (Coates *et al.*, 2014). Thus, hydrocarbons can be considered biomarkers according to a set of criteria such as the carbon chain length range, carbon number predominance, presence of functional groups, incorporation of double bonds, non-random methyl substitution, stereochemistry, or stable carbon isotope composition (Simoneit, 2002; Georgiou and Deamer, 2014). Overall, chains with dominance of odd or even carbons, presence of unsaturations and branching, or isotopic compositions depleted in ^13^C are common features of biogenic aliphatic hydrocarbons.

Straight (linear and saturated) hydrocarbons are known as *normal* alkanes (*n*-alkanes). Overall, shorter *chain n*-alkanes (*i.e.*, <20 carbons) with an even number of carbon atoms (except for the cyanobacterial C_17_ *n-*alkane) are normally associated to microbial inputs, whereas longer chains with an odd number of carbons are allocated to higher plants (Eglinton and Hamilton, 1967; Harwood and Russell, 1984; Killops and Killops, 2005; Derrien *et al.*, 2017). Alkanes containing one or more sets of double bonds (unsaturations) are called alkenes, and both *n*-alkanes and alkenes can substitute their hydrogens for methyl ramifications (-CH_3_ groups), which are consistent throughout this review. Accordingly, methylated alkanes, for example, can harbor one or more methyl substitutions (alkane types are depicted in Section 1 in the Supplementary Appendix). Chain lengths, degrees and sites of unsaturation, or number and positions of branches in the chain are properties related to phylogeny, microbial community structure, and adaptation to environmental constraints.

A hydrocarbon chain that incorporates a terminal carboxyl group (-COOH) is addressed as an alkanoic acid. Mid- and long-chain biosynthesized alkanoic acids are known as fatty acids, and they are also capable of incorporating methyl ramifications and unsaturations in their chains, which can, in turn, modify the straight conformation of the molecule. Similar to *n*-alkanes, those of shorter chain and even number of carbons are common in microbial membranes (Cranwell *et al.*, 1987; Kaneda, 1991; Pisani *et al.*, 2013), however, and unlike *n*-alkanes, long-chain fatty acids and fatty alcohols are associated to higher plants (Eglinton and Hamilton, 1967; Harwood and Russell, 1984; Killops and Killops, 2005; Derrien *et al*., 2017).

The ubiquity of fatty acids in the cell (either as structural “building blocks,” as part of neutral lipids serving as storage materials; or as derivatives involved in cell signaling) makes them suitable molecular traces of life. Moreover, if an alkanoic (or fatty acid) incorporates two terminal carboxyl groups, this will lead to the formation of dicarboxylic acids (see Section 2 in the Supplementary Appendix).

Similarly, hydrocarbons containing a hydroxyl group (-OH) are called alkanols (fatty alcohols), and polyhydric alkanols are compounds with two or more hydroxyl groups (diols, triols, *etc.*). A relevant triol involved in the formation of the basic structure of the membrane is glycerol. Its three hydroxyl groups form an ester with fatty acids forming a triglyceride, which is an amphipathic molecule containing a polar “head” (glycerol) and three apolar fatty acid “tails” (see Section 3 in the Supplementary Appendix). The polar head of a triglyceride can acquire more hydrophilic properties by binding more polar groups, such as phosphate groups (PO_4_^−^), forming an amphipathic phospholipid (Alberts *et al.*, 2002) with two hydrocarbon chains and reduced lipophilicity (more soluble in water and less soluble in non-polar solvents). These lipids are now conformed by hydrophilic moieties that interact with water and by hydrophobic moieties that self-associate, spontaneously forming lipid bilayers (Van Meer *et al.*, 2008). Thus, phospholipids function as monomers of biological membranes, the complex assembly that separates the cell from the outside environment (Ingólfsson *et al.*, 2014).

Some mid- and long-chain alkanes and alkenes (C_10–30_) display methyl branches at regular, repeating positions in the chain. These compounds are derived from a C_5_H_8_ unit with two unsaturations called an isoprene, and on the elongation of repeating isoprene units, the compounds that are formed are called isoprenoids (also known as terpenes or terpenoids), which are a large and diverse family of lipids (Holstein and Hohl, 2004 and references therein). Highly branched isoprenoid alkanes are commonly encountered in the domain of Archaea. Bacterial and eukaryotic membranes are composed of phospholipids whose fatty acid chains are linked by an ester bond to glycerol-3-phosphate, whereas archaeal membranes are composed of isoprenoid-based chains ether linked to glycerol-1-phosphate (Koga and Mori, 2005; Summons *et al.*, 2022). The core lipids within archaeal phospholipids are composed of two isoprenoid alkane chains linked to glycerol by two ether bonds, and thus, these are called dialkyl glycerol diethers (DGDs). The more extremophilic archaea (and some species of bacteria) contain long-chain membrane-spanning compounds that form a monolayer instead. The two ether bonds in either glycerol end grants glycerol dialkyl glycerol tetraethers (GDGTs) four ether bonds in total that increase the recalcitrance of these lipids. Isoprene and isoprenoids are depicted in Section 4 in the Supplementary Appendix.

The degradation of DGDs and GDGTs can yield linear hydrocarbon isoprenoids that can be allocated to archaeal species, however other linear isoprenoids stem from more complex lipid pigments. Chlorophyll, for example, has a phytol side chain that on hydrolysis can yield isoprenoid lipid biomarkers from photosynthetic bacteria, microalgae, or higher plants. Carotenoids (Section 5 in the Supplementary Appendix) are examples of isoprenoid pigments constituted by eight isoprene units formally derived from the C_40_ isoprenoid lycopene carbon skeleton that provide very distinct coloration to their multiple biological sources. Although more than 600 different carotenoid structures biosynthesized by photosynthetic (bacteria, eukaryotes, and halophilic archaea) and non-photosynthetic organisms have been identified (Eugster, 1995), most carotenoids lose their diagnostic value during alteration by reduction of all functional groups and generation of much less fossil-specific saturated hydrocarbons such as lycopane and β-carotane (Brocks and Summons, 2003).

Examples of early diagenetic reactions in pigment lipids and their products include (1) the expulsion of methylated aromatic rings (toluene and xylene) from the polyene (*i.e.*, polyunsaturated) chain, leading to long-chain linear isoprenoids that can, in turn, (2) cyclize into individual aromatic rings. Whether these reactions of ring expulsion and cyclization occur or not, the multiple unsaturations present in fresh diaromatic carotenoids make these molecules prone to (3) reduce their double bonds, and hence β-carotane becomes a common early diagenetic product of β-carotene (Koopmans *et al.*, 1997; Schaeffer *et al.*, 1997) with potential for effective preservation in the geological record. Due to their high molecular weight (HMW) and susceptibility to degrade at high temperatures, pigments need extraction techniques (mostly liquid chromatography) different from that employed for most of the lipid compounds described in this review (gas chromatography or GC) despite their common detection by mass spectrometry (MS). As this review aims at focusing mostly on the detection of lipid biomarkers using techniques reproducible on astrobiological missions such as GC-MS (*e.g.*, the SAM—Sample Analysis at Mars—instrument onboard the *Curiosity* rover), we will mainly focus on degradation products of some of the most relevant pigments throughout the manuscript.

Although this review does not delve into the larger, more complex lipid families, it is relevant to mention hopanoids, which are pentacyclic molecules with potential to be extensively functionalized and are widely spread intercalating and regulating the permeability of bacterial, fungal, and plant cell membranes, but not those of Archaea (Ourisson *et al.*, 1987). The fundamental pentacyclic hydrocarbon structure of hopanoids is based on the hydrophobic hopane skeleton (see Section 6 in the Supplementary Appendix), and its functionalized forms in bacteria are the amphipathic bacteriohopanepolyols (BHPs), which are saturated, pentacyclic terpanes of the C_31_–C_35_ extended hopane series with multiple hydroxyl groups. Thus, BHPs are biomarkers of the Bacteria domain and can act as membrane rigidifying components, a role fulfilled by sterols in the Eukarya domain. On diagenesis, BHPs lose hydroxyl groups, which yield recalcitrant homohopanes extended with a defunctionalized alkyl chain of varying carbon lengths (C_1_–C_5_).

All in all, there is a variety of lipid compounds that can be used as biomarkers with diagnosis capacity to identify sources, metabolisms, and environmental conditions. The combination of simpler and more complex lipids isolated from a given sample can be associated to more exclusive orders of organisms (*e.g.*, cyanobacteria, purple sulfur bacteria, green non-sulfur bacteria, sulfate-reducing bacteria [SRB], archaea, fungi, diatoms, *etc.*), but it will depend on the overall context where these are detected.

Still, broad distinctions such as prokaryote versus eukaryote, bacteria versus archaea, or bacteria versus algae, for example, are easily attainable (Brocks *et al.*, 2003; Meyers, 2003; Derrien *et al.*, 2017; Sánchez-García *et al.*, 2020b; Summons *et al.*, 2022). Thus, specific lipids and their ratios in membranes may be used as biomarkers to identify organisms (Brocks and Summons, 2003; Willers *et al.*, 2015), to study their adaptability and responses to environmental challenges (Cook and McMaster, 2002; Nichols *et al.*, 2004; Sturt *et al*., 2004; de Carvalho and Caramujo, 2018) and to disclose metabolic traits (van der Meer *et al*., 2003; Carrizo *et al*., 2022; Megevand *et al.*, 2022).

#### 1.3.3. Lipids in astrobiology: detection, preservation, and diagenesis

Lipids and hydrocarbons can be extracted from environmental samples as diverse as soil (Amelung *et al.*, 2008; Tinoco *et al.*, 2018; Vega-García *et al.*, 2021), sediment (Sánchez-García *et al*., 2008; Carrizo *et al.*, 2019a), ice, snow (Nemirovskaya, 2006), particulate organic matter (Vonk *et al.*, 2010; Okuno and Yokomizo, 2015), biofilms and microbial mats (Sánchez-García *et al.*, 2020a; Megevand *et al.*, 2022), or even meteorites (Nagy, 1966; Sephton *et al.*, 2001; Huang *et al*., 2005).

While isolation and extraction methods vary, analysis and visualization are usually carried out via GC-MS. Even though it is not the scope of this review to delve into the technical properties of lipid biomarker analysis, it is of great relevance to explain one of the most utilized complementary techniques: the compound-specific isotopic analysis of individual lipid biomarkers. Stable carbon isotopic studies are based on elucidating the proportion of the naturally occurring heavier carbon isotope (^13^C; 1.11%) to the lighter, most abundant isotope (^12^C; 98.89%) in the total organic carbon (bulk) or in a specific compound. During growth, autotrophic organisms fix inorganic carbon from the atmosphere favoring the incorporation of the lighter isotope over the heavy one, since the former is more energetically preferable. This results in ^13^C-depleted biomass relative to inorganic substrate. Such depletion—or fractionation—can be interpreted as a biosignature of one metabolic route of carbon fixation over another (van der Meer *et al.*, 2003). Thus, the carbon isotope composition of individual compounds in conjunction with their molecular analysis facilitates the allocation of a biogenic or abiogenic origin with a significantly increased accuracy.

Lipid biomarker and stable carbon isotopic analyses have long been utilized in environmental science to determine the source of organic matter in active systems (ecological context) and in the fossil record (paleobiological context), as well as to elucidate biogeochemical processes and the environmental changes involved (Eglinton and Murphy, 1969; Meyers and Ishiwatari, 1993). The utility of lipid biomarkers as indicators of biological, paleoenvironmental, and geochemical processes on Earth has also been applied to astrobiology, where numerous studies have exploited the chemical resistance, source-diagnosis potential, and unique insights that lipids offer to search for extant life in extreme environments with martian analogy.

Mars analogs on Earth are attractive not only because of the extremophilic life they harbor, but also due to a number of environmental peculiarities (*e.g.*, particular mineralogy, abundance of salts, scarcity of liquid water, extremely low temperature, *etc.*) that may favor the long-term preservation of lipids by mineral encapsulation or entombment, which further fosters the resistance of the molecule to degradation and multiplies its astrobiological value.

The preservation of lipids is a pivotal matter in this review, as fresh and functionalized biomolecules seem unlikely targets in planetary exploration. A focus on preservation, however, demands a focus on *diagenesis*, which can be defined as the alteration of organic matter through biological or chemical processes as it is transported by water or on deposition in sediments (Brocks and Summons, 2003). Examples of alterations to lipid molecules include hydrolysis, oxidation, reduction, sulfurization, desulfurization, or atomic rearrangements (Brocks and Summons, 2003) that degrade the molecule to a simpler, defunctionalized form (ultimately insoluble linear hydrocarbons or *n*-alkanes). These diagenetic reactions occur “early” (over geologic timescales) after the lipids disassociate from the membrane and depending on the level of modifications (which, in turn, depends on the elapsed time and conditions of deposition), these “simplified” lipids can still inform about their biological sources. Such will be the case for the terrestrial analog environments presented here, which are considered geologically modern (maximum a few million years).

If the conditions to preserve organic matter are suboptimal or if the environment is ancient in million-to-billion-year timescales, we are shifting into a process of *catagenesis*, where increased burial correlates with increased temperatures and pressures, eventually breaking C-O and C-C bonds leading to hydrocarbon fragmentation or cracking (Brocks and Summons, 2003). In the absence of burial but under intense UV and cosmic radiation for billions of years, the detection of C_10_–C_12_ *n*-alkanes by *Curiosity* on Mars could be an example of multiple processes involved in molecular degradation where putative long-chain *n*-alkanes or alkanoic acids of uncertain origin ended up being fragmented (Freissinet *et al.*, 2019). Beyond catagenesis, temperatures and pressure trigger host rock metamorphism and hydrogen is released from hydrocarbons, leaving simple molecules such as methane and initiating the aromatic carbon phase (polycyclic aromatic hydrocarbons) in a terminal process of molecule degradation known as *metagenesis* (Brocks and Summons, 2003).

In the context of early diagenesis, part of this study focuses on the simplest aliphatic lipids, not only fatty acids, but also alkanols and defunctionalized alkanes, the latter being the most stable group of hydrocarbons. After billions of years of extreme conditions on Mars, the chances of identifying intact, complex molecules are minimal. In addition, they must be detectable by current instrumentation (GC-MS) reproducible in martian rovers (SAM and the Mars Organic Molecule Analyser [MOMA] from the ExoMars *Rosalind Franklin*). Notwithstanding, other lipid compound classes with a more complex chemistry such as pentacyclic triterpenoids or polyether, membrane-spanning lipids are also considered. This is not only due to their differential preservation potentials in any given lithologies, but also due to their particular taphonomic and diagenetic evolution. Finally, the review also focuses on the simplest forms of life (*i.e.*, prokaryotes). Given their abundance, adaptability, and resilience on virtually every environment, they are the most likely candidate form of life (if any) to be found on Mars.

**FIG. 1.**
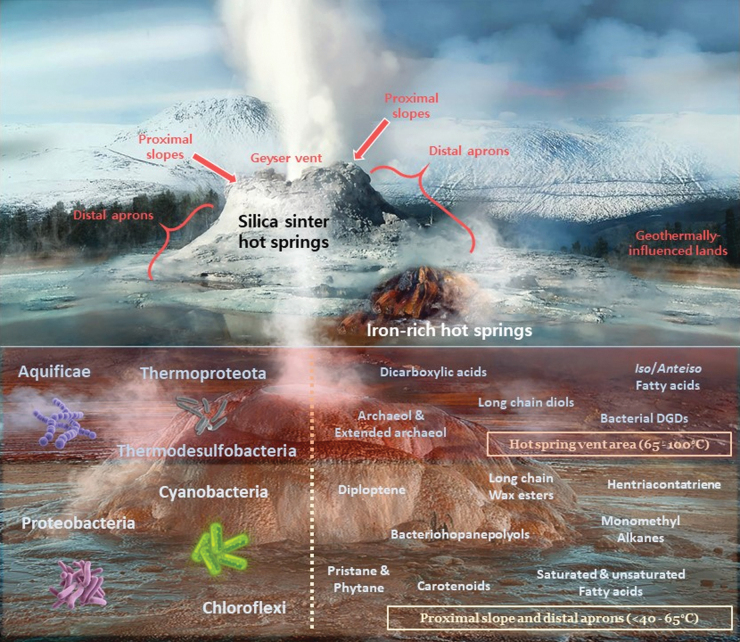
Recreation of the lipid biomarker distribution in hydrothermal systems, where characteristic hot springs, fumaroles, and active geysers are represented. The upper section illustrates two main types of hot spring mounds based on mineralogy: silica sinter (white, erupting geyser) and iron-rich (smaller brown mound to the lower right). The main sections of a geyser are indicated in the white silica sinter mound: geyser vent, proximal slopes (immediate slopes adjacent to the vent), distal aprons (silicified facies away from the geyser slopes), and the further geothermally influenced lands; where other aprons, mid-apron pools, and hot springs are found. The lower section focuses on the lipid molecules (right side) and associated microbiological sources (left side) found in the hotter (65–100°C) regions of a geyser (vent and hot proximal slopes; reddish rectangle) and in the milder (<40–65°C) distal slopes and aprons (pale yellow rectangle). The high temperatures in these environments demand the presence of thermophiles and thermotolerant members of both the Archaea (Thermoproteota) and Bacteria (rest of phyla displayed) domains. To avoid figure cluttering, only some of the most notable lipid biomarkers in hot spring environments have been included. Image designed by Iratxe de Dios with images from Castle Geyser in YNP (white silica sinter mound), from the Chocolate Pots in YNP (smaller brown mound), and from El Tatio Geyser Field (silica sinter mound in the background of the lower section of the figure). YNP, Yellowstone National Park. Image edited and labeled by main author (P.L.F.). 70 × 60 mm (300 × 300 DPI).

**FIG. 2.**
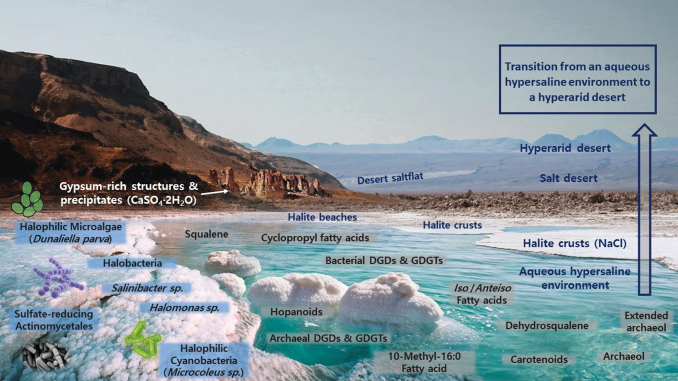
Recreation of lipid biomarkers distribution in hypersaline environments. Aqueous hypersaline bodies subjected to high evaporation rates and conditions of aridity have a propensity to transition into dry salt flats, evaporitic deserts (salt deserts rich in subsurface halite units), and, ultimately, into hyperarid deserts (extremely low water activity). This environmental shift is depicted in the figure. The halite crusts and mounds accumulated in the coastal sections around hypersaline water bodies are eventually covered by layers of dust due to aeolian and sedimentary processes. Subsequent aeolian abrasion and further desertification leaves subsurface halite units of varying depths and thicknesses under the newly generated desert. The presence of other salt microniches or even macroscopic structures such as gypsum-rich precipitates is often common. This figure illustrates groups of lipid biomarkers encountered in hypersaline and hyperarid environments (water side) besides some of the most frequent halophilic microorganisms that inhabit these settings (salt crust side). To avoid figure cluttering, only some of the most notable lipid biomarkers found in hypersaline and hyperarid environments have been included. Image designed by Iratxe de Dios using images from the Dead Sea (Jordan/Israel) for the aqueous sections and the salt crusts, as well as a mountain (left side) from the evaporitic and hyperarid halite Sdom Formation by the Dead Sea coasts. The background desert is a section of the Atacama region escorted by Andean mountains and volcanoes. Image edited and labeled by main author (P.L.F.).

**FIG. 3.**
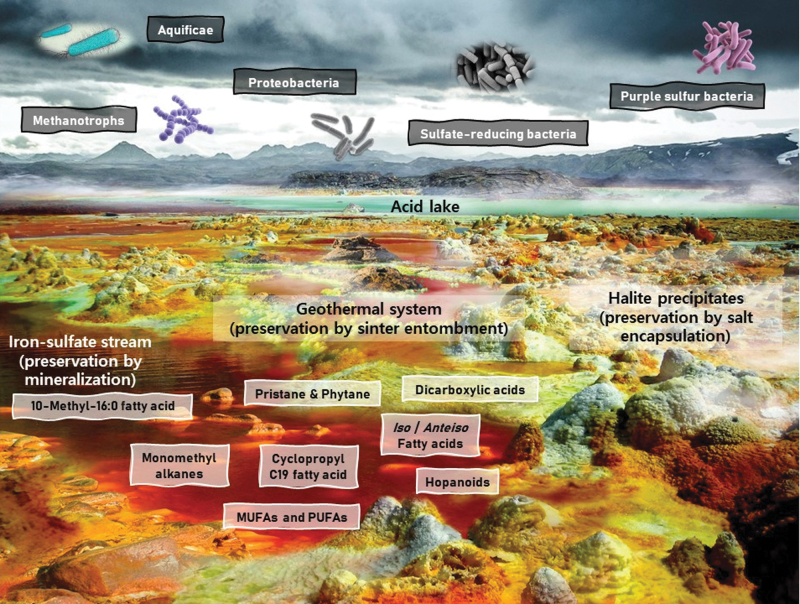
Recreation of a polyextreme environment including an iron-sulfate stream, a geothermal system, an acidic lake, and halite precipitates. Despite being the most hostile environments on Earth, polyextreme conditions may harbor multiple mineral matrices that favor lipid preservation to favorable extents. Examples of preservation strategies in this figure include sinter entombment, halite encapsulation, and mineralization within metal-rich phases, such as iron. The latter makes the Río Tinto iron-rich basin in Spain a key astrobiological target and a well-established martian analog, whereas the rest of the preservation strategies are likely to take place at the Dallol hydrothermal dome in Ethiopia. These “alien-like” landscapes truly interrogate the limits of microbial adaptability and at the same time emulate intemperate conditions that may have been present on early Mars. To provide a comprehensive overview, only some of the most relevant lipids (red aqueous stream) as well as common acidophiles (cloudy sky) that thrive in highly acidic and polyextreme environments have been included. Image designed by Iratxe de Dios using images from Río Tinto (Spain) for the iron sulfate-rich stream and images from the Dallol hydrothermal system in the Danakil Depression (Ethiopia). Image edited and labeled by main author (P.L.F.).

The review published by Summons *et al.* (2022) on fossilized lipid biomarkers as molecular tools to elucidate Earth's paleobiological evolution is an ideal introduction to the field of lipid biomarkers in the highly relevant contexts of geochemistry and geomicrobiology. This review builds on such work and hopes to establish a natural progression by providing an astrobiological perspective and a thorough recapitulation that gathers most of the studies employing lipid biomarker analyses on terrestrial analogs of Mars. We, therefore, report prominent lipid biomarkers and common profiles in several extreme conditions to unearth a suit of traceable molecular and isotopic biosignatures that we aim at summarizing after each environment, in Tables 1–3 and Figs. 1–3. Perhaps, these biosignatures are the key to identify and catalog characteristic microbial patterns in terrestrial environments that can be used as a reference tool for future planetary-class exploration missions to Mars and other potentially habitable worlds of the Solar System.

**Table 1.** Hydrothermal Systems

Lipid biomarkers	Potential biosource	Compound-specific δ^13^C isotopic composition	References and terrestrial analog
Branched GDGTs	Thermophilic bacteria	NR	Kaur *et al.* (2015); Pancost *et al*. (2005); OK, TVZ, NZ
Isoprenoidal GDGTs	Thermophilic archaea	NR	Kaur *et al*. (2015); Pancost *et al*. (2005); OK, TVZ, NZ
Branched DGDs (C_16_–C_18_)	Thermophilic bacteria *Thermodesulfobacterium commune* *Ammonifex degensii*	NR	Jahnke *et al*. (2001); Zeng *et al*. (1992a); OS, YNP
Archaeol	Thermophilic archaea	−13.6‰ to −26.7‰	Kaur *et al*. (2015); Pancost *et al.* (2005); OK, TVZ, NZ Schubotz *et al.* (2013); Lower Geyser Basin, YNP
Long chain diols (C_18_–C_23_)	Chloroflexi *Thermomicrobium* sp.	NR	Pond *et al*. (1986); Zeng *et al*. (1992a); OS, YNP
Short MUFAs 20:1ω11 and 20:1ω1	Thermophilic bacteria Aquificales	−18.4‰ to −30.6‰ (OS) +4.4‰ to −9.8‰ (BP)	Jahnke *et al*. (2001); OS, YNP Schubotz *et al*. (2013); BP, YNP
Cyclopropyl C_21_ fatty acid	Thermophilic bacteria Aquificales	−23.4‰ to −25.2‰ (OS) +2.2‰ to −14.0‰ (BP) +3.9‰ (OK)	Jahnke *et al*. (2001); OS, YNP Schubotz *et al.* (2013); BP, YNP Pancost *et al.* (2005); OK, TVZ, NZ
Dicarboxylic acids (C_6_–C_15_)	Thermophiles (bacteria or archaea)	NR	Sánchez-García *et al.* (2020a); Hveragerdi, Iceland Sánchez-García *et al*. (2019); El Tatio Geyser Field, Chile
*Iso*-C_15_, C_17_, C_18_ and C_19_ *Anteiso-*C_15_, C_17_ and C_18_ fatty acids	Thermophilic sulfate-reducing bacteria For example, *T. commune* *Thermocrinis ruber*	*Iso_(15–19)_*: −24.2‰ to −28.6‰ *Anteiso_(15–18)_*: −26.0‰ to −28.0‰	Jahnke *et al*. (2001); OS, YNP Sánchez-García *et al.* (2020a); Hydrothermal Iceland
Pristane and phytane	Cyanobacteria, Chloroflexi, green/purple sulfur/non-sulfur bacteria, archaea, algae, and/or plants	Pristane: NR Phytane: −27.8‰ (OS) +0.1‰ to −23.9‰ (BP)^a^	Parenteau *et al*. (2014); Chocolate Pots, YNP Schubotz *et al.* (2013); OS, BP, YNP Sánchez-García *et al*. (2019); El Tatio Geyser Field, Chile
Diploptene	Cyanobacteria (if accompanied by other cyanobacterial biomarkers)	NR	Sánchez-García *et al.* (2020a); Hydrothermal Iceland
Hentriacontatriene (C_31:3_)	Chloroflexi	−11.3‰ to −21.4‰^b^	Kaur *et al.* (2015); Pancost *et al.* (2005); OK, TVZ, NZ van der Meer *et al*. (2008); Hveravellir, Iceland Megevand *et al*. (2022); El Tatio Geyser Field, Chile
C_31_–C_37_ wax esters	Chloroflexi *Chloroflexus* sp.	−11.0‰ to −24.0‰^b^	
C_38_–C_40_ wax esters	Chloroflexi *Roseiflexus* sp.	NR	
Cyclopropyl C_17_ and C_19_ fatty acids	Gram-negative proteobacteria and/or anaerobic bacteria Purple and green sulfur bacteria	*cy*-C_17_: −24.4‰ *cy*-C_19_: −23.2‰ to −24.0‰	Jahnke *et al*. (2001); Pancost *et al*. (2005); Sánchez-García *et al.* (2020a); Sánchez-García *et al.* (2019); most locations
MUFAs and PUFAs (16:1ω7, 18:1ω7, 18:1ω9, 18:2ω6, 18:3ω3, 18:3ω6)	Cyanobacteria *Oscillatoria*, *Mastigocladus*, *Phormidium*, *Fischerella* and *Synechococcus* sp. (BHPs and carotenoids together are ubiquitous in multiple phototrophic bacteria)	−4.0‰ to −25.8‰	Sánchez-García *et al.* (2020a); Hydrothermal Iceland Megevand *et al*. (2022); El Tatio Geyser Field, Chile
Heptadecane (C_17_)		−22.9‰ to −32.4‰	Jahnke *et al*. (2004); OS and Fountain Paint pots, YNP Sánchez-García *et al*. (2020a); Hydrothermal Iceland
Octadecene (C_18:1_)		−17.9‰	Megevand *et al.* (2022); El Tatio Geyser Field, Chile
Monomethyl alkanes (C_15_-C_19_ and C_20_-C_22_)		−17.6‰ to −32.6‰	Sánchez-García *et al*. (2020a); Hydrothermal Iceland Megevand *et al*. (2022); El Tatio Geyser Field, Chile Jahnke *et al*. (2004); OS and Fountain Paint Pots, YNP
Dimethyl alkane (7,11-Dimethyl C_17_)		−29.4‰ to −31.4‰	Jahnke *et al*. (2004); Shiea *et al*. (1990); van der Meer *et al.* (2000); OS and Fountain Paint Pots, YNP
BHPs		−17.1‰ to −29.9‰^c^	Jahnke *et al*. (2004); Summons *et al.* (1996); OS and Fountain Paint Pots, YNP
Carotenoids (β-carotene, γ-carotene, zeaxanthin, canthaxanthin, echinenone and myxoxanthophyll)		NR	Fecteau *et al.* (2021); Hirschberg and Chamovitz (1994); multiple locations at YNP
Other carotenoids (Chlorobactene and isorenieratene)	Green sulfur bacteria	NR	Wahlund *et al.* (1991); North Island, TVZ, NZ
Farnesol	Green and purple sulfur bacteria	−12.0‰ to −22.0‰	Manske *et al*. (2005); Black Sea van der Meer *et al*. (1998)

^a^ The wide range of δ^13^C values in Bison Pool reported by Schubotz et al., (2013) indicated contribution of different phototrophs with different carbon fixation pathways (i.e., Calvin cycle, 3HP bicycle and/or rTCA pathway).

^b^ The most depleted δ^13^C values may not represent typical carbon assimilation fingerprints by Chloroflexi (i.e., using the 3HP bicycle) due to a process of cross-feeding (photoheterotrophy) while coexisting with a cyanobacterial mat.

^c^ BHP methylated and non-methylated products.

BHP = bacteriohopanepolyol; BP = Bison Pool; DGD = dialkyl glycerol diether; GDGT = glycerol dialkyl glycerol tetraether; NR = not reported; MUFA = monounsaturated fatty acid; OK = Orakei Korako; OS = Octopus Spring; PUFA = polyunsaturated fatty acid; TVZ = Taupo Volcanic Zone; YNP = Yellowstone National Park.

**Table 2.** Arid and Hypersaline Environments

Lipid biomarkers	Potential biosource	References and terrestrial analog
Isoprenoidal GDGTs Crenarchaeol Caldarchaeol	Halophilic archaea	Jahnke *et al.* (2008); Guerrero Negro, Baja California
Archaeol	Halophilic archaea *Halorubrum*, *Haloferax* and *Haloarcula*	Stiehl *et al.* (2005); Dead Sea, Israel Cheng *et al.* (2017); Dalangtan Playa, Tibet, China
Extended archaeol	Halophilic archaea or non-halophilic methanogens	Wilhelm *et al*. (2017); Yungay halite unit, Atacama
Squalene	Halophilic archaea	Jahnke *et al.* (2008); Guerrero Negro, Baja California Stiehl *et al.* (2005); Dead Sea, Israel Grice *et al*. (1998); Sdom Formation, Israel Sánchez-García *et al*. (2018); Salar Grande, Atacama
Dehydrosqualene	Halophilic archaea	Jahnke *et al.* (2008); Guerrero Negro, Baja California
Crocetane	Halophilic archaea	Jahnke *et al*. (2008); Guerrero Negro, Baja California Sánchez-García *et al.* (2018); Salar Grande, Atacama
Gammacerane	Purple sulfur bacteria *Rhodopseudomonas palustris* Protozoa Bacterivorous ciliates Paleosalinity indicator	Kleemann *et al.* (1990); Sinninghe Damsté *et al.* (1995); Summons *et al*. (1988)
Cyclopropyl C_17_ and C_19_ fatty acids	Gram-negative proteobacteria and/or anaerobic bacteria Halophilic purple sulfur bacteria Physiological stress indicator	Fourçans *et al*. (2004); Rontani and Volkman (2005); Camargue Coast, France Sachse and Sachs (2008); Christmas Island Crognale *et al.* (2013); Mierlei Lake, Romania Ben-David *et al.* (2011); Negev Desert, Israel Shen (2020); several regions in Atacama
*Iso*/*Anteiso*-C_15_ and C_17_ fatty acids	Gram-positive bacteria Sulfate-reducing bacteria	Crognale *et al.* (2013); Mierlei Lake, Romania Wilhelm *et al.* (2017); Yungay gypsiferous unit, Atacama Sánchez-García *et al.* (2018); Salar Grande, Atacama
MUFAs (18:1ω9/11/12, 19:1ω6/8)	Alphaproteobacteria	Jahnke *et al.* (2014); Guerrero Negro gypsum crusts, Baja California
PUFAs (18:2ω6, 18:2ω9)	Algae and other eukaryotic sources *Dunaliella parva*	Stiehl *et al.* (2005); Dead Sea, Israel Shen (2020); several regions in Atacama
MUFAs and PUFAs (16:1ω7, 16:1ω9, 18:1ω7, 18:1ω9, 18:2ω6, 18:2ω7)	Cyanobacteria Halotolerant *Microcoleus vaginatus* and endolithic *Chroococcidiopsis* sp.	Jahnke *et al*. (2014); Guerrero Negro gypsum crusts, Baja California Dembitsky *et al.* (2001); Negev Desert, Israel Sánchez-García *et al.* (2018); Salar Grande, Atacama Ziolkowski *et al.* (2013); Gypsum precipitates, Atacama
10-Methyl-16:0 and 10-Methyl-18:0 Fatty acids	Halotolerant sulfate-reducing Actinomycetales	Jahnke *et al*. (2014); Guerrero Negro gypsum crusts, Baja California Crognale *et al.* (2013); Mierlei Lake, Romania
Monomethyl alkanes (C_15_–C_19_)	Cyanobacteria	Sánchez-García *et al*. (2018); Gypsum and halite units of Salar Grande, Atacama Azua-Bustos *et al.* (2020); Yungay smectite unit, Atacama
Phytosterols β-Sitosterol and stigmastanol	Land plants	Sánchez-García *et al.* (2018); Gypsum and halite units of Salar Grande, Atacama
Hopane and homohopanes of the C_30_–C_34_ series	Bacteria	Sánchez-García *et al.* (2018); Gypsum and halite units of Salar Grande, Atacama
Carotenoids β-Carotene and isorenieratene	*Salinibacter ruber* and Green sulfur bacteria	Antón *et al*. (2002); Hopmans *et al.* (2005); Saltern ponds/crusts in Alicante and Mallorca, Spain

**Table 3.** Acid and Iron Sulfate-Rich Environments

Lipid biomarkers	Potential biosource	References and terrestrial analog
Cyclopropyl C_19_ fatty acid	Gram-negative proteobacteria and/or anaerobic bacteria Purple sulfur bacteria	Tan *et al*. (2018); Microbial mat at St. Oswald's Bay, United Kingdom
*Iso*/*Anteiso*-C_15_ and C_17_ fatty acids	Sulfate-reducing bacteria	Sánchez-García *et al*. (2020b); concentration increased with depth at Río Tinto Tan *et al*. (2018); St. Oswald's Bay, United Kingdom Parenteau *et al.* (2014); Chocolate Pots, YNP
*Iso*-C_18_ fatty acid	Chloroflexi	Carrizo *et al.* (2019b); Dallol hydrothermal dome, Ethiopia
MUFAs and PUFAs (16:1ω7, 16:1ω9, 18:1ω7, 18:1ω9, 18:1ω10, 18:2ω6, 18:2ω7)	Cyanobacteria, Aquificales, and Thermotogales (Thermophiles)	Sánchez-García *et al.* (2020b); concentration increased with depth at Río Tinto Tan *et al.* (2018); St. Oswald's Bay, United Kingdom Parenteau *et al.* (2014); Chocolate Pots, YNP Johnson *et al.* (2020); Hyperacid lakes, Australia Carrizo *et al.* (2019b); Dallol hydrothermal dome, Ethiopia
MUFA 18:1ω8	Methanotrophic bacteria	Sánchez-García *et al.* (2020b); concentration increased with depth at Río Tinto
10-Methyl-16:0 fatty acid	Acidophilic sulfate-reducing bacteria	Tan *et al.* (2018); St. Oswald's Bay, United Kingdom Parenteau *et al.* (2014); Chocolate Pots, YNP
Dicarboxylic acids (C_6_–C_15_)	Thermophiles	Sánchez-García *et al.* (2020b); 40–100 cm of depth in Río Tinto Carrizo *et al.* (2019b); Dallol hydrothermal dome, Ethiopia
C_17_ Monomethyl and dimethyl alkanes	Cyanobacteria	Parenteau *et al.* (2014); Chocolate Pots, YNP Carrizo *et al.* (2019b); Dallol hydrothermal dome, Ethiopia
Pristane and phytane	Phototrophs Cyanobacteria Chloroflexi or Archaea	Carrizo *et al.* (2019b); Dallol hydrothermal dome, Ethiopia
>C_20_ fatty acids (odd-over even pattern)	Algae, fungi and higher terrestrial plants	Johnson *et al.* (2020); Australia
Phytosterols β-Sitosterol and stigmastanol	Land plants	Carrizo *et al.* (2019b); Dallol hydrothermal dome, Ethiopia Sánchez-García *et al.* (2020b); Río Tinto

### 1.4. Lipid nomenclature

A common shorthand nomenclature is used throughout the review to designate aliphatic lipid molecules: The alkyl chain carbon number (*x*) is represented as C_x_; straight ad saturated (*normal*) chains as *n*-C_*x*_; methyl-branched as br-C_*x*_; mono-, di-, or trimethyl alkanes as Me-C_*x*_, DiMe-C_*x*_, or TriMe-C_*x*_, and generally abbreviated as MMA, DMA, and TMA, respectively. *Iso-* (2-methyl relative to methyl terminus) or *anteiso*- (likewise 3-methyl) chains as *i*-C_*x*_ and *a*-C_*x*_, respectively. Cyclopropyl fatty acids as *cy*-C_*x*_; unsaturated chains as C_*x*:*y*_, where *y* is the number of double bonds or unsaturations.

Finally, unsaturated fatty acids are represented as *x*:*y*ω*z*, where (*z*) denotes the specific position of the first double bond under the frame of the (ω) notation (*i.e.*, starting to count from the methyl end of the acid). In this last scenario, (*x*) is assumed to be the number of carbon atoms and so, annotating (*C*) in front of (*x*) is not necessary for fatty acids. Monounsaturated and polyunsaturated fatty acids are abbreviated as MUFA and PUFA, respectively. Examples of the most relevant lipid compounds described here are listed in the Supplementary Appendix, together with their chemical structure.

## 2. Hot Spring Environments

Among the various extreme environments that this review aims at covering and due to their many implications in astrobiology and on the probable emergence of life on early Earth, hot springs are a mandatory first stop. Hydrothermal fields and hot spring deposits are primary targets in the search for fossil evidence of life on Earth and beyond for a number of reasons. First, they provide a thermodynamically favorable setting for the synthesis of simple organic compounds that need to occur at high temperatures (Farmer, 2000), as well as accomplishing a series of environmental constrains such as interacting interfaces that promote the accumulation and polymerization of prebiotic compounds (Damer and Deamer, 2015; Deamer and Georgiou, 2015).

In other words, hydrothermal systems might have been a spot for life to emerge. Second, these environments are characterized by unprecedented rates of mineralization, which directly favors microbial entombment and subsequent fossilization, making such deposits rich storehouses of paleobiological information (Fernandez-Turiel *et al.*, 2005; Parenteau *et al.*, 2014; Campbell *et al*., 2015; Williams *et al.*, 2021). Third, the emergence of hydrothermal activity seems to be inextricably linked to planetary formation and evolution (Holm and Andersson, 2005). Beyond Mars, the icy satellites of the outer Solar System are already under close scrutiny for future missions given their potential for hydrothermal activity under their putative subglacial oceans (Lowell, 2005; Howell and Pappalardo, 2020; Cable *et al.*, 2021).

### 2.1. Hot spring geochemistry and mineralogy

The microbial diversity in subaerial hydrothermal systems is substantial, and it thrives in very intemperate conditions: high temperatures (80–110°C) that fluctuate more than 30°C in just 10 cm, pH variations from <1 to >7, and rapid hydration-dehydration cycles (Dunckel *et al.*, 2009; Damer and Deamer, 2015; Kaur *et al.*, 2015). This results in multiple microbial niches with highly discernable patterns where, for example, thermophilic microorganisms (>80°C) can steeply grade into mesophiles (15–40°C) in the span of 1 m (Dunckel *et al.*, 2009).

Thus, this article will be structured based on the cross-section of a typical hydrothermal system on Earth. That is, the most pertinent lipid biomarker findings will be summarized by following: a thermal gradient that begins at extreme near-vent and proximal slope settings of a geyser (ranging 65–100°C), continues at mid-apron surfaces and hot pools (40–65°C), and concludes in the cooler distal aprons of geothermally influenced lands (<40°C) (Campbell *et al.*, 2015), as reflected in Fig. 1 (top half). The diverse typology of hot springs depends to a large extent on the chemical composition of the hot fluids and the surrounding mineralogy. Even though they all require percolating groundwater flowing through the porous igneous rock near a magma chamber, it is the composition of the rising superheated fluids and the spring deposit environment what determines their differences (Kresic, 2010), which can be reflected in a wide range of pH values.

Certain hot springs may present very low pH values, such as the acid-sulfate springs of Dallol, Ethiopia (Carrizo *et al*, 2019b). Others display almost neutral values, such as numerous alkaline chloride springs from Yellowstone National Park (YNP) that are saturated with silica (Fishbain *et al*., 2003; Bowen De León *et al.*, 2013; Churchill *et al.*, 2021). Finally, other hydrothermal sites can be mildly alkaline, forming bicarbonate springs saturated with carbon dioxide and carbonate minerals such as the gypsum-rich Chongqing hydrothermal area in Southwestern China (Ta *et al.*, 2019).

There are also cases of iron-rich springs with abundant dissolved iron, such as the Chocolate Pots in YNP (Parenteau *et al.*, 2014). As expelled hydrothermal fluids cool down, mineral species alter their structures on contact with the atmosphere. For example, in acid-sulfate springs, the venting acid fluids oxidize H_2_S from volcanic vapors to form H_2_SO_4_ (sulfuric acid) as gases ascend toward the O_2_-rich surface, which, in turn, contributes to modify mineral structures by acid leaching. Other examples would be Fe^2+^ oxidation to Fe^3+^, which forms iron lumps around hydrothermal vents, or a dynamic combination of evaporation and cooling of hydrothermal fluids supersaturated in carbonates or silica. These last two trigger the precipitation of carbonate (travertine) or silica (geyserite) sinter deposits via heterogeneous and homogeneous nucleation and polymerization (Des Marais and Walter, 2019).

Silica sinter is highly relevant to astrobiology given its high preservation potential and its screening properties against UV radiation, and it consists of a hydrated, amorphous form of non-crystalline silica called opal-A. Once the superheated and silica-rich fluid reaches the surface, it cools down, precipitating as amorphous silica sinter that solidifies, forming a macroscopic geyserite mound (Lynne *et al.*, 2005). At this stage, microbial biosignatures such as lipid biomarkers become entombed and silicified (Cady *et al.*, 2018), providing a notable paleobiological and paleoenvironmental record on extraction from geyserite.

Contemporary terrestrial spring deposits such as those in YNP (United States), Iceland, New Zealand, or El Tatio geyser field (Chile) are some of the best characterized hydrothermal environments on the planet in terms of lipid biomarker and carbon isotopic composition. Their unique features offer an opportunity to loom not only to an ancient, hydrothermally active Mars (Summons *et al.*, 1996; Pinti, 2011), but also into our primitive world. Related to this aspect is the fact that on-land hydrothermal systems are gaining increasing popularity as plausible candidate environments to support the origin of life on Earth. Given the propensity of deep-sea vents to dilute decisive prebiotic agents and hence delay any further polymerization, it is hard to ignore the way subaerial hydrothermal fields offer a unique and interrelated atmosphere-hydrosphere-lithosphere interface. These fluctuating interfaces allow for dehydration-rehydration cycles that, in turn, favor the accumulation and self-assembly of complex organic compounds while providing an energetic input in the form of heat (Damer and Deamer, 2015; Deamer and Georgiou, 2015; Van Kranendonk *et al.*, 2021).

### 2.2. Lipid biomarkers in hot spring vents and proximal geyser aprons (65–100°C)

#### 2.2.1. Polyether compounds

Our biomarker tour around highly characterized geothermal sites on Earth starts at the spring vent, the most visually striking site in the proximal apron (65–100°C), where fluids or gases reach the surface in the form of geysers, fumaroles, and hot springs, and where water boiling points range from 75°C to 95°C depending on altitude (Dunckel *et al.*, 2009). This is home to thermophiles: organisms that must adapt their cell membranes to resist high temperatures and thermal stress (Fig. 1, bottom half), for which they incorporate a series of structural motifs and chemical bonds that stabilize their heat-resistant plasma membranes (Albers and Driessen, 2007; Boyd *et al.*, 2013). Ether bonds that feature C-O-C linkages, for example, are less readily hydrolysable than ester bonds, and they occur in some bacterial but mostly in archaeal membranes, where glycerol diether lipids such as archaeol, a ubiquitous core lipid among archaea, are common (see Section 4 in the Supplementary Appendix) (Koga and Mori, 2005).

Similarly, long C_30_–C_40_ tetraether lipids, dicarboxylic acids, or diols span the membrane while increasing its rigidity via covalent bonds to eventually reduce proton permeability (Carballeira *et al.*, 1997; Koga, 2012). Polyether membrane-spanning compounds comprise molecules with two alkyl chains that contain two or four ether linkages (C-O-C bonds): DGDs (two ethers) and GDGTs (four ethers). The more ether bonds, the higher the contribution of these lipids toward membrane stabilization on exposure to high vent temperatures (Jain *et al.*, 2014). In addition, some GDGTs contain phytanyl groups with varying degrees of cyclopentane rings that provide additional membrane rigidity (Albers *et al.*, 2000; Huguet *et al.*, 2017).

Non-isoprenoidal GDGTs with a maximum of two rings are associated to some species of soil bacteria. Conversely, isoprenoidal (*i.e.*, containing phytanyl chains), highly ringed structures (named as GDGT-*n* in the case of Archaea, where *n* is the number of rings) are usually recovered from extremophilic archaea (Boyd *et al.*, 2013; Lü *et al.*, 2019) (Table 1). Several studies in the hot springs from the Taupo Volcanic Zone (TVZ) in New Zealand have identified archaeol (a typical archaeal DGD) and various types of GDGTs with contrasting degrees of cyclopentane moieties (Pancost *et al*., 2005; Kaur *et al.*, 2015) attributed to the two archaeal superphyla: Proteoarchaeota and Euryarchaeota. However, besides Archaea, some specific bacterial orders such as Theromdesulfobacteriales but especially Aquificales were inferred in the TVZ by DGDs of similar chain lengths (two alkyl chains: C_17_-C_18_, C_18_-C_18_, and C_18_-C_19_) and by C_18_ monoether compounds such as 1-O-alkylglycerols, which link an alkyl chain and a glycerol through a single ether bond (Pancost *et al.*, 2005).

Saturated DGDs ranging between C_16_ and C_18_ associated to the sulfate reducer *Thermodesulfobacterium commune* were detected in the Octopus Spring (YNP) by Jahnke *et al*. (2001) and by Zeng *et al*. (1992a, 1992b), who, in addition, identified long chain diols (ranging C_18_–C_23_) previously reported in the green non-sulfur *Thermomicrobium* sp. (Pond *et al.*, 1986). Jahnke *et al.* (2001) further expanded studies on thermophilic bacteria in Octopus Spring vents by detecting low molecular weight (LMW) monounsaturated fatty acid (MUFAs) (18:1ω9, 18:1ω11, 20:1ω11, and 20:1ω13), fatty acids with intra-chain cyclopropane moieties (*cy*-C_21_) and the aforementioned monoethers indicative of Aquificales.

#### 2.2.2. Fatty acids and dicarboxylic acids

The average chain length (ACL) of the acyl and alkyl skeletons of membrane fatty acids and ether-containing lipids of thermophiles is systematically higher than in mesophiles (Shen *et al.*, 1970; Vinçon-Laugier *et al.*, 2017). Although this has been proven by comparing membrane fatty acids from Aquificales and Thermodesulfobacteriales (Jahnke *et al.*, 2001; Vinçon-Laugier *et al.*, 2017), as well as different strains of the genus *Bacillus* with differential degrees of thermotolerance (Shen *et al.*, 1970), ACL alone is not an adequate lipid parameter to gauge the presence of thermophiles in a given niche.

This is because fatty acid melting points are not only length-dependent, but also highly compositionally affected, that is, influenced by intramolecular modifications on temperature fluctuations (Shen *et al.*, 1970). Since the compound melting point is so determinant in altering the viscosity and fluidity of the membrane, modifications such as methyl branching, cyclization, and unsaturations must be considered together with ACL. Therefore, as temperature increases, microorganisms reduce the degree of intramolecular membrane lipid modifications in favor of thermostability (Kaur *et al.*, 2015).

Even though these molecular adjustments are not as precise indicators as specific lipid compounds, they do suggest thermophilic life, or its absence. The latter is what Williams *et al*. (2021) concluded after measuring low ACL in the compounds extracted from the vent of an active flowing spring in Hveravellir (Iceland), together with a growing ratio of branched-to-total fatty acids as temperature decreases away from the geyser vent, which related with an increasing gradient of mesophilic microorganisms.

In a more thorough exploration of lipid biomarkers in Iceland (Sánchez-García *et al.*, 2020a), researchers sampled hot springs, mud pots, and fumaroles in Hveragerdi, Krýsuvík, and Námafjall, respectively. The presence of remnants from once active thermophilic organisms was inferred from the detection of dicarboxylic acids ranging from C_6_ to C_15_. The detection of dicarboxylic acids in relation to thermophiles was also reported at El Tatio (Atacama, Chile), the largest geyser field in the southern hemisphere, where silica sinter geyser mounds were explored for their lipid biomarkers content (Sánchez-García *et al*., 2019). The largest presence of thermophile biomarkers was observed in an active geyser mound episodically expulsing liquid water at ∼84°C, representing the hottest type of hydrothermal system of the three that were studied, which, besides the wet geyser system, included a steaming and a dry mound.

In addition, *iso*/*anteiso* (*i*/*a*) fatty acids were detected in all three sinter mounds. *i*/*a* Fatty acids are present in most bacteria (Kaneda, 1991), but they are particularly abundant in sulfate reducing bacteria (SRB) (Table 1), both with and without unsaturation (Taylor and Parkes, 1983; Russell *et al.*, 1997; Jahnke *et al.*, 2014). These acids, and *i*-C_17_ and *i*-C_19_, were also detected in filamentous streamer biomass just below the source pool vent of Octopus Spring (Jahnke *et al.*, 2001), which demands an obligate thermophilic nature from its biological source. Given the high vent temperatures (87°C), it is possible that the *i*/*a* congeners were derived from thermophilic SRB such as *Thermodesulfobacterium commune* or *Thermocrinis ruber* (Jahnke *et al.*, 2001).

### 2.3. Middle and distal geyser aprons and geothermally influenced terrain (≤40–65°C)

Moving away from the hydrothermal spring vent, the temperature decreases and the microbial communities populating the middle (∼40–65°C) and distal (<40°C) aprons are no longer dominated by thermophiles. Instead, the communities populating these sections will be mainly mesophilic and/or thermotolerant (Fig. 1, bottom half). Mesophiles occupy cooler niches around the distal aprons and further geothermally influenced lands. There is a wide range of lipid biomarkers described in diverse hydrothermal areas around the globe that can be related to thermophilic, thermotolerant, and mesophilic biosources with heterogeneous specificity.

#### 2.3.1. Lipid biomarkers of thermophilic Chloroflexi

For instance, long-chain (C_29_–C_31_) mono-, di-, and even triunsaturated alkanes (alkenes) with an obvious dominance of hentriacontatriene (C_31:3_) are characteristically synthesized by green, non-sulfur bacteria such as Chloroflexi (Table 1), a phylum of facultative aerobes largely composed of thermophilic and thermotolerant species. Together with long-chain alkenes, other compounds associated to Chloroflexi are long-chain wax esters (*i.e.*, esters from fatty acids and alkanols) of C_31_–C_40_ in length (van der Meer *et al.*, 2002, 2003).

Shorter wax esters (C_31_–C_37_) are more specific of the *Chloroflexus* genus (van der Meer *et al.*, 2000, 2008), whereas longer ones (C_38_–C_40_) tend to be associated to *Roseiflexus* (van der Meer *et al*., 2002, 2010). The lipid biomarkers for Chloroflexi are not rare within hot spring environments. They have been identified at the Orakei Korako geothermal area in the TVZ (New Zealand) (Pancost *et al*., 2005; Kaur *et al.*, 2015), in Hveravellir (Iceland) (van der Meer *et al.*, 2008) and at El Tatio Middle Basin springs (Megevand *et al.*, 2022).

Still, other studies that have not identified Chloroflexi biomarkers in Iceland have inferred their presence based on the characteristic green/orange gelatinous and filamentous aspect of its mats (Robinson and Eglinton, 1990; Sánchez-García *et al.*, 2020a), which is also the case for studies at El Tatio (Fernandez-Turiel *et al.*, 2005; Dunckel *et al.*, 2009; Sánchez-García *et al.*, 2019). YNP remains as the best characterized hot spring system when it comes to lipid biomarker analyses, and so, various campaigns have identified Chloroflexi-associated lipid signatures entrapped in the sinter samples from the many accessible near-vent areas in the field, such as the Chocolate Pots (Parenteau *et al.*, 2014), Mushroom Springs (Ward *et al.*, 2012), or Octopus Spring (Zeng *et al.*, 1992a, 1992b; Summons et al., 1996; Jahnke *et al.*, 2004).

Further, the abundance of these thermophilic and thermotolerant microorganisms in hot spring environments will positively correlate with an abundance of light-harvesting pigments in the membrane. Obligate thermophiles such as the green non-sulfur *Chloroflexus* genus display chlorosomes (membrane-bound and pigment-rich antenna complexes) with abundant bacteriochlorophyll *c*, but also β- and γ-carotenes (Frese *et al.*, 1997) that are depicted, among others, in Section 5 in the Supplementary Appendix. Species such as *Roseiflexus castenholzii*, on the other hand, express pigments such as bacteriochlorophyll *a* (not *c*) and γ-carotenes, but no chlorosomes or β-carotenes (Hanada *et al.*, 2002).

In other species of thermophilic green sulfur bacteria from the TVZ in New Zealand (*e.g.*, *Chlorobium tepidum*), however, chlorobactene becomes a more abundant and hence relevant pigment (Wahlund *et al.*, 1991). Moreover, a study focused on the membrane carotenoid distribution of the thermophilic non-sulfur bacterium *Rhodothermus marinus* (harvested from submarine hydrothermal systems in Iceland) detected carotenoid glycosides, which are more complex carotenoid molecules bound to terminal, cyclic monosaccharides (Lutnaes *et al.*, 2004).

#### 2.3.2. Lipid biomarkers of thermotolerant Cyanobacteria

Other lipid compounds are rather more related to photosynthetic biosources. This is the case for the linear isoprenoids pristane and phytane (Table 1). Pristane may derive from phytoplanktonic and plant tocopherol (Goossens *et al.*, 1984), and phytane can be derived from archaeol (Brocks and Summons, 2003). Nevertheless, the prime source for both species is chlorophyll *a* from oxygenic phototrophs (Didyk *et al.*, 1978) and bacteriochlorophyll *a* and *b* from phototrophic bacteria such as purple bacteria or green sulfur and non-sulfur bacteria (Peters *et al.*, 2005).

These isoprenoids have been detected in different hydrothermal systems around the globe. In the iron-rich Chocolate Pots (YNP), they were detected in four phototrophic mats whose composition was dominated by either filamentous Cyanobacteria (*Oscillatoria* and *Pseudoanabaena* sp.) or symbiotic associations of coccoid Cyanobacteria and filamentous green non-sulfur bacteria (Chloroflexi) (Parenteau *et al.*, 2014). Similarly, at El Tatio, their presence in silica sinter mounds was related to oxygenic (Cyanobacteria) and anoxygenic (Chloroflexi) photosynthetic microorganisms that were detected by DNA sequencing and/or immunoassays (Sánchez-García *et al.*, 2019). In contrast, the detection of only phytane, together with biphytane and the C_25_ and C_30_ isoprenoid hydrocarbons in streamer biofilm communities from the outflow channels of three hot springs in the Lower Geyser Basin (YNP) was rather considered to originate from archaeol (Schubotz *et al.*, 2013).

Temperature can also help constrain the biological source of phytane, as the upper limit of chlorophyll is 73°C (Ward *et al*., 1998, 2012). Thus, above that temperature, archaea become the most likely source of microbial life for phytane, as photosynthetic life is seriously hampered in those conditions (Tornabene *et al.*, 1979; Peters *et al.*, 2005). This grants temperature a fundamental role in the distribution of phototrophs. Accordingly, this agrees with some of the detected lipidic biomarkers that are associated to Cyanobacteria in the mid and distal aprons of hydrothermal systems. For instance, short-chain alkanes (C_15_–C_20_) with one or two methyl ramifications are common in cyanobacterial membranes (Shiea *et al.*, 1990; Kenig *et al*., 1995; Coates *et al.*, 2014; Hoshino and George, 2015). These are addressed as monomethyl alkanes (MMAs) and dimethyl alkanes (DMAs). In particular, the biosynthetic pathway of the 7Me-C_17_ MMA (methylheptadecane) seems to be unique to the cyanobacterial clade (Coates *et al.*, 2014).

Other common lipidic compounds in Cyanobacteria include short-chain alkenes with one or two sets of double bonds such as heptadecene (C_17:1_), heptadecadiene (C_17:2,_), nonadecene (C_19:1_), and nonadecadiene (C_19:2_) (Coates *et al.*, 2014); or fatty acids with one or multiple unsaturations such as 16:1ω7, 18:1ω7, 18:1ω9, 18:2ω6, 18:3ω3, and 18:3ω6 (Ahlgren *et al.*, 1992; Cohen *et al.*, 1995; Dembitsky *et al.*, 2001; Coates *et al*., 2014). These lipid biomarkers are often described at temperate locations within hot springs from El Tatio, Iceland, New Zealand, and Yellowstone. For instance, several MMAs (Me-C_15_, Me-C_17_, and Me-C_18_) along with octadecene (C_18:1_), MUFAs 16:1ω7 and 18:1ω9, or PUFA 18:2ω6 were detected in silica sinter deposits from El Tatio (Megevand *et al.*, 2022). Their cyanobacterial association was supported by complementary analysis of 16S ribosomal RNA (rRNA) sequencing and the immunodetection of Cyanobacteria (Sánchez-García *et al.*, 2019).

In hydrothermal Iceland, however, samples of microbial mats from the distal aprons of sulfidic hot springs yielded the *n*-alkane heptadecane (C_17_), MMAs from Me-C_17_ to Me-C_19_, and the MUFA and PUFA 16:1ω7 and 18:2ω6. These markers, combined with a stable carbon isotopic signature compatible with the fixation of inorganic carbon through the Calvin cycle pathway (van der Meer *et al.*, 2008), narrowed the biosource to Cyanobacteria (Sánchez-García *et al.*, 2020a). In one of the studies, the reported cyanobacterial lipid biomarkers were found together with diploptene (Sánchez-García *et al.*, 2020a), a membrane-spanning bacterial hopanoid also abundant in Cyanobacteria (Bird *et al.*, 1971; De Rosa *et al.*, 1971; Sakata *et al.*, 1997). Nonetheless, diploptene has low (mostly) prokaryotic specificity (Rohmer *et al.*, 1984) if not accompanied by other photoautotrophic markers nor the conditions that support their growth.

Clearly, there is a plethora of lipid biomarkers consistent across different hot springs that can be traced all the way to the Cyanobacteria phylum (Table 1). What will change across environments is the species, yet such level of taxonomic accuracy requires other assays such as 16S rRNA sequencing. According to Robinson and Eglinton (1990), *Mastigocladus laminosus* is the predominant cyanobacterial species in Icelandic hot springs and the one with highest temperature tolerance (Castenholz, 1969). Interestingly, none of the species sequenced from the sinter samples collected at El Tatio coincided with *M. laminosus*. In contrast, other thermophilic relatives of Cyanobacteria (*Fischerella* sp.) were recently found at a temperature that approaches the upper limit for optimal chlorophyll functioning (73°C) in a hot spring from El Tatio Middle Basin (Megevand *et al.*, 2022). The *Fischerella* genus was identified by the consistent detection of its DNA sequences and due to a characteristic lipid biomarker profile with relative abundance of 4, 5, and 6Me-C_17_, which are lipid congeners ascribed to *Fischerella* sp. (Coates *et al.*, 2014).

YNP is the terrestrial hot spring environment with the most lipid-based microbial characterizations, especially at Octopus Spring. In this particular site, *normal*, MMAs, and DMAs such as C_17_, 7Me-C_17_, and 7,11DiMe-C_17_, along with other MMAs, remain as recurrent examples of lipid biomarkers for Cyanobacteria (Shiea *et al*., 1990; Summons *et al*., 1996; van der Meer *et al*., 2000; Jahnke *et al*., 2004). At the Octopus Spring site but also in the Fountain Paint Pots (YNP), the presence of *Phormidium luridum* was deduced by the detection of MMAs and DMAs convoyed by specific bacteriohopanepolyols (BHPs), which are pentacyclic C_30_-C_32_ hopane-based molecules that can be synthesized by Cyanobacteria (Sáenz *et al*., 2012; Matys *et al.*, 2019). More specifically, the cellular membranes of *P. luridum* were rich in 7/8Me-C_17_ and 7,11DiMe-C_17_, as well as in bishomohopanol and its methylated congener, 2β-methylbishomohopanol (both being BHP derivatives) (Summons *et al.*, 1996; Jahnke *et al*., 2004). Interestingly, *P. luridum* forms cyanobacterial mats of high paleobiological interest due to their ability to construct very distinct, conical-shaped columnar stromatolites (Jahnke *et al.*, 2004).

In other hydrothermal systems within YNP such as the Mushroom Spring or the iron-rich Chocolate Pots, the identification of persistent cyanobacterial lipid biomarkers was associated to the presence of the Cyanobacteria *Synechococcus lividus* (Summons *et al*., 1996; Parenteau *et al.*, 2014), which was supported by 16S rRNA sequence analysis (Becraft *et al.*, 2011). Interestingly, *S. lividus* is known to coexist with bacteria from the Chloroflexi phylum in manifold mats across most YNP alkaline deposits, which makes *S. lividus* one of the most common species of Cyanobacteria in the national park (Shiea *et al*., 1990; Jahnke *et al.*, 2004; Becraft *et al*., 2011).

Beyond YNP, studies in other systems such as hydrothermal Iceland have detected diploptene in microbial mats of presumably *P. luridum* (Summons *et al.*, 1996) or other unidentified Cyanobacteria (Sánchez-García *et al.*, 2020a), in addition to the aforementioned lipid markers in Octopus Spring and Fountain Paint Pots. Interestingly, within the TVZ in New Zealand, longer chain MMAs (C_20_–C_22_) served as biomarkers for the Cyanobacteria *Oscillatoria amphigranulata* (Jahnke *et al.*, 2004), as opposed to the common C_16_–C_18_ MMAs found in the membranes of most other relatives.

#### 2.3.3. Lipid biomarkers in symbiotic formations of Chloroflexi and Cyanobacteria

Within hydrothermal systems, the Chloroflexi and Cyanobacteria phyla habitually coexist forming symbiotic microbial mats, where the former community inhabits the upper layers of the biofilm and protects the Cyanobacteria against UV radiation and high sulfide concentrations (Jørgensen and Nelson, 1988; Skirnisdottir *et al.*, 2000). This phenomenon is continuously reported by lipid biomarker profiles, including molecular features diagnostic of both Cyanobacteria (C_17_ alkanes, C_17:1_ alkenes, C_17_ MMAs/DMAs, homohopanol, bishomohopanol, and methylated congeners of the BHP family) and Chloroflexi (long-chain polyunsaturated alkanes along with C_31_-C_40_ wax esters) such as those described in hydrothermal microbial mats in Iceland (van der Meer *et al.*, 2008), YNP (Shiea *et al.*, 1990; Zeng *et al.*, 1992a, 1992b; Jahnke *et al*., 2004; Parenteau *et al*., 2014), or El Tatio geyser field (Megevand *et al.*, 2022).

Moreover, such a remarkable symbiosis can be further investigated via compound-specific isotopic analysis of the individual lipid compounds synthesized by Cyanobacteria and Chloroflexi. Different metabolic pathways employed by microorganisms will have a particular fractionation signature of carbon that can be used to deepen our understanding about how they interact with their particular extreme environment. Table 1 summarizes these fractionation biosignatures for relevant lipids in hydrothermal settings.

Cyanobacteria fix inorganic carbon from atmospheric CO_2_ through the Calvin-Benson cycle, where an enzymatic preference toward the energetically favorable ^12^C from CO_2_ relative to its ^13^C heavier isotope yields compounds that are depleted in ^13^C relative to CO_2_ (−8‰) by 22–30‰ (Sakata *et al.*, 1997). In contrast, bacteria from the Chloroflexi phylum fix inorganic carbon via the 3-hydroxypropionate pathway (Herter *et al.*, 2002) when growing autotrophically, which imparts unusually heavy δ^13^C signatures to its synthesized lipids (−14‰ to −15‰) (van der Meer *et al.*, 2003).

However, a phenomenon of cross-feeding has been described for thermophilic, green non-sulfur bacteria such as *Chloroflexus aurantiacus* or *R. castenholzii* by acquiring small organics produced by cyanobacterial photosynthesis; implying that they are also capable of growing photoheterotrophically (van der Meer *et al.*, 2010). This metabolic routine has been recognized in Cyanobacteria–Chloroflexi hot spring symbiotic mats, where despite the enriched, bulk δ^13^C biomass values from *C. aurantiacus* pointing toward photoautotrophic metabolisms, the compound-specific δ^13^C signature of glucose and other lipids was as depleted as one would expect from the cyanobacterial Calvin cycle (van der Meer *et al.*, 2003). These isotopic signatures have been described in YNP where Chloroflexi co-inhabited sulfidic hot springs along with the Cyanobacteria *P. luridum* (Jahnke *et al.*, 2004; Zhang *et al.*, 2004) and *S. lividus* (van der Meer *et al*., 2000, 2003, 2010), but also in hydrothermal Iceland with *M. laminosus* (van der Meer *et al.*, 2008); or undetermined (Sánchez-García *et al.*, 2020a).

Sánchez-García and colleagues have also observed this symbiosis in mid-temperature biofilms growing in a hydrothermal spring from the Middle Basin at El Tatio (Megevand *et al.*, 2022). Other remote and hence more inaccessible extreme hot spring environments (Central Australia, Devon Island in Canada, Svalbard territory, or the Kamchatka peninsula) (Preston *et al.*, 2012) are still lacking a more extensive search for lipid biomarkers and deeper analyses of their stable isotope chemistry from which the field would highly benefit.

#### 2.3.4. Ubiquitous lipids: isoprenoids, cyclopropane fatty acids, hopanoids, and pigments

Molecular analysis of lipid by GC-MS often yields compounds that are ubiquitous not only in Cyanobacteria, but also across multiple archaeal and bacterial orders. Examples of these commonalities are saturated or unsaturated fatty acids with 16 or 18 carbons, isoprenoid alkanes pristane and phytane, phytadienes (products of methanolysis of chlorophyll α or anaerobic degradation of phytol), and bacterial diether components that aid against thermal stress (Zeng *et al*., 1992a, 1992b; Grossi *et al.*, 1998).

Cyclopropane fatty acids (*cy*-FAs), a subgroup of fatty acids containing a cyclopropane ring that reduces the fluidity of the cellular plasma membrane, are also widespread across numerous species of bacteria. *cy-*FAs, especially those with 17 and 19 carbons (*cy*-C_17_ and *cy-*C_19_, respectively), have been identified at very low levels in all of the geothermal settings presented here (Jahnke *et al*., 2001; Pancost *et al.*, 2005; Sánchez-García *et al*., 2019; Sánchez-García *et al*., 2020a). Even though *cy-*C_19_ has been allocated as a marker of purple sulfur bacteria (Grimalt *et al.*, 1992; Thiemann and Imhoff, 1996), or the *cy*-C_17_ acid as a marker of green sulfur bacteria (Kenyon and Gray, 1974), both have also been non-specifically designated to Gram-negative bacteria (Wilkinson and Ratledge, 1988; Lange *et al.*, 2014) or anaerobic bacteria (Vestal and White, 1989; Dong *et al.*, 2014), including SRB (Fang *et al.*, 2006; Li *et al.*, 2011). It will eventually depend on the environment, the accompanying lipid biomarkers, and the stable carbon isotope signature whether these somewhat universal biomarkers can be associated to one biological source or another one.

Larger and more complex lipids such as hopanoids and carotenoids also present a ubiquitous distribution across phototrophs. Diploptene, for example, is a hopanoid assumed to be mainly of cyanobacterial origin due to its abundance in said phylum (De Rosa *et al.*, 1971; Sakata *et al.*, 1997), but also a prevailing lipid in methanotrophic bacteria (Rohmer *et al.*, 1984) or even in ferns (Ageta and Arai, 1983) and mosses (Huang *et al.*, 2010). Nonetheless, accompanying hopanoids may constrain the biosource to a more thermophilic origin (Table 1). For example, the *Synechococcus* genus of Cyanobacteria biosynthesizes two different methyl-bacteriohopanetetrol glycosides besides diploptene. These pentacyclic molecules are methylated, and they contain a hopane skeleton bound to a tetrol (four −OH groups) that is, in turn, linked to a carbohydrate (Llopiz *et al.*, 1996).

Interestingly, a recently discovered candidate species of the Acidobacteria phylum (*Chloracidobacterium thermophilum*)—which is also a thermophilic phototroph found in YNP—displays similar hopanoids (Garcia Costas *et al.*, 2012): diploptene, bacteriohopanetetrol (no methyl group nor carbohydrate bound), and bacteriohopanetetrol cyclitol (a tetrol group linked to a cycloalkane instead of a carbohydrate). Regarding relevant diagenetic products, an expected geological alteration of the aforementioned hopanoids is the loss of the carbohydrate/cycloalkane and the loss of the OH groups in the tetrol, leaving homohopane congeners bound to short, saturated alkyl chains (Eigenbrode, 2011), which are molecular fossils to account for in silica sinter deposits.

Light-harvesting pigments such as carotenoids are widely distributed across photosynthetic organisms in all three domains of life, which is why interpreting their biosource allocation is highly dependent on surrounding environmental conditions and accompanying biomarkers. Even though the carotenoids of Cyanobacteria are similar to those of higher plants, the presence of β-carotene and zeaxanthin (β-carotene with two OH groups) (Section 5 in the Supplementary Appendix), echinenone (β-carotene with a ketone C=O group), and myxoxanthophyll (carotenoid glycoside) are appropriate pigment biomarkers of Cyanobacteria (Fecteau *et al.*, 2021; Hirschberg and Chamovitz, 1994). Interestingly, the carotenoid distribution varies depending on the oxygen availability in the system, where β-carotene becomes a predominant pigment in anaerobic conditions relative to the abundance of the other light-harvesting congeners (Hirschberg and Chamovitz, 1994). Some of these compounds are summarized in Table 1.

Generally, the ubiquity of lipids such as isoprenoids, hopanoids, and pigments poses a problem at the time of biosource allocation, which is why lipid biomarker analysis is benefited by other complementary/auxiliary measurements (*e.g.*, a compound-specific isotopic analysis). A suitable case study is farnesol, the ester-bound alcohol side chain of bacteriochlorophyll *c*, *d*, *e* (Scheer, 1991), and *g* (Airs *et al.*, 2001), which are light-harvesting pigments more or less specific of green sulfur (*c*, *d*, and *e*) (Caple *et al.*, 1978), non-sulfur (*c* and *d*) (Gloe and Risch, 1978), or purple sulfur bacteria. Thus, relatively high values of stable carbon isotope fractionation in farnesol (δ^13^C values of ca. −12‰ vs. ca. −22‰) can be attributed to green sulfur bacteria fixing CO_2_ via the reductive tricarboxylic acid pathway (van der Meer *et al.*, 1998; Manske *et al.*, 2005) or to purple sulfur bacteria doing so through the Calvin cycle (Bühring *et al.*, 2009).

### 2.4. Concluding remarks and relevance for Mars exploration

In conclusion, the characteristic lipid biomarkers of hot spring settings have been summarized in Table 1. These would likely include: (1) dicarboxylic acids and polyether compounds such as archaeol and other GDGTs representing membrane adaptations designed to withstand the temperature extremes in the hottest regions (*i.e.*, spring vents, 75–100°C) dominated by thermophilic archaea and bacteria; (2) compounds closely related to thermophilic photosynthetic bacteria, such as Aquificales (18:1, 20:1, and *cy*-C_21_ fatty acids), *Chloroflexus*-like green non-sulfur (long-chain polyunsaturated alkanes and C_31_-C_40_ wax esters), and green (*cy*-C_17_) or purple (*cy*-C_19_, farnesol) sulfur bacteria in proximal slopes (75–65°C); (3) specific combinations of hopanoids (*e.g.*, diploptene, BHP glycosides, *etc.*) and carotenoids (β- and γ-carotene, chlorobactene, zeaxanthin, echinenone, and carotenoid glycosides) common in thermophilic phototrophs; and (4) compounds associated to thermotolerant photosynthetic bacteria (*e.g.*, the esterifying alcohol of chlorophyll—phytol—plus bacteriochlorophylls or derivatives) such as Cyanobacteria (*n*-C_17_, C_17:1,_ C_19:1,_ Me-C_17_, and DiMe-C_17_, as well as 16:1ω7, 18:1ω7, 18:1ω9, and 18:2ω6 fatty acids) or *Roseiflexus*-like green nonsulfur bacteria (C_37_-C_40_ wax esters) in distal aprons (<40°C) plus compounds diagnostic of both thermotolerant (*e.g.*, Cyanobacteria) and thermophilic (*e.g.*, Chloroflexi) photosynthetic bacteria at intermediate temperatures typical of mid-apron pools (40–65°C). Prominently, biomarkers of gram-positive bacteria (*i/a* fatty acids), sulfate-reducing bacteria (10-Me16:0, *i/a*-C_15_, and *i/a*-C_17_ fatty acids), or Actinomycetales (10-Me18:0, *i/a*-C_15_ and *i/a*-C_17_ fatty acids) can be ubiquitous.

Attempts at identifying a “typical” lipid biomarker for hydrothermal environments are hindered by the ubiquity of these compounds around other environments. Still, the molecular complexity required for membrane thermal stabilization proposes a totality of features in alkyl chains that may render specificity for thermophilic microorganisms. Although some compounds alone are suitable to infer archaeal or bacterial inputs, the presence of diether compounds, highly ringed tetraether chains, phytanyl patterns, and dicarboxylic acids (Fig. 1) can be proposed as lipid biomarkers for high temperature settings, among which hydrothermal systems are common.

Beyond specific lipid molecules, what is most relevant in the search for life on Mars is the detection of molecular patterns that imply molecular complexity. In this context, the widespread property of high level non-random branching observed in the phytanyl and di-/tetraether chains of many thermophiles could be considered as an agnostic feature of life in hot spring environments. Yet, billions of years of UV exposure, cosmic radiation, and crystallization processes are likely enough to have caused molecular fragmentation and loss of complexity in the aforementioned molecular patterns. This type of degradation processes could be related to the presence of simple alkyl chains (*i.e.*, C_10_–C_12_ alkanes) of unknown origin on present martian hydrothermal vestiges (Freissinet *et al.*, 2019).

On Earth, abiotic Fischer-Tropsch reactions in magma chambers and aqueous hydrothermal systems can generate hydrocarbons of chain lengths between 16 and 29 carbon atoms (Holm and Charlou, 2001). On Mars, abiotic *n*-alkanes may be similar to any putative biosynthesized congeners (McCollom and Seewald, 2007) that have been subjected to persistent alteration processes. In either case, the amorphous silica discovered by the *Spirit* rover in Gusev Crater could hold lipid fragments whose origin would be, in principle, impossible to discern. To assess biogenicity, future payload designs should be capable of identifying molecular peculiarities (*e.g.*, preference for odd or even carbon numbers in the alkyl chain, specific carbon chain lengths, or compound-specific isotopic composition with alternating δ^13^C values between even- and odd-numbered carbons) that are improbable to originate abiotically (Georgiou and Deamer, 2014).

## 3. Hypersaline Environments

The very driest locations on Mars and on Earth have two factors in common: The past presence of water and a current wealth of evaporitic deposits and precipitated salts that denote preceding intervals of high salinity (Rapin *et al.*, 2019). The following chapters will recapitulate significant findings in the search for lipid biomarkers following a typical geochemical and environmental progression from aqueous environments with high concentrations of salt (aqueous hypersaline environments) to dry, hypersaline basins near water bodies that form as a result of high evaporative rates, which may eventually become hyperarid deserts (Fig. 2) where water activity is noticeably low. As such, terrestrial hyperarid locations tend to be accompanied by hypersaline conditions, and high salinity results in extremely hostile conditions. If life exists in environments of this sort, and it does, it must sustain critical osmotic imbalances and tolerate toxic ion concentrations that can well be lethal (Flowers *et al.*, 2015). Then again, life keeps pushing the edge of habitability toward the apparently uninhabitable, and in this case, that life is represented by halophiles.

### 3.1. Aqueous hypersaline environments

#### 3.1.1. Squalene, gammacerane, and polyether compounds common in hypersaline systems

In general, the unforgiving conditions rendered by elevated salinities filter the number of microorganisms that are able to withstand such taxing osmotic concentrations, and thus, many of the sediments deposited under a hypersaline water column of any given depth are likely to accommodate lipid biomarkers derived from halophilic archaea. These will be enriched in ^13^C by up to 7‰ relative to biomarkers of presumed phytoplanktonic origin (Grice *et al.*, 1998), which, in turn, imparts haloarchaeal isoprenoidal lipids (phytane and C_21_–C_25_ regular isoprenoids) a distinguishable fingerprint when interrogating for the presence of prokaryotic organisms within the sediment.

Squalene is another acyclic isoprenoid (Section 4 in the Supplementary Appendix) whose overall abundance is elevated in the neutral lipid fractions of many archaea in environmental samples from saline lakes (ten Haven *et al.*, 1988). The haloarchaea *Halobacterium cutirubrum*, for example, has been found to contain a noticeably high concentration of squalenes in its total lipid extract. For this reason, and despite its occurrence in most organisms, the predominance of squalene within archaeal membranes (Tornabene, 1978; Grice *et al.*, 1998) allows for its consideration as an appropriate lipid biomarker for halophiles (Table 2) in hypersaline depositional environments (ten Haven *et al.*, 1988).

Also, the C_30_ pentacyclic triterpenoid gammacerane (Section 6 in the Supplementary Appendix) is a biomarker generally associated to a marked water column stratification (Sinninghe Damsté *et al.*, 1995), which are conditions often observed in hypersaline settings (Leeuw and Damsté, 1990; Sinninghe Damsté *et al.*, 1995). Its precursor, tetrahymanol (Section 6 in the Supplementary Appendix), has multiple biosources, with bacterivorous ciliates being the most common one (Harvey and Mcmanus, 1991).

Nonetheless, tetrahymanol has also been isolated from the ubiquitous, phototrophic purple non-sulfur bacterium *Rhodopseudomonas palustris* (Kleemann *et al.*, 1990), from a fern (Zander *et al.*, 1969), or even from a fungus (Kemp *et al.*, 1984). On diagenesis, tetrahymanol loses its hydroxyl group and becomes gammacerane, which, according to a study by Summons *et al*. (1988), can be effectively preserved for up to 850 million years. Studies, therefore, allocate gammacerane not only as a paleosalinity indicator for a given setting, but also as a biomarker for past protozoan input and an element to gauge water column stratification (Summons *et al*., 1988; Sinninghe Damsté *et al.*, 1995).

Like thermophiles, halophilic microorganisms have retained a series of adaptations concerned with their lipid membranes that permits not only their survival, but also their growth in hypersaline settings. An example would be the high concentrations of polyether membrane lipids previously described in high-temperature environments. The DGDs and GDGTs, in this case, employ their ether linkages to contribute toward membrane stabilization and reduction of ion permeability to maintain a proper osmotic pressure (Dawson *et al.*, 2012; Cheng et al, 2017).

Along with other lipid biomarkers, some DGDs such as extended archaeol (often abbreviated C_20_–C_25_ due to both of its alkyl chains) are recurrent biomarkers of archaeal inhabitants in hypersaline environments. Extended archaeol has also been identified in a few species of non-halophilic methanogens (Becker *et al.*, 2016), yet, in hypersaline environments it is considered a hallmark of halophilic Euryarchaeota (Table 2), from the clade Haloarchaea (Dawson *et al.*, 2012), and mostly from the orders Halobacteriales, Haloferacales, and Natrialbales (Vandier *et al.*, 2021). As archaeol is globally distributed among Archaea (Koga *et al*., 1993; Koga, 2012; Tourte *et al.*, 2020), the relative abundance of extended archaeol over archaeol may be used as a proxy of Haloarchaea (Vandier *et al.*, 2021).

#### 3.1.2. Salterns, coasts, and gypsum precipitates

The ubiquity of isoprenoids such as archaeol or squalenes in hypersaline systems highlights the adaptability and therefore abundance of Archaea in locations where halotolerant bacteria are thought to occupy moderate microniches (Antón *et al.*, 2000). In spite of the general outcompeting dynamics of the Archaea domain over Bacteria in high salinities, a study by Antón *et al*. (2002) succeeded at isolating five previously unknown strains of enough similarity to propose what is now one of the most halophilic species of bacteria: *Salinibacter ruber*. These chemoorganotrophic, rod-shaped bacteria isolated from saltern ponds of Alicante and Mallorca (Spain) presented red pigmentation suggestive of β-carotene. In total, there are four major clusters of carotenoid biomarkers organized by their prevalent (or absence of) functional groups within the isoprenoid hydrocarbon chain: hydroxycarotenoids, epoxycarotenoids, carotenes, and ketocarotenoids (Rivera *et al.*, 2014).

The HMW of carotenoid biomarkers almost always results in complex fragmentation patterns of varying abundances that have been thoroughly compiled in a review by Rivera *et al*. (2014). The presence of these fragments along with their relative intensities reflected in liquid chromatography-MS spectra allows for the resolution of the original whole molecule with fidelity. In addition to *S. ruber*, other bacterial species capable of tolerating certain levels of salt concentrations (halotolerant) often thrive in niches within hypersaline environments. Coastal lagoons and tidal channels are rich in laminated mat ecosystems that harbor a plethora of lipid inputs allocated to halotolerant species of Cyanobacteria and Archaea (Table 2).

A study by Jahnke *et al*. (2008) in the hypersaline coastal ponds of Guerrero Negro, Baja California (Mexico), focused on a mat dominated by the filamentous Cyanobacteria *Microcoleus chthonoplastes*, as well as accompanying methanogens and alkaliphilic bacteria. Although the presence of the halotolerant cyanobacterium *M. chthonoplastes* was resolved by sequencing a gene that on expression translates into a nitrogenase enzyme (Omoregie *et al.*, 2004), the presence of deeper halophilic archaea was instead attested to haloarchaeal, isoprenoidal lipid biomarkers: crocetane, squalene, dehydrosqualene, archaeol-like membrane lipids with unsaturated isoprenyl moieties (C_20:1,_ C_20:2_, and C_20:3_), and trace amounts of the isoprenoidal GDGTs caldarchaeol (GDGT-0) and crenarchaeol (GDGT-5) (Jahnke *et al.*, 2008).

Other lipid and stable isotope studies in equivalent environments such as the striking red brines of the Camargue coast (France) (Fourçans *et al.*, 2004; Rontani and Volkman, 2005) or the remote shores of Christmas Island (Sachse and Sachs, 2008) described cyanobacterial biomarkers (7-methylheptadecane, pristane or phytane) for halophilic (*Phormidium* sp.) and halotolerant (*Microcoleus* sp.) populations of Cyanobacteria, as well as for SRB (*i*/*a*-C_15_/C_17_ fatty acids) or purple photosynthetic bacteria (*cy*-C_19_).

Besides its vast salterns, Guerrero Negro hosts other hypersaline, non-halite gypsum structures and precipitates (CaSO_4_·2H_2_O) that form subaqueous crusts and granules inhabited by multiple endoevaporitic microbial communities. These structures are depicted in Fig. 2. The lipid and isotopic biosignatures and the phylogeny of these gypsum units were also explored by Jahnke and Des Marais (2019) and Jahnke *et al.* (2014), who resolved a <1 mm superficial mat dominated by the *Euhalothece* cyanobacterial genus. The lipid biomarker examination reflected the large microbial diversity that phylogenetic analyses suggested, including Cyanobacteria.

Results comprise not only chlorophylls and bacteriochlorophylls, but also methylated hopanoid congeners that could belong to Cyanobacteria or anaerobic, purple non-sulfur bacteria depending on the methyl branch position or even the depth at which the gypsum sample was obtained (Jahnke *et al.*, 2014). Indeed, the enriched δ^13^C values of bishomohopanols relative to alkanes and fatty acids (Δ^13^C of 4–7‰) and to acyclic isoprenoids (Δ^13^C of 14‰) within the gypsum conglomerates point toward a population of anoxygenic purple photoautotrophs (Jahnke and Des Marais, 2019). In addition, an array of monomethylated alkanes (C_15_–C_18_) were allocated mostly to halotolerant Cyanobacteria, as well as diverse fatty acid congeners (18:1ω7, 18:2ω7, and 16:1ω7). Other detected lipids were tentatively associated to Alphaproteobacteria (18:1ω9/11/12, 19:1ω6/8, and the *cy*-C_19_ acid), SRB (*i*-C_17_ and 10-Me18:0), or algae (PUFAs such as 18:2ω9) (Jahnke *et al.*, 2014). Most of these lipid biomarkers were also detected in hypersaline carbonate and gypsum precipitation ponds from the Ebro Delta in Spain (de Oteyza *et al.*, 2004) and in analogous gypsum crusts submerged under the hypersaline salterns of Eliat, Israel (Sørensen *et al.*, 2005). Biomarker studies on gypsum crusts, therefore, imply that endoevaporitic microbial communities are bacterial rather than archaeal, which, in turn, hints at a microniche of tolerable osmotic imbalances where halophilic archaea do not thrive.

Although halite- and gypsum-rich microniches have been thoroughly explored, it would be of great benefit for the field to launch campaigns in other evaporite scenarios that combine gypsum crusts, halite-rich lagoons, and a wide range of salinities such as the Andean salt flats of Pajonales and Gorbea, in Chile. These settings with diverse mineralogy (and yet clearly hypersaline) would be of profound interest to acquire a more systemic and broadened perspective regarding their scarcely studied microbiology (Tebes-Cayo *et al.*, 2021) and lipid biomarker distribution.

#### 3.1.3. Lipid biomarkers in the Dead Sea

This review has progressively carried us from ponds, salterns, and shores to the most notorious hypersaline water mass on Earth: the Dead Sea. Between Jordan and Israel, its 304 m of depth makes this landlocked environment the deepest saline lake on the planet, and its salinity of 342 g/kg, which is roughly the equivalent to 10 times as salty as average ocean waters (Pletcher, 2021), makes the Dead Sea a very unwelcoming environment for life.

A study by Stiehl *et al*. (2005) performed molecular and isotopic analysis on pre-isolated cultures of microorganisms known to thrive in the Dead Sea. Three archaeal cultures composed of the genera *Halorubrum*, *Haloferax*, and *Haloarcula* yielded mostly DGDs, specifically archaeol and some of its unsaturated derivatives (Stiehl *et al.*, 2005), in addition to squalene and homologs with distinct degrees of unsaturation. Part of the reason why squalene is a common lipid biomarker for archaea (Tornabene *et al*., 1979; Grice *et al.*, 1998; Stiehl *et al.*, 2005) is because of its positive charge, which is essential within the archaeal membrane to counteract the negative charges from DGDs that would otherwise compromise cell integrity (Stiehl *et al.*, 2005).

In addition, cultures of the only unicellular photosynthetic algae known to inhabit the Dead Sea water column (*Dunaliella parva*) yielded phytol and plenty of saturated fatty acids of diverse lengths (C_10_–C_28_) but with peaks at C_16_ and C_18_. While saturated C_16_ fatty acids are abundant and ubiquitous in most organisms (Harwood and Russell, 1984), PUFAs such as C_18:2_ are more common components of photosynthetic algae, but also found in Cyanobacteria and protists (Ahlgren *et al*., 1992; Dijkman and Kromkamp, 2006). A unique hypersaline environment such as the Dead Sea, however, would filter most microbial life, and so these ubiquitous lipid biomarkers can be easily linked to *D. parva* (Evans and Kates, 1985; Stiehl *et al*., 2005).

As opposed to the pre-isolated cultures utilized by Stiehl et al, (2005), a previous campaign by Oldenburg *et al*. (2000) obtained samples from the Dead Sea floor sediments directly under the water column. Their lipid biomarker analysis revealed many molecules (*n*-alkanes with peaks at C_27_, C_29_, C_31_, and C_33_; *n*-alkanols with a maximum at C_27_ and sterols such as stigmasterol and β-sitosterol) that suggested terrestrial and therefore allochtonous inputs related to higher land plants (Oldenburg *et al.*, 2000) (Table 2). These findings were supported by depleted compound-specific δ^13^C values directly associable to a C3 plant metabolism. Yet, the enriched δ^13^C signature of phytol (−19‰ to −22‰) did not correspond to the highly ^13^C-depleted C3 metabolism of terrestrial plants (from −33‰ to −39‰), and thus, it was traced back to autochthonous photoautotrophs such as the eukaryote *D. parva*. This reflects the utility of stable isotope chemistry as a complementary approach to the broad and sometimes indistinct results derived from lipid analyses. Other molecules such as *i*/*a*-FAs (C_14–18_) and DGDs reflected a contribution from halophilic bacteria and archaea (Kaplan and Friedmann, 1970; Oldenburg *et al.*, 2000).

#### 3.1.4. Lipid biomarkers in hypersaline lakes

Another saline lake that has been studied from the lipid perspective is Mierlei Lake, in Transylvania (Crognale *et al.*, 2013). Out of the 12 fatty acids detected at Mierlei, four were *i*/*a* mid-chain saturated acids associated to Gram-positive communities. The MUFA 16:1ω7, despite being present in a variety of prokaryotic and eukaryotic microorganisms, was related here to coccoid-shaped species of Cyanobacteria known to be halotolerant under saline—but not hypersaline—conditions such as those of Mierlei Lake. The identification of the 10-Me16:0 fatty acid was assigned to halotolerant, sulfate-reducing Actinomycetales (Crognale *et al.*, 2013). Finally, *cy*-C_19_ was related to the presence of halophilic purple sulfur bacteria (Thiemann and Imhoff, 1996). These data are included in Table 2.

Results from Mierlei Lake to an extent, parallel those obtained by Carrizo *et al*. (2022) in a set of high altitude (3340 masl) Andean ponds with differential degrees of salinity. In this case, altitude and UV radiation were expected to impose an additional constraint to life. Despite the lack of visible vegetation, lipid biomarkers such as diploptene, C_17_ alkane, C_17–_C_18_ alkenes, and C_17_ MMAs and DMAs suggested the presence of microorganisms capable of oxygenic phototrophy. The sources of these hydrocarbons were narrowed down to Cyanobacteria (Carrizo *et al.*, 2022) thanks to the co-detection of fatty acids such as 18:1ω7 or 18:1ω9, and isotopic fingerprints largely resembling the Calvin cycle, that is, δ^13^C values from −18.7‰ to −30.9‰ corresponding to hydrocarbons and −14.8‰ to −28.8‰ to fatty acids.

Cyanobacteria in these Andean lakes (supported by phylogenetic assays) were assumed to be halophilic as they shared the same desiccated, hypersaline lacustrine niche with haloarchaeal organisms. Archaea were, in turn, inferred by the detection of the isoprenoidal hydrocarbons squalene, dehydrosqualene and tetrahydrosqualene, as well as archaeol (Carrizo *et al.*, 2022). On the other hand, anoxygenic phototrophs such as purple sulfur bacteria (inferred by farnesol and *cy*-C_19_) were less abundant in conditions of increasing salinity. This applied to SRB families, likely *Desulfobacteraceae*, *Desulfovibrionaceae*, *Desulfobulbaceae* (terminally branched *i*/*a*-C_15_/C_17_, mid-branched 10-Me16:0 and *cy*-C_17_) (Kuever, 2014), or *Desulfohalobiaceae* (10-Me18:0) (Jahnke and Des Marais, 2019). To a large extent, SRB and archaea carry out heterotrophic carbon assimilation pathways and in the case of the Andean salt lakes, it could be based on the intake of cyanobacterial exudates as reflected by their compound-specific δ^13^C fingerprint (Carrizo *et al.*, 2022).

#### 3.1.5. Summary and relevance for Mars exploration

Out of the lipids presented in this section, only gammacerane can be directly associated to the water column, and to stratified and (likely) saline waters (Sinninghe Damsté *et al.*, 1995). Given the numerous eukaryotic and prokaryotic biosources of tetrahymanol (its precursor molecule), gammacerane is unable to provide specific information on the local microbiology. Further, archaeal lipids such as crocetane, squalene, dehydrosqualene, and archaeol are omnipresent in the membranes of Archaea, although not specific for aqueous hypersaline environments. Extended archaeol, however, could be a more specific haloarchaeal marker (Dawson *et al.*, 2012), and thus of hypersaline environments. In general, DGDs seem more abundant than GDGTs in certain hypersaline settings (Jahnke *et al.*, 2008). Regarding halophilic bacteria, specific lipid biomarkers for aqueous hypersaline environments remain unclear.

Although not exclusive to aqueous hypersaline environments, the presence of carotenoids such as β-carotene or isorenieratene is persistent across the membranes of anaerobic green sulfur bacteria inhabiting marine or smaller aqueous saline bodies (Hopmans *et al.*, 2005). The presence of bacterial carotenoids in hypersaline settings suggests the importance that this class of compounds may have had in the context of UV protection on Mars, where current evaporitic basins such as Gale or Jezero have been confirmed as ancient reservoirs of liquid, saline water. However, the destructive effect of a highly oxidizing soil chemistry, together with the cumulative effect of a powerful cosmic radiation and UV incidence practically assures the absence of pigment-like compounds as such, but rather as diverse degradation products (*e.g.*, β-carotane, the unsaturated homologous of β-carotene) (Section 5 in the Supplementary Appendix), or fragments resulting from their alteration (*e.g.*, long-chain linear isoprenoids) and posterior cyclization to individual aromatic rings. This is relevant for martian settings such as the Gale paleolake where aromatic compounds have been detected in 3-billion-year-old mudstones (Eigenbrode *et al.*, 2018).

### 3.2. Dry evaporitic basins

#### 3.2.1. Lipid biomarkers in the Sdom formation at the Dead Sea basin (Israel)

The transition from aqueous hypersaline bodies to increasingly arid environments necessitates an intermediate stop: dry, hypersaline basins (see Fig. 2, salt deserts). These locations resemble martian landing zones such as Gale Crater, a paleolake basin currently surveyed by the *Curiosity* rover where deposits disclose evidence for several brines from extreme evaporative concentration (Rapin *et al.*, 2019; Thomas *et al.*, 2019).

The Sdom formation, a Miocene/Pliocene 2 km-thick evaporite halite sediment (Charrach, 2018) with extremely low carbon content just by the Dead Sea shores, represents a good scenario to explore fossil biomarkers of past aqueous environments. Here, Grice *et al.* (1998) carried out a study focused on stable carbon isotope chemistry of molecules of lipidic nature, hence providing an insight into the lipid biomarkers that can be found in settings of this kind.

Researchers identified in 10 evaporite deposits a hydrocarbon fraction mainly composed of pristane, phytane, C_21_–C_25_ regular isoprenoids, and squalane (the saturated degradation product of squalene). Although pristane and phytane are often associated to photosynthetic organisms, the δ^13^C content of all isoprenoids was enriched in ^13^C by up to 7‰ compared with other biomarkers of presumed phytoplanktonic origin (steranes and hopanes), and thus, their biosource was deemed to be halophilic archaea. The predominance of the C_21_–C_25_ linear isoprenoids was interpreted to be derived from ether-bound membrane lipids of halophilic archaea on release at early stages of diagenesis (Grice *et al.*, 1998).

The lack of DGDs and GDGTs near the dry Sdom Formation could be attributed to low amounts of living bacteria and archaea. The detection of the linear isoprenoid squalane, but not the unsaturated homolog squalene, suggested a taphonomic effect (*i.e.*, loss of double bonds) of diagenesis. The absence of a ubiquitous archaeal marker such as archaeol also supports the presence of molecular remnants rather than extant archaea. Archaeol and other isoprenoidal GDGTs are composed of two phytanyl chains (core lipids) bound to a polar glycerol (polar lipid). On cell death, hydrolysis of the polar groups separates the recalcitrant core lipids from the polar lipids (White *et al.*, 1979; Lengger *et al*., 2013), with the former lipids being the ones that can remain intact over geological timescales (Jenkyns *et al.*, 2012).

Due to the hypersaline nature of the Sdom Formation, the aforementioned phytane and unspecified long-chain isoprenoids (C_21_–C_25_) (Grice *et al.*, 1998) could be modified core lipids of previous archaeol molecules from haloarchaea. Isoprenoidal and cyclic GDGTs such as caldarchaeol are expected to degrade in a similar way: Phytanyl chains are likely to hydrolyze from their polar groups, yielding longer (C_40_) linear isoprenoids such as biphytane diols and diacids (Schouten *et al.*, 2013), but it remains unclear whether biphytanes break into shorter isoprenoidal chains over time. These processes may have significant implications for martian astrobiology, as they imply that current hyperarid, saline soils that were once flooded by hypersaline lakes could similarly preserve mid- and long-chain linear isoprenoids that should be considered as future mission targets.

#### 3.2.2. Lipid biomarkers in the Negev Desert (Israel)

Further south from the Sdom formation in Israel, the salt concentrations are no longer as ferocious as in the Dead Sea. Concentrations decrease, but so does water availability. The most immediately adjacent dryland to the Dead Sea is the Negev Desert, a region with salt-enriched soil and a handful of vegetation patches. A good example on how to examine a desert utilizing lipid proxies was carried out by Ben-David *et al*. (2011), where the authors studied the microbial community of patchy arid and semi-arid zones of the Negev Desert through fatty acid analysis. They used a number of fatty acid ratios and biomarkers to characterize soil microbial community structures, assess their physiological status, and identify stress factors. For instance, the MUFA/branched fatty acid ratio was employed as a proxy to assess the relative abundances of Gram-negative versus Gram-positive bacteria, as the former tend to express more branched acids (Ben-David *et al.*, 2011; Kaur *et al.*, 2005).

Moderately higher amounts of *cy*-C_17_ and *cy*-C_19_ fatty acids were related to anaerobic bacteria (White *et al.*, 1996) and an elevated proportion of *cy*-C_17_ relative to 16:1ω7 was interpreted as indicative of physiological stress likely related to high soil salinity, high radiation, and/or high evaporation rates. In this study, the presence of 16:1ω7 and 18:2ω6 was related to Cyanobacteria and was in accordance with previous findings that have established *Microcoleus vaginatus* as the main cyanobacterial species in the area (Dembitsky *et al.*, 2001).

#### 3.2.3. Lipid biomarkers in the Dalangtan Playa (Tibetan Plateau, NW China)

Investigating the subsurface of terrestrial analogs rather than just scooping their surface regolith may be a more adequate (and potentially successful) strategy to search for remains of a hypothetical past life on Mars, considering the highly oxidizing chemistry and intense radiation on the martian surface. One of these pioneering studies was at the Tibetan Plateau, where a subsurface composed of halite, gypsum-rich, and detrital materials was surveyed down to 680 cm in depth. The lipid biomarker survey was focused on arid subsurfaces at an ancient depositional center called the Dalangtan Playa (Cheng *et al.*, 2017). This 80 km-long and 30 km-wide stretch of salty crusts of halite and gypsum is situated within the Qaidam sedimentary basin at 5000 masl. Cheng *et al*. (2017) found lipid biomarkers of archaea (archaeol and caldarchaeol) and bacteria (MUFA and branched fatty acids, including mid-chain methylated and *i/a* fatty acids) preserved within the Dalangtan evaporites.

Most of the archaeal biomarkers were identified in the halite- and gypsum-rich upper layer, whereas the bacterial markers were relatively more abundant deeper in the subsurface in a detrital unit away from the salt crust (Cheng *et al.*, 2017). Halophilic archaea were associated to archaeol, which was constitutively present in both subsurface units, but not in the surficial halite crust due to the intense UV radiation in altitude, which, in turn, reinforces the argument for subsurface sampling. Caldarchaeol, tentatively associated to the archaeal phyla Methanobacteriota and Thermoproteota (Blaga *et al.*, 2009), was predominant in the high-salinity units from Dalangtan. Unless halophilic lipid biomarkers achieve an excellent degree of preservation, possible diagenetic products of GDGTs such as unbranched long-chain isoprenoids or other unknown congeners should be interesting targets in the subsurface of martian evaporitic basins.

#### 3.2.4. Lipid biomarkers within salt deposits in the New Yorkshire Bouley Mine (United Kingdom)

Cockell *et al*. (2020) conducted a remarkable lipid biomarker survey in a halite-rich 250-million-year-old (Permian) Zechstein sequence located 1.2 km under the surface of North Yorkshire. The lipid extract was dominated by long-chain alkanes, but there was also a low abundance of hopanes and steranes and interestingly, no isoprenoidal lipids (Cockell *et al.*, 2020). Given these results, the authors interpreted that the samples were devoid of fresh biosignatures due to either depositional effects (low biomass present during salt precipitation) or to microbial/physicochemical diagenesis (Cockell *et al.*, 2020). This is supported by the lack of even or odd carbon preference from the detected *n*-alkanes, as well as the lack of alkenes and aromatics. Still, this study demonstrated the suitability of salt crystals within ancient deposits to preserve biomolecules, especially lipids (Farmer and Des Marais, 1999), which supports orienting the exploration of Mars toward halite deposits with instrumentation capable of lipid identification, such as the SAM instrument from *Curiosity* or the MOMA from the *Rosalind Franklin* rover.

### 3.3. Hyperarid environments: the case of the Atacama Desert

#### 3.3.1. Geophysical features and preservational aspects of the Atacama Desert

Arid environments can be categorized employing an Aridity Index (AI) that takes two governing factors into account to quantify desiccation: the mean annual precipitation divided by the mean annual evapotranspiration (Barrow, 1992). If the AI value of arid deserts is <2, hyperaridity is defined by <0.05, and only a minority of deserts on Earth reach these values (Meslier and DiRuggiero, 2019). Whether polar or not, hyperarid deserts are landforms with no flowing groundwater and where fluvial incision and deposition have ceased. Unforgiving ecosystems where a strategic biology is of utmost importance to withstand the many environmental stress factors in play (prolonged desiccation, high evapotranspiration, strong UV influx, oscillating temperatures, oligotrophic conditions, and/or hypersalinity), and yet, due to their striking biological and hydrological paucity, these environments might hold the key to support the preservation of biosignatures to remarkable extents. This is why the driest deserts on Earth have become consensus terrestrial analogs to present Mars (Fairén *et al.*, 2010; Preston *et al.*, 2012), and as such, it is crucial to gather the most common lipid biomarkers that have been found in such subaerial and subterranean systems.

The desolate Andean plains of the Atacama Desert extend over a vast 1000 km-stretch reaching altitudes that exceed the intemperate 6000 masl. This 10–15 million-year-old desert receives a mean annual rainfall of <2 mm, but there are years with no precipitation at all (Bull and Asenjo, 2013). Geomorphological features of the Atacama include hyperarid pavements, hypersaline plateaus, geothermal deposits, playas, alluvial fans, aeolian features, strong UV incidence, oxidizing chemistries, and soil perchlorates like the ones discovered in 2008 by the *Phoenix* lander on Green Valley near the martian North Pole (Navarro González *et al*., 2003; Fairén *et al.*, 2010; Preston *et al.*, 2012). These conditions amalgamate to encourage a series of strategies that allow the long-term preservation of biomolecules in the subsurface of Atacama, such as protective mineral-organics interactions, halite encapsulations within matrices, salt-derived enzymatic inhibition, cellular adaptations, entombment by chemical precipitation, and subsequent xeropreservation following an extraordinarily low water activity (Parro *et al.*, 2011; Fernández-Remolar *et al*., 2013; Wilhelm *et al*., 2017; Sánchez-García *et al*., 2018; Shen, 2020; Sánchez-García *et al.*, 2021). All of this, supplemented with the high preservation potential of lipid compounds, crafts a recipe for optimism.

#### 3.3.2. Lipid biomarkers in the subsurface of Yungay

There are numerous studies that focus on the microbial diversity available in different regions of Atacama, but only a few have made a profound exploration of the lipid biomarker profile in its subsurface. A study by Wilhelm *et al*. (2017) involved a group of US scientists including NASA researchers that focused on one of the most inhospitable and dry cores of the Atacama Desert: the Yungay region, an area that has been dry since the Jurassic (Hartley *et al.*, 2005) and that experiences less than 2 mm of rainfall annually (McKay *et al.*, 2003). Here, core samples as deep as 2.5 m—resembling the ExoMars *Rosalind Franklin* Drill Unit's maximum depth (2.0 m)—covered a ∼90 cm depth upper gypsiferous unit as well as clay-rich units at >90 cm interrupted by a dense, thin halite unit at 140–150 cm acting as an aquiclude (Wilhelm *et al.*, 2017).

The most abundant lipid species in the gypsiferous unit of the Yungay were fatty acids that peaked at C_16_ and C_18_, but also *iso* and *anteiso* congeners with abundance, diversity, and unsaturations that increased with depth toward the clay unit (>90 cm) and away from the gypsiferous surface (Wilhelm *et al.*, 2017). Taking that into consideration plus the fact that these fatty acid species are prone to diagenesis (Brocks and Summons, 2003), their increasing abundances with depth suggest an evolving stage of xeropreservation (*i.e.*, preservation by drying). The Yungay surface and the upper gypsiferous unit, the most exposed layers to precipitation, display a slight even-over-odd predominance of *n*-alkanes. In the clay unit, a weak archaeal signal (isoprenoidal GDGTs) was observed relative to a clear dominance of bacterial branched GDGTs. In contrast, the well-cemented halite unit was rich in archaeol and extended archaeol, suggesting a past period of intense evaporation followed by extreme aridity that kept halophilic archaeal markers almost intact. These are desirable conditions for evaporitic basins on Mars.

Later, Sánchez-García *et al.* (2018) and a team of Spanish scientists from Centro de Astrobiología (CAB) in Madrid carried out a study at the hyperarid and hypersaline conditions homed by Salar Grande. A drill down to the 100 m-mark allowed for the profiling of the lipid biomarker record through first, a halite unit (down to 40 m of depth) and second, a well-defined detrital unit (40–100 m of depth) (Sánchez-García *et al.*, 2018).

The team found a number of *n*-alkanes and fatty acids with an even-over-odd prevalence indicative of microbial activity and identified several mid-chain MMAs associated to Cyanobacteria (Table 2) and branched alkanes with quaternary carbons linked to sulfide oxidizers (Kenig *et al.*, 2003). The higher abundance of branched fatty acids (*i*/*a* pairs C_15_ and C_17_) and MUFAs (16:1ω7, 18:1ω9, and 20:1ω13) compared with their saturated counterparts was interpreted in terms of different preservation strategies of the labile branches and double bonds (Sánchez-García *et al.*, 2018).

The occurrence of all these bacterial markers was mostly within the large 40 m-thick halite unit. The fivefold larger signal of hopanoids over steranes and diasteranes indicated an evident preponderance of prokaryotic versus eukaryotic fingerprint, mostly within the halite unit (Sánchez-García *et al.*, 2018). There were nine types of hopanoids detected (C_27_–C_34_), among which the homohopanes series (C_30_–C_34_) showed a dominance of the S over the R enantiomers, suggestive of early diagenesis by heavy microbial processing (Sánchez-García *et al.*, 2018). In addition to MMAs and MUFAs, Sánchez-García *et al.* pinpointed phytol-derived isoprenoids (*i.e.*, pristane and phytane), related to halotolerant Cyanobacteria in a salt-rich element in combination with antibody-based detections. Also, within the upper and thicker halite unit, Sánchez-García and colleagues detected copious amounts of the isoprenoids crocetane and squalane that were attributed to archaea (Table 2), whether methanogenic and/or methanotrophic (Koga *et al*., 1993; Hinrichs *et al.*, 2000) or halophilic (Stiehl *et al.*, 2005), respectively.

Squalene, the precursor alkene of squalane, has a long (C_30_), linear chain with six ramifications and six unsaturations that makes it prone to diagenetic transformation. Consequently, squalane and a variety of unsaturated derivatives resulting from the cleavage of the double bonds are common diagenetic products of squalene, which is, in turn, abundant in the neutral lipid fractions of many archaea in samples harvested from hypersaline environments (ten Haven *et al.*, 1988).

Interestingly, both studies by Wilhelm *et al*. (2017) and Sánchez-García *et al.* (2018) detected lipid biomarkers from higher plants, which have not been present in either the Yungay region nor Salar Grande for thousands of years. These results highlight the distinguished potential of aridity and salinity factors to preserve traces of past life in such extreme environments for up to 2 Ma (Dembitsky *et al.*, 2001; Wilhelm *et al*., 2017; Azua-Bustos *et al*., 2020).

Within the scientific community working with lipid biomarkers, however, it remains uncertain whether plant inputs in plant-devoid environments may have a preservational bias over other lipids due to length and size (Johnson *et al.*, 2020) or if there are unknown alternative pathways of biosynthesis (or diagenesis) of long-chain lipids that may source from organisms other than higher plants.

Later in 2020, Shen published a paper that discussed subsurface (10–20 cm deep) regolith samples from seven different locations of the North Atacama Desert, including Yungay (Shen, 2020), which is also where a Spanish team from CAB went that same year along with an international group of researchers (Azua-Bustos *et al.*, 2020) with the purpose of sampling a 30–45 cm deep smectite unit (platy phyllosilicates). Via fatty acid analysis, Shen was able to detect in all samples a microbial signal in form of saturated fatty acids with even-over-odd preference. He also attributed the abundance of terminally branched *i*/*a* congeners to the Gram-positive Firmicutes phylum based on previous studies (Dong *et al.*, 2006), whereas the CAB team (Azua-Bustos *et al.*, 2020) assigned *i*/*a* pairs C_15_ and C_17_ found in Yungay to SRB membranes (Taylor and Parkes, 1983; Grimalt *et al*., 1992) and other heterotrophs such as *Thermus*, *Deinococcus*, or *Bacillus* genera. This is summarized in Table 2.

The Spanish team identified pristane, phytane, MMAs, and MUFAs 16:1ω7, 16:1ω9 and 18:1ω9; all related to Cyanobacteria (Shiea *et al*., 1990; Summons *et al*., 1996; Jahnke *et al*., 2004; van der Meer *et al.*, 2008; Parenteau *et al*., 2014; Sánchez-García *et al.*, 2019). The only PUFA that Shen detected in his samples was 18:2ω9, commonly associated to eukaryotic sources (higher plants, protozoa, and/or fungi) (Shi *et al*., 2002; Jenkins *et al.*, 2009).

This aligns with Azua-Busto's findings on exogenous eukaryotic material, likely introduced by aerial transport based on lipid molecules and isotopic analyses that detected stigmastanol and various long chain *n*-alkanes of odd predominance (C_27_, C_29_ and C_31_) compatible with the utilization of the Calvin cycle. Also, Shen identified notable amounts of the Proteobacteria-related (Wang *et al.*, 2012) and alkalinity-associated fatty acid *cy*-C_19_ along a >600 km-long latitudinal transect. This highlighted the consistent presence of halotolerant species (*e.g., Halomonas*) from samples obtained beneath or within permanent halite subsurface units in the hyperarid, Andean coastal salt flats (Shen, 2020).

#### 3.3.3. Lipid proxies for paleoenvironmental studies

In 2021, Sánchez-García *et al*. investigated lipid biomarker profiles on three sedimentary records from the Triassic-Jurassic interface in Atacama to assess recent or fossilized life as old as ∼200 Ma (Sánchez-García *et al*., 2021). This time, in addition to identifying biosources and metabolic traits, a number of geochemical lipid proxies were applied to reconstruct the prevailing environmental conditions over time in the three sedimentary scenarios. For example, the carbon preference index (CPI) of *n*-alkanes was used to assess the maturity of the organic matter. Fresh biomass shows a characteristic predominance of odd-over-even number of carbons that produces CPI values >5, whereas mature samples tend to display CPI values approaching 1 due to the progressive loss of the odd/even preference (Rieley *et al.*, 1991; Hedges and Prahl, 1993). The low CPI values measured in the Atacama deposits by Sánchez-García *et al*. denoted some thermal maturity of the Triassic-Jurassic materials, particularly advanced (*i.e.*, strong effect of diagenesis) in one of the three scenarios that showed a relatively smoothed and flattened molecular distribution of *n*-alkanes.

Other ratios provide an idea of the relative abundance of oxygen in sedimentary environments, such as the relation of pristane over phytane (Pr/Ph), as long as they stem from phytol (Peters *et al*., 2005; Blumenberg *et al.*, 2012). Assuming the chlorophyll side chain phytol as the primary origin of both molecules (Rontani and Volkman, 2003), their relative abundance in the sediment is influenced by bottom-water redox conditions at the time of deposition (Didyk *et al.*, 1978), and its ratio (Pr/Ph) can be used to estimate the relative abundance of oxygen in sedimentary environments (Blumenberg *et al.*, 2012).

In the Triassic-Jurassic deposits of Atacama, redox conditions were assessed as dominantly oxic according to Pr/Ph >1. Also, the relative proportion of even low- over odd high-molecular-weight alkanes (LMW/HMW_alk_, *i.e.*, the sum of C_16_ and C_18_ over that of C_27_, C_29_, and C_31_) (Vonk *et al.*, 2012) was employed as a proxy to assess the relative proportion of prokaryotic versus eukaryotic biomass (Grimalt *et al.*, 1986). Other proxies were used to estimate the relative abundance of remnants from autotrophs and heterotrophs, such as the sum of pristane and phytane over the amount of the C_17_ and C_18_ *n*-alkanes ([Pr + Ph]/[*n*-C_17_ + *n*-C_18_]) (Canfield and Des Marais, 1993), or the ratio of branched over the *normal* heptadecane alkane (br-C_17_/*n*-C_17_) (Chen *et al.*, 2011).

Additional lipid ratios used in this study were more focused on ecology, such as the terrigenous-over-aquatic ratio (TAR) and the *n*-alkane P_aq_ index (Derrien *et al.*, 2017 and references therein), which were applied to evaluate the presence in the three Triassic-Jurassic sedimentary systems of aquatic versus terrigenous plant biomass (Sánchez-García *et al.*, 2021).

#### 3.3.4. Gypsum nodules in the hyperarid core

Just like the aforementioned hypersaline shores and wetlands, the hyperarid Yungay core also harbors endoevaporitic halotolerant communities within gypsum nodules that are adjacent to halite layers. It is worth noting that despite being situated in a hyperarid setting such as the Atacama Desert, the hygroscopic nature of gypsum (a property of attraction and retention of water molecules via absorption or adsorption) will make it one of the last microniches with available remaining water, and thus, favoring endolithic life (Buck and Van Hoesen, 2002; Dong *et al.*, 2007; Farías and Saona Acuña, 2020).

This means that the interior environments of gypsum nodules are not necessarily arid ecosystems; however, their presence in hyperarid regions of Atacama is notable. Hence, a detailed study by Ziolkowski *et al*. (2013) involving fatty acid extraction from Yungay gypsum samples detected a clear predominance of the 16:0, 16:1ω9 and 18:1ω9 congeners. Accompanied by the characterization of their stable carbon isotopic composition, which resulted in partially depleted δ^13^C values (from −30‰ to −31‰), the authors ascribed the lipid biomarkers to an autotrophic community that likely comprised Cyanobacteria (Ziolkowski *et al.*, 2013). The interpretations from Ziolkowski *et al*. are in accordance with another molecular and microscopic examination (Montero-Lobato *et al.*, 2020) that confirms the presence of the endolithic *Chroococcidiopsis* sp. of Cyanobacteria within Atacama gypsum samples as the dominant photoautotroph in the most extreme deserts (Nienow, 2009; Wierzchos *et al.*, 2013). Nonetheless, along with cyanobacterial species, Andean gypsum crusts can also be populated by methylotrophs and heterotrophic phototrophs such as Chloroflexi (Schulze-Makuch *et al.*, 2021).

Indeed, gypsum endoevaporitic communities seem to be homed by Bacteria rather than Archaea, the latter, on the other hand, are more predominant within halite units. Reports of permineralized gypsum formations on terrestrial deserts display living and fossil microscopic evidence of entrapped microorganisms (both eukaryotes and prokaryotes) within fluid inclusions, which, like halite, establishes gypsiferous units as favorable preservation matrices for organics (Benison and Karmanocky, 2014). Taken as a whole, salt crystals are definitely a prime example of a martian refugium for future missions to focus on.

If halophilic microorganisms can be preserved within halite for up to ∼250 Ma (Vreeland *et al.*, 2000), it would be recommendable to append gypsum mineralization and entombment to the list of preservation processes available in hyperarid and hypersaline environments (*e.g.*, halite encapsulation), especially given the extensive gypsum-rich evaporitic sulfates on the surface of Mars (gypsum sand, silt grains, molds, and veins) reported by orbital analyses (Bishop *et al.*, 2004; Gendrin *et al.*, 2005) and high-resolution cameras on rovers (Grotzinger *et al.*, 2005) that offer novel opportunities for planetary exploration.

### 3.4. Concluding remarks for hyperarid and hypersaline environments relevant to Mars

Although it has been proven ambiguous to determine patterns for hyperaridity, saline settings are dominated by halophiles, and more specifically archaea in halite units. Membrane lipids of archaea such as crocetane, squalene, dehydrosqualene, archaeol, caldarchaeol, and other DGDs and GDGTs can be common biomarkers in hyperarid, hypersaline environments (Table 2). However, given the relatively more extremized conditions in the evaporitic settings compared with the somewhat water-protected aqueous hypersaline environments (*i.e.*, higher dryness and higher exposure to UV radiation), it is even more unlikely to find archaeal biomarkers in their intact form in the former environments. Instead, isoprenoidal chains and fragments resulting from their defunctionalization or saturation (White *et al*., 1979; Lengger *et al.*, 2013; Schouten *et al.*, 2013; Xu *et al.*, 2020) are to be more expected. Further, isoprenoid chains may progressively lose methyl ramifications and become saturated alkanes (Cockell *et al.*, 2020).

On Earth, early diagenesis is enhanced by the enzymatic action of microorganisms; however, this can be attenuated by the biological paucity of hyperarid and hypersaline environments combined with the geochemical factors in play that support the preservation of organics in the absence of liquid water: xeropreservation, mineral entombment, salt encapsulation, and a variety of mineral-organics interactions. The latter includes several mechanisms that facilitate the adherence of organics to mineral surfaces, such as chemical sorption, occlusion by biomineralization (similar to mineral entombment but in the microscale), and macromolecular organomineral (colloid) aggregations (Keil and Mayer, 2014).

In contrast, the physicochemical parameters driving diagenesis on Mars are different than on Earth due to the lack of plate tectonics and subduction, as well as the very likely absence of microbial-driven degradation of biomolecules. Still, the billion-year timescales of its salt deposits combined with the elevated levels of ionizing radiation and regolith oxidants demand exceptional preservation conditions in the subsurface if there were to be any functionalized lipids (*e.g.*, carboxylic groups or unsaturations). Accordingly, only simple short- and mid-chain hydrocarbons of unknown origin have been found on the martian subsurface (Eigenbrode *et al.*, 2018). All in all, the mineralogy of this section was merely focused on halite and gypsum, but there are other sets of minerals with high encapsulation potential that hold conditions of acidity and chemical toxicity that will be reviewed in the following chapter.

## 4. Acid and Iron Sulfate-Rich Environments

The repertoire of martian sites analogous to extreme environments on Earth can be further expanded by *Meridiani Planum*, whose distinctive mineralogy and geochemistry allocates it as a category of its own among the different martian environments with astrobiological interest. This plain, located just below the martian equator, hosts hematite-rich soils and outcrops, as well as abundant ferric sulfate minerals such as jarosite that were detected by the miniaturized Mössbauer spectrometer onboard the *Opportunity* rover (Klingelhöfer *et al.*, 2004). The presence of these minerals can be explained by a known sequence of processes that take us to an interesting scenario that has not yet been tackled by this review.

First, since jarosite is a hydroxide sulfate mineral (Kfe^3+^_3_[SO_4_]_2_[OH]_6_), its presence in *Meridiani Planum* provides convincing mineralogical evidence for a past aqueous environment (Klingelhöfer *et al.*, 2004). Geomorphological models of martian hydrologic evolution, indeed, support an event of massive groundwater upwelling in the Northern lowlands triggered by the rise of the *Tharsis* volcanic region (Andrews-Hanna *et al.*, 2007).

Second, the hydrous iron-sulfate chemical nature of jarosite implies that its formation involved a process of oxidation of iron sulfides. Assuming *Meridiani Planum* was covered by a shallow ocean in the past, a scenario of aqueous oxidation of pyrite (FeS_2_) is plausible (Squyres *et al.*, 2004). This takes us to the third step, which is the generation of wet acid-sulfate conditions in the ancient groundwater that flooded *Meridiani Planum*, and that on regional heating and dehydration may have left the sulfate salt deposits that *Opportunity* identified in 2004 (Klingelhöfer *et al.*, 2004; Zolotov and Shock, 2005).

In summary, the Northern lowlands of Mars probably once harbored an acidic body of water full of iron oxides and iron sulfides (Klingelhöfer *et al.*, 2004) that, given its chemistry and low pH, might have been prohibitive for any putative form of life. At this point, it is inevitable to turn our heads toward Río Tinto (Spain), an acid and iron-rich river flowing within one of the largest deposits of metallic sulfides on Earth.

### 4.1. Aqueous iron sulfate-rich environments

#### 4.1.1. Lipid biomarkers in the river basin of the acid Río Tinto (Spain)

Río Tinto flows in the core of the Iberian Pyritic Belt, a 250 km-long and 30–50 km-wide geological entity that encloses a vast amount of metallic sulfide deposits. The river itself is 92 km in length, and besides its high concentrations of heavy metals (*e.g.*, Fe, Cu, Zn, Pb, or As), it has a consistent mean pH of 2.3, which allows the elevated concentrations of ferric iron to remain in solution (Amils *et al.*, 2014), giving the river an odd, dark red coloration. This basin has gained a lot of astrobiological relevance because it serves as a terrestrial analog of current and past *Meridiani Planum*. Current because of the iron oxides and ferric sulfates that can currently be found in both environments, and past because the existing depositional processes of Río Tinto and its terraces propose a potentially similar acidic hydrological activity to the one that might have taken place at *Meridiani Planum* during Noachian Mars (Fernández-Remolar *et al.*, 2005).

Further, Río Tinto is crowded with extremophiles that thrive in extreme conditions of acidity, better known as acidophiles. These peculiar microorganisms are chemolithotrophic, meaning they obtain their energy by oxidizing inorganic compounds (mostly metallic sulfides here) and thus, contributing toward the acidification of the system (Amils *et al.*, 2007). All this thriving life at Río Tinto opens a window to the possibility of past or extant extraterrestrial life in locations such as *Meridiani Planum*. Among the biomolecular studies of life in highly acidic environments, those based on lipid biomarkers are thorough but there are only a handful. Unlike other extremophiles for which there are specific biomarkers (thermophiles and halophiles), no lipid compounds specific to extremophilic organisms in acidic environments (acidophiles) are known.

Instead, lipid biomarkers of microorganisms capable of thriving in environments such as Río Tinto must be identified and often combined with other analyses to achieve a more precise biosource specificity. Nevertheless, a summary of lipid biomarkers identified in acid and iron-sulfate rich environments is provided in Fig. 3 and Table 3. This strategy where different techniques are combined for lipid detection was followed by Sánchez-García *et al*. (2020b) on a drilling mission in Río Tinto in 2020, where a multi-analytical approach that included 16S rRNA sequencing, an antibody-based immunoassay, and a molecular lipid analysis allowed for a profound characterization of the Río Tinto subsurface.

As previously mentioned for hyperarid environments, sampling the subsurface of Mars analogs provides a realistic scenario for future endeavors, and in the case of Río Tinto, it is supported by an extensive study by Fernández-Remolar *et al*. (2008) where complex, subsurface methanogenic, and acidophilic habitats are described. Along a 2 m depth profile conducted in an evaporitic esplanade near the Peña de Hierro mining complex, Sánchez-García *et al*. (2020b) found a series of *n*-alkanes that exhibited a larger proportion of odd long chains over even long chains at depths down to 1 m, but not beyond, indicating an increasing presence of microbial biomass with depth. This finding was consistent with an increasing concentration of the *i*/*a*-C_15_ and the *i*/*a*-C_17_ fatty acids with depth, associated to SRB (Table 3) given the geochemical context (Sánchez-García *et al.*, 2020b).

Most of the identified fatty acids were saturated, yet some unsaturated fatty acids related to cyanobacterial sources (18:1ω9 and 18:2ω6) were also found mostly at depth. Interestingly, despite the absence of Cyanobacteria in acid settings (*i.e.*, at pH <4) (Cirés *et al.*, 2017), RNA sequencing and immunoassays supported their presence (*i.e.*, *Dolichospermum*, formerly *Anabaena* sp. and *Microcystis* sp.) in the Río Tinto subsurface, likely by occupation of microniches of higher pH values (>4–5) within the Tinto basin (Sánchez-García *et al.*, 2020b).

The detection of other monounsaturated congeners (18:1ω8) was assigned to methanotrophs (Costello *et al.*, 2002) (Table 3). Ultimately, dicarboxylic acids indicative of thermophiles (Carballeira *et al.*, 1997) were found to be abundant between 40 and 100 cm of depth (Sánchez-García *et al.*, 2020b).

#### 4.1.2. Lipid biomarkers in the acid stream of St. Oswald's Bay (United Kingdom)

Stepping up on the pH scale, the acidic stream (pH 3.5) of St. Oswald's Bay, in Dorset (United Kingdom), also represents an appropriate mineralogical environment to study the sulfate-rich aqueous conditions of Noachian and Hesperian Mars. Like Río Tinto, this stream is also rich in ferric sulfate waters derived from the aqueous oxidation of pyrite, which has, in turn, led to the precipitation of jarosite and goethite (iron oxyhydroxide) (Tan *et al.*, 2018). In the core samples obtained by the edge of the stream at a depth of 10–15 cm, the abundance of saturated fatty acids with even-over-odd predominance and maxima at C_16_ and C_18_ denoted microbial life at St. Oswald's Bay (Tan *et al.*, 2018).

In particular, the presence of SRB was inferred through the detection of *i*/*a*-C_15_ and *i*/*a*-C_17_ fatty acids and the mid-chain branched 10-Me16:0 congener (Vestal and White, 1989; Boschker and Middelburg, 2002). The *cy*-C_19_ fatty acid was only identified in active microbial mats but not in the core samples, and it was associated to anaerobic bacteria (Tan *et al.*, 2018) like purple sulfur bacteria or SRB (Thiemann and Imhoff, 1996; Fang *et al.*, 2006; Li *et al.*, 2011). Similar to the Río Tinto study, the detection in the acidic stream of St. Oswald's Bay of the MUFA 18:1ω9 was also associated to cyanobacterial lipid profiles, but without support of additional techniques. These findings are summarized in Table 3.

Despite the potential conservative role of organics by iron (Lalonde *et al.*, 2012), the probability of finding intact lipid molecules on iron sulfate streams is low due to the high acidity and oxidizing chemistry, which cause the molecules to degrade and adopt poorly studied fragmentation patterns that require further research. For example, fatty acids defunctionalize (*i.e.*, lose their carboxylic functional group) into saturated hydrocarbons (alkanes) by dehydration and decarboxylation. On Mars, the SAM instrument has already detected short chain *n*-alkanes (C_10_–C_12_) in Gale crater (Freissinet *et al.*, 2019). In addition, longer chain *n*-alkanes (up to C_22_) have been found in a number of meteorites (Sephton *et al.*, 2001), although their origin was attributed to terrestrial contamination. Tan *et al*. (2018) also reported the conversion of the anaerobe-characteristic *cy*-C_19_ into C_18_ MMA on specific conditions of acidity and thermal diagenesis over extended periods of time.

Interestingly, although acidity does contribute toward *cy*-FA defunctionalization (Tan *et al.*, 2018), it may have an opposite effect for at least several strains of *Escherichia coli* subjected to an acid challenge (pH 3) *in vitro*. In a process coined “acid habituation” by Brown *et al.* (1997), it appears that the Gram-negative *E. coli* is capable of converting both MUFAs 16:1ω7 and 18:1ω7 to their cyclopropane derivatives *cy*-C_17_ and *cy*-C_19_, respectively.

Since the environmental stress that comes along with an increased expression of cyclopropane rings has allowed scientists to employ the ratio *cy*-C_17_ to 16:1ω7 (the monoenoic precursor of *cy*-C_17_) to assess the physiological or nutritional conditions of a given bacterial community (Crognale *et al.*, 2013), it would be of interest to assess the utility of such ratio when the stress factor is acidity instead of nutrient scarcity. Nevertheless, C_18_ MMAs not only seem plausible defunctionalization products of cyclopropyl fatty acids (Brown *et al.*, 1997), but they are also recurrent lipids in cyanobacterial mats (Tan *et al.*, 2018). It is, therefore, conceivable that diagenesis can confound the interpretation of any given lipid biomarker analysis, whether in acid conditions or in other extreme environments. This highlights the importance of taphonomic studies of lipid preservation in terrestrial analogs to evaluate the impacts of diagenesis, which would largely expand the scope of future life detection prospects.

#### 4.1.3. Lipid biomarkers in the iron-rich Chocolate Pots of YNP (United States)

We finish our ascent in the acid portion of the pH scale in the Chocolate Pots hydrothermal system, with values that range between 5.5 and 6, and with a mean temperature of 51–54°C; in summary, more benign pH conditions that still allow thermophilic prokaryotic photosynthesis. Accordingly, most of the lipid biomarkers identified in this system by Parenteau *et al*. (2014) align with the presence of Cyanobacteria and Chloroflexi. For instance, the PUFA 18:2 and the MUFA 18:1ω9 are mostly considered of cyanobacterial origin, but they were also detected in the membranes of *C. aurantiacus* (Kenyon and Gray, 1974). The oxygen-dependent desaturation mechanism during the biosynthesis of cyanobacterial acyl chains generates a double bond in the ω9 position, as opposed to most anaerobic chain biosynthetic pathways in other bacteria (Cronan and Rock, 2008).

The presence of Cyanobacteria in the moderately acidic Chocolate Pots was further supported by the detection of methyl- and dimethylheptadecanes (C_17_ MMAs/DMAs), as well as hopanoids such as bishomohopanol (plus methylated congeners) (Parenteau *et al.*, 2014). As previously mentioned, the presence of Cyanobacteria-Chloroflexi mats in the Chocolate Pots is supported by the detection of long-chain wax esters (C_32_ and C_34_) and the polyunsaturated alkene C_31:3_ (Parenteau *et al.*, 2014). Fatty acids with *i*/*a* or 10-methyl ramifications were once again allocated to SRB (Parenteau *et al.*, 2014).

Most of the labile lipid biomarkers found in the Chocolate Pots were detected in fresh microbial mats. Their presence in geological samples may be seriously threatened as they tend to be degraded and mineralized easily via microbial metabolic processes. For instance, the oxygen produced by cyanobacterial aerobic respiration can be an immediate oxidizing agent (Brocks and Summons, 2003). Nonetheless, the Chocolate Pots are rich in ferrihydrite, a metastable mineral phase that is even less ordered and crystallized than goethite, and like silica sinter, quite effective at preserving organic matter.

In fact, Parenteau *et al*. (2014) identified against their expectations a small percentage of intact cyanobacterial and Chloroflexi membrane lipids and fatty acids preserved in the ferrihydrite phase under the mats, and thus, empirically supported the idea that goethite is, indeed, a potentially reliable encapsulation agent for organics. There are three proposed mechanisms of lipid preservation in iron-rich amorphous mineral phases.

First, if the aqueous phase is anoxic and Fe(II)-rich, Fe(II) might sequester cyanobacterial oxygen and oxidize into Fe(III) species that precipitate as ferrihydrite, and thus, decreasing biogenic oxygen levels, the most immediate oxidizing agent (Parenteau *et al.*, 2014).

Second, despite the oxidizing potential of Fe(III), the positively charged iron electrostatically interacts with the negatively charged cell surface and fatty acids, leading to a rapid chelation mechanism that accelerates lipid mineralization (Ferris *et al.*, 1988; Warren and Ferris, 1998).

Third, it has been demonstrated that iron can inhibit cellular autolytic enzymes, and therefore aiding in the survival of lipids (Herbold and Glaser, 1975; Leduc *et al.*, 1982).

#### 4.1.4. Diagenesis in iron sulfate-rich environments

Although diagenetic effects can be problematic, its effects in iron sulfate-rich environments can be minimized in two ways. First, by digging deep to obtain samples shielded from radiation and protected from soils with extreme chemistries, just like the study at Río Tinto (Sánchez-García *et al.*, 2020b). Second, by examining the encapsulation and preservation potential of iron deposits in acidic environments.

Along these lines, the presence of jarosite and goethite in Río Tinto, St. Oswald's Bay, and the Meridiani plains is of interest. Just like amorphous silica, it seems that non-crystalline and poorly arranged goethite (which also dominates the terrace mineralogy of the Tinto basin) is particularly good at promoting the long-term preservation of organic molecules (Fernández-Remolar and Knoll, 2008; Lalonde *et al.*, 2012; Tan *et al.*, 2018; Sánchez-García *et al.*, 2020b). However, some studies suggest that diagenetic processes of crystallization and subsequent transformation into hematite would obliterate any enclosed biomarkers (Preston *et al.*, 2011), just like amorphous silica transitioning into quartz.

These studies also suggest that the abundance of Fe(III) in *Meridiani Planum* would generate highly oxidizing conditions that might oxidize the carbon skeleton of the most recalcitrant lipid compounds (Sumner, 2004; Klein, 2005). Moreover, the deconvolution of data obtained by the spectrometers onboard *Opportunity* resolves the presence of nontronite smectite clays at *Meridiani Planum* [(Fe_1.92_Mg_0.03_)(Al_0.23_Si_3.76_)O_10_(OH)_2_] (Glotch *et al.*, 2006). During diagenesis, ferric clays such as nontronites can release electron acceptors such as Fe(III) and Al(III) with potential to catalyze organic reactions and therefore contribute toward the defunctionalization of not only fatty acids, but also more complex lipid biomarkers (cyclopropyl congeners, polycyclic terpenes, *etc.*) with higher diagnostic potential (Tan and Sephton, 2020).

#### 4.1.5. Summary of acid and iron sulfate-rich environments

All in all, the microbiology of these settings is obviously composed of acidophiles, mostly bacterial and without a clear archaeal dominance. Both bacterial diversity and fatty acid complexity seem to increase with depth, which implies better preservation conditions away from hydrological activity, which is comparable to xeropreservative mechanisms of hyperarid settings. Although lipid biomarkers specific to acidophiles are not readily available, there is a plethora of microorganisms with preference for other geochemical and mineralogical features that are also characteristic of acidic environments (*e.g.*, sulfate or iron abundance) whose biomarkers can be used instead (Table 3).

Samples from highly acidic systems such as Río Tinto or St. Oswald's Bay reflect the presence of SRB in form of *i/a*-C_15_, *i/a*-C_17_ and 10-Me16:0 fatty acids, often accompanied by *cy*-C_19_ related to anaerobic (likely purple sulfur) bacteria. Although sulfate reducers are not exclusive to low pH environments, the sulfate-rich mineralogy of acidic environments almost demands their presence. Interestingly, it seems that despite the high acidity of Río Tinto, St. Oswald's Bay, and the Chocolate Pots, an accompanying iron-rich mineralogy can support the preservation of lipid biomarkers. However, a thorough exploration of their diagenetic products and fragmentation patterns with depth remains essential.

## 5. Polyextreme Environments

In this last section, we put lipid biomarker analysis to the test by examining what we consider to be the most challenging combination of constraints available on Earth, or the *true* extreme conditions. These places are addressed as polyextreme environments because they combine conditions of aridity, elevated salinity and acidity that are not usually found together, and on occasions they also include hydrothermal activity. Polyextreme environments (depicted in Fig. 3) are ideal settings to interrogate the limits of life and to address the taphonomy of lipid biomarkers.

### 5.1. The acid and saline lakes of Western Australia

Four naturally occurring acidic, salt lakes in Norseman, Western Australia provide a first insight into the lipid biomarker composition of polyextreme settings (Table 3). These lakes are adjacent to mudflats rich in smectites and phyllosilicates, providing interesting mineral matrices for preservation of organics alongside other sulfate minerals (Johnson *et al.*, 2020).

Most of the lipid biomarkers identified by GC-MS here seem to belong to higher terrestrial plants (*e.g.*, *n*-alkanes, *n-*alkanols, and *n*-alkanoic acids of chains >C_20_) (Eglinton and Hamilton, 1967) or C_29_ sterols (Volkman, 1986), supported by an absence of PUFAs, largely indicative of algae (Volkman *et al.*, 1998). Besides the ubiquitous C_16_ and C_18_ fatty acids, not many more microbial inputs were detected, perhaps due to the eclipsing predominance of plant lipids, which given the low pH of the lakes (1.4–3.7), they are likely derived from dead plant organisms (Johnson *et al.*, 2020). More complementary studies would be desirable to further investigate the scarce presence of microbial biomarkers in the acidic Australian lakes. On the other hand, lipid studies at Río Tinto and St. Oswald's Bay do reveal a complex microbial diversity despite their high acidity (Amils *et al.*, 2007; Tan *et al*., 2018; Sánchez-García *et al.*, 2020b).

This suggests that the conditions of these lakes (where salinity is an additional factor to consider) might have a previously unacknowledged preservational bias for larger plant lipids rather than for shorter microbial fatty acids. That is, assuming there are indeed numerous (detectable) microbial inputs in the acidic Australian lakes. If biases to preserve larger/heavier molecules were true, and since prokaryotes are more astrobiologically relevant than eukaryotes, larger and more complex microbial lipids such as GDGTs but especially the more recalcitrant hopanes would then become priority targets in the search for extraterrestrial biomarkers enclosed in mineral matrices (Johnson *et al.*, 2020).

### 5.2. The Dallol hydrothermal dome (Ethiopia)

Whether there is a predominance or a minority of microbial inputs, there is always a degree of microbial diversity thriving among the most varied and extreme conditions on our lithosphere. This raises a fascinating question: How extreme do terrestrial environments have to be to truly interrogate the limits of life? To tackle a matter of such magnitude, the scope of this review must land on the Danakil Depression, in Northeast Ethiopia, a depression situated 120 m below sea level where an arm of the Red Sea was isolated due to tectonic uplifting, causing an event of massive evaporation and subsequent halite deposition. Within Danakil, the Dallol volcanic dome rises surrounded by a hypersaline and acidic hot spring area.

The weather in the Dallol hydrothermal system is categorized as a desert climate where geysers discharge not only extremely hot fluids (from 90°C to 108°C), but also violently acidic (where pH values go as low as −1.7) (Kotopoulou *et al.*, 2019). These fluids are rich in iron, and due to the mixing with Red Sea waters, they are also halite supersaturated upon discharge. Further, the hyperarid climate of Dallol promotes quick evaporation rates and the subsequent formation of large halite evaporitic deposits that are also rich in other metals such as zinc, manganese, potassium, and magnesium (Tadesse *et al.*, 2003).

In other words, most extreme conditions described in previous chapters are found together in this turbulent hydrothermal system, and according to the lipid biomarkers of Dallol, it is habitable (Carrizo *et al.*, 2019b). Most of the samples collected at the Dallol dome by Carrizo *et al.* (2019b) included short chain *n*-alkanes, fatty acids, and *n*-alkanols with predominance of even-over-odd congeners *≤*C_22_. Accordingly, the LMW/HMW ratios of *n*-alkanes across samples consistently exceeded 1 (from 1.6 to 2.3). These microbial indicators were supported by a predominance of branched alkanes such as MMAs, pristane, and phytane. The lack of vegetation in the area immediately pointed toward a microbial origin, but the high temperatures and low pH values (<4) imply that Cyanobacteria or other phototrophs were not a preferential biosource.

Instead, other possibilities that may explain the presence of branched alkanes gain strength. For example, archaeol-derived phytane would infer the presence of archaea (Brocks and Summons, 2003), whereas tocopherol-derived pristane could indicate the presence of phytoplankton or plant material (Goossens *et al.*, 1984) possibly introduced via aeolian transport from woods and biomass extensions 7 km away from the hydrothermal area (Carrizo *et al.*, 2019b). This hypothesis may be substantiated by the detection of the phytosterols β-sitosterol and stigmastanol. Nonetheless, and just like in the iron sulfur streams (Tan *et al.*, 2018; Sánchez-García *et al.*, 2020b), the ever-changing conditions and the short and abrupt steep temperature and pH gradients of Dallol allow for microniches (Carrizo *et al.*, 2019b) where conditions for cyanobacterial growth might be suitable.

Unsaturated fatty acids such as 16:1ω7 accompanied by Chloroflexi lipid biomarkers such as long-chain wax esters, MUFAs such as 18:1ω9, or the *i*-C_18_ fatty acid (Jahnke *et al.*, 2001; Kaur *et al.*, 2015) in Dallol, in fact, support the potential for Cyanobacteria-*Chloroflexus* symbioses such as those identified in YNP (van der Meer *et al.*, 2003; Jahnke *et al.*, 2004; Zhang *et al*., 2004; Becraft *et al*., 2011; Ward *et al.*, 2012). Moreover, the unsaturated congeners 16:1ω7, 18:1ω9, 18:1ω10, and 18:2ω6 have been previously identified in YNP and have been associated to thermophiles such as Aquificales or Thermotogales (Jahnke *et al.*, 2001), which further aligns with some of the short-chain dicarboxylic acids identified in the Dallol polyextreme environment (Carrizo *et al.*, 2019b). The *iso* and *anteiso* pairs of C_15_ and C_17_ fatty acids suggest that thermophilic sulfate reducers such as *Thermotoga* or *Desulfobacter* could also be present in Dallol, which is in agreement with the results obtained from the TVZ in New Zealand (Campbell *et al.*, 2015; Kaur *et al.*, 2015) and from the Octopus Spring in YNP (Jahnke *et al.*, 2001).

All these revealing yet allusive indicatives are another example of the importance of complementary approaches combining stable isotopic analyses with the molecular analysis of lipid biomarkers to better infer or confirm the presence of any given taxonomy. For instance, δ^34^S signatures measured on both sulfate (from 6.5‰ to 11.7‰) and total sulfur (from 8.2‰ to 20.5‰) supported the participation of SRB in the microbial community of Dallol (Carrizo *et al.*, 2019b). Further, bulk δ^13^C values disclose a range of carbon fractionation patterns such as those associated to the Calvin cycle (*e.g.*, Cyanobacteria or α-, β-, and γ-Proteobacteria) and the reductive acetyl-coenzyme A cycle (*e.g.*, several species of δ-Proteobacteria, acetogenic Firmicutes, methanogenic Euryarchaeota, or chemolithotrophic Planctomycetes) (Carrizo *et al.*, 2019b).

All these results confirm a noticeable degree of microbial diversity (Table 3) and are molecular evidence of either extant life or well-preserved biosignatures of recently extinct biology (Carrizo *et al.*, 2019b) in one of the most extreme environments on Earth.

### 5.3. The emergence of life and preservational aspects in polyextreme environments

The microbial prosperity in polyextreme environments highlights how the limits of life on Earth remain uncertain. However, before expecting biosignatures in regions of the Solar System where multiple extreme conditions are acting together, it is important to ponder the likelihood for the emergence of life in settings of this sort. Hydrothermal activity and hence high temperatures are among the first candidate environments deemed as suitable for the origin of life as we know it, whether in the surface (Harvey, 1924) or in deep hydrothermal vents (Corliss et al., 1981; Baross and Hoffman, 1985). The high temperature niches in ancient hot spring systems catalyze reactions that were likely facilitated by the “crowding” of organic molecules in precipitating mineral surfaces. In turn, these surfaces harnessed redox gradients capable of providing chemiosmotic energy exploitable by the earliest *living* systems (Camprubí *et al.*, 2019). Hydrothermal systems collected a series of features (*e.g.*, abiotic generation of organics, availability of thermal energy, mineral diversity, protected microenvironments, and ionic concentrations driving proton gradients) (Camprubí *et al.*, 2019) that together may have constituted an appropriate environment to harbor the first macromolecules and subsequent living organisms.

Additional parameters such as anoxygenic conditions, high UV flux, and moderate acidity (pH 5–6)—as in subaerial systems such as El Tatio, but also as in the Hadean oceans (Macleod *et al.*, 1994)—might have helped constrain factors such as primitive catabolic routes, pigment evolution, and mineral precipitation, respectively. However, incorporating additional geochemical constraints such as hypersalinity, hyperaridity, or extremely acidic pH values (*e.g.*, acid Australian lakes or the Dallol hydrothermal system) could hinder a presumably complex and sensitive process of life emergence, rendering said environment suboptimal for the cascade of events that chemically transition from abiotic to biogenic.

The habitability of terrestrial polyextreme environments could be explained by a process of colonization of adapted extremophiles, as opposed to a primitive form of local life that arose under so many constraints. This is probably supported by a process of cellular evolution and *differentiation* to attain *adaptation*, therefore the earliest prokaryotic settlers might have required complexified lipid membranes and spore deployment.

According to the most ancient evidence of biological carbon fixation (Rosing, 1999), life on Earth took ∼800 million years to emerge; thus, it is reasonable to expect ∼1 billion years for terrestrial life to colonize primitive polyextreme environments. Since the Noachian period of potential habitability of Mars occurred 700–900 million years after the planet's formation, it is reasonable to expect preserved biosignatures in martian environments that used to hold elevated salinities, hydrothermal activity, and acidic conditions.

Despite their extreme conditions, the Dallol hot spring dome in Ethiopia and the acidic/hypersaline lakes of Western Australia collect a miscellaneous mineralogy that favors the preservation of organics in different ways. Halite encapsulation and phyllosilicates that form at low temperatures in aqueous conditions (smectite clays) are two shared mechanisms of preservation (Gil-Lozano *et al.*, 2020) that are possibly taking place in both polyextreme environments presented here. These are preservation strategies that are perhaps attainable in regions of Gale Crater on Mars due to its past aqueous environment, to its mineralogy, and to salt deposits such as the ones adjacent to the Australian acid lakes and within Dallol.

Besides, in Dallol, there are two additional mechanisms of mineral matrix preservation to consider: (1) mineralization within metal-rich mineral phases (*e.g.*, iron) and (2) xeropreservation or silica sinter entombment, which are mechanisms prevalent in other terrestrial analogs such as Río Tinto (Spain), the Yungay in Atacama (Chile), or YNP (United States), respectively. Ironically, despite the many challenges that polyextreme environments pose for life (or its emergence), it seems that the gathering of extreme conditions entails multiple strategies for the preservation of lipid biomarkers and thus, such locations are of high astrobiological interest.

## 6. Concluding Remarks, Pitfalls, and Projection

### 6.1. Conclusion

The results obtained from current and past missions to Mars have proven the importance of continuing the efforts to survey the neighboring planet. Billions of years ago, Mars was a geologically and volcanically active world, with a thicker atmosphere, warmer temperatures, and consequently, a global hydrosphere. Today, its surface is cold, hyperarid, and largely exposed to UV and ionizing radiation. With the exception of salt deposits, ices, or subsurface cavities, the present conditions of Mars insinuate a sterile surface, although the planet's past window of habitability encourages a search for preserved biosignatures.

On Earth, lipid molecules combine their universal character as biomarkers (as-we-know life) with a notable preservation potential and thus, given enough consideration for future missions, could provide a significant aid in the search for extraterrestrial life. To maximize the advantages of lipids, the astrobiology community should consider not only accompanying with the right complementary techniques (*e.g.*, stable carbon isotopic analysis) but also furthering our understanding of their molecular architectures, characterizing their distributions in terrestrial analogs, and ultimately identifying the molecular degradation patterns that diagenetic processes and other stress factors (ionizing radiation, high oxidant concentrations, and wind-dust erosion) can trigger on them upon alteration.

We have interrogated a series of extreme environments for distinctive microbial patterns according to the lipid studies presented here. Nevertheless, it is imperative to remember that molecular lipid analyses only yield so much specificity, so supplementing results with stable carbon isotope surveys is paramount to achieve higher taxonomic accuracy. Since patterns will be broad and encompassing, considering the mineralogical preservation machinery available in each analog is crucial to assemble a comprehensive map of what we can expect to find on Mars.

Hot spring environments present a truly contradictory situation. These are the best characterized settings on Earth when it comes to lipid biomarker studies (Robinson and Eglinton, 1990; Summons *et al*., 1996; Jahnke *et al*., 2001, 2004; Zhang *et al*., 2004; Pancost *et al*., 2005; Sánchez-García *et al*., 2019, 2020a; Williams *et al*., 2021), but they have been poorly prospected on Mars, with the Home Plate in Gusev being the only surveyed martian hydrothermal area (Cady *et al.*, 2018; Ruff *et al.*, 2020). Its silica sinter deposits, however, have not been examined in detail.

Assuming that life on Mars could have developed in a similar way to Earth, we conclude that near-vent putative lipid biomarkers on Mars, as a result of diagenesis, would most likely have degraded into linear alkanes derived from membrane-stabilizing compounds such as dicarboxylic acids or polyether compounds. Other candidate molecules include homohopanes as degradation products of BHPs, which are generic bacterial biomarkers (Farrimond *et al.*, 1998).

Within ancient geyser slopes, we can expect most MUFAs, MMAs, wax esters, and alkenes to degrade into shorter, saturated hydrocarbon chains. It is, therefore, vital to promote research on lipid molecular taphonomy by subjecting, for example, terrestrial silica sinter samples with known entombed organics to martian conditions. Results of this sort may help the scientific community understand and predict potential lipid fragmentation patterns, which could, in turn, provide insights as of how the saturated alkyl chains (C_10_-C_12_) found in Gale crater by *Curiosity* were formed (Eigenbrode *et al.*, 2018).

Conveniently, the case for preserving organics in a polyextreme arid environment with evaporitic units such as sulfates, halite, or carbonates is promising. Jezero and Gale craters represent hyperarid, once flooded evaporitic basins (Schon *et al*., 2012; Grotzinger *et al.*, 2014; Rapin *et al.*, 2019; Mangold *et al*., 2021), like the Negev or the Atacama deserts. The latter has extremely low water activity, which favors subsurface xeropreservation and protective mineral-organics interactions along with UV shielding.

Underground halite and gypsum units add matrix encapsulation potential to the equation. Together with the paucity of tectonics, deeper subsurface samples from evaporitic deposits within martian paleolakes or shallow coasts would be optimal scenarios that may preserve putative lipid biomolecules to a better extent than any other environment. This means that highly resistant lipid compounds and hyperarid evaporitic paleolakes/basins combine to increase the chances of uncovering more intact hydrocarbon skeletons with perhaps still intact ether backbones or isoprenoidal chains. Further, accessing UV-, perchlorate-, and chlorate-devoid samples (martian iron [hydr]oxide-rich conditions also favor the accumulation of chlorates) (Qu *et al.*, 2022) using enhanced drills such as the 2 m device loaded onto the *Rosalind Franklin* rover (Veneranda *et al.*, 2021) could be deemed as an attainable objective.

Finally, acidic streams open a window to regions such as *Meridiani Planum* during the Noachian and the early Hesperian geological periods of Mars, where liquid water seemed to be consistently acidified by the ferric sulfate minerals identified by *Opportunity* (Klingelhöfer *et al.*, 2004; Squyres *et al.*, 2004; Zolotov and Shock, 2005). Studies on analogs such as Río Tinto demonstrate an increasing microbial diversity and lipid complexity with depth (Sánchez-García *et al.*, 2020b), probably away from the relatively higher degradation potential of hydrological activity of the surface and from excessively low pH niches.

If similar processes were involved in *Meridiani Planum*, deeply buried organics could be protected from the oxidizing surface and from UV radiation. The state of these lipids will depend on the encapsulation potential of poorly arranged goethite and ferrihydrite minerals found on the plains of Eagle crater, but studies at the Chocolate Pots validate that intact, functionalized fatty acids can be safeguarded within the ferric mineral matrix (Parenteau *et al.*, 2014).

It is exactly these combinations of mineral matrices, geochemical processes, and biological pathways that prompt the possibility of extraterrestrial life, but also what empowers the interdisciplinary nature of astrobiology. The field remains at its most exciting stage; not having found any form of life yet, but with the technology and an escalating knowledge to do so. We propose a combined molecular and isotopic fingerprinting of lipids as a powerful tool to contribute toward the deciphering of the question that keeps the scientific community expectant: was there or is there any life on Mars?

### 6.2. Limitations

Unveiling the molecular and isotopic fingerprints of life in extreme environments on Earth showing a close resemblance to present or past scenarios on Mars provides criteria to recognize signs of prebiotic chemistry or hypothetical life (as we know it) on our neighboring planet. However, like with any other biomarker approach, the use of lipids entails limitations and uncertainties that must be considered in the search for extraterrestrial life.

A common issue in lipid extraction procedures and GC-MS methodologies is contamination, where field or laboratory apparatus can easily introduce lipid biomarkers to a fresh sample, or where organic compounds manage to leach toward the interior of rocks. It is, therefore, essential to compare sample surfaces versus interiors, as well as working in a strictly sterile environment, thoroughly checking anomalous signals and testing samples multiple times in more than one facility (Summons *et al.*, 2022).

What truly demands caution is the yet unknown process of interpreting signs of life beyond Earth. Given the abstruseness of the concept of *biogenicity*, there are potential “false positives” involving morphologies, textures, patterns, or compounds capable of forming abiotically. Nonetheless, there are, indeed, strategies that permit the distinction of abiotically synthesized from biologically generated lipid molecules.

The molecular abundances and atomic distributions of different lipid fractions (alkanes, carboxylic acids, or alkanols) can be interpreted in several ways that may help pinpoint their origin. For example, abiotic Fischer-Tropsch generation of hydrocarbon chains almost always results in typical Schultz-Flory probability distributions, that is, a systematic decrease of molecule abundance with an increase in the number of carbon atoms in the chain (McCollom *et al.*, 1999).

As the chain grows, the probability of said growth can be expressed as a ratio (α) between molecules with successive carbon numbers (*C_n_*_+__1_/C_*n*_) (Szatmari, 1989), and so, the ratio for abiotically generated lipids remains almost constant throughout the elongation of the chain (α ≈ 0.6). In contrast, biological alkanes generate erratic and dissimilar α values. Moreover, while abiotically generated hydrocarbon chains display a clear lack of even-over-odd or odd-over-even carbon number dominance (McCollom *et al.*, 1999), biologically synthesized lipids have a preference toward even or odd carbons (Georgiou and Deamer, 2014).

Also, in a typical GC-MS chromatogram, peaks from biotic compounds usually stand out from the rest (*e.g.*, cyanobacterial heptadecane and methylated alkanes, *Chloroflexus*-associated long-chain polyunsaturated alkanes, isoprenoids or hopanes) (Georgiou and Deamer, 2014).

This review has claimed how the use of stable carbon isotope chemistry can aid in biosource identification of certain lipid biomarkers, but it is also a suitable approach to discern biological from non-biological. Highly depleted δ^13^C values (*i.e.*, strong fractionations) in lipids are typically related to biological sources, and enzymatic carbon discrimination is the only known natural process to produce large fractionations on long-chain carbonaceous molecules (Peters *et al.*, 2005), at least on Earth.

In the laboratory, however, Fischer-Tropsch synthesis reactions have yielded hydrocarbon chains depleted by 36‰ relative to atmospheric carbon (δ^13^C approximately −8‰) (McCollom and Seewald, 2006; McCollom *et al.*, 2010), which is relevant to naturally occurring Fischer-Tropsch reactions on interstellar nebulae and parent bodies (Kress and Tielens, 2001). However, these ^13^C-depleted and abiotically generated lipid compounds can be distinguished from biological ones thanks to their archetypal constant ^13^C composition across all intramolecular carbons (McCollom and Seewald, 2006). On the other hand, biosynthesized compounds display alternating δ^13^C values between even- and odd-numbered carbons within the alkyl chain due to their assembly mechanisms.

Thus, supplementing a clean and meticulous molecular analysis with an isotopic probing of intramolecular carbon atoms in multiple lipid biomarkers should minimize the risk of false positives when assessing prospective biological signatures. Biosynthesis will be, in turn, supported by a higher frequency of anomalies with increasing complexity in the molecular profile (Marshall *et al.*, 2017; McMahon and Cosmidis, 2022). All these aspects must be considered when searching for signs of a hypothetical extraterrestrial life, at least as we know it, with the biomolecules that have been amply scrutinized on Earth.

On that note, the universality of lipids as biomarkers from the ever-present cell membrane along with their recalcitrant character grants lipids a significant, double astrobiological value as tools to be employed in the search for life beyond Earth.

## Supplementary Material

Supplemental data

## Abbreviations Used

ACL: average chain length
AI: Aridity Index
BHP: bacteriohopanepolyol
BP: Bison Pool
CAB: Centro de Astrobiología
CPI: carbon preference index
*cy*-FA: cyclopropane fatty acid
DGD: dialkyl glycerol diether
DMA: dimethyl alkane
GC: gas chromatography
GDGT: glycerol dialkyl glycerol tetraether
HMW: high molecular weight
*i*/*a*: *iso*/*anteiso*
LMW: low molecular weight
masl: meters above sea level
MER: Mars Exploration Rovers
MMA: monomethyl alkane
MOMA: Mars Organic Molecule Analyser
MS: mass spectrometry
MUFA: monounsaturated fatty acid
OK: Orakei Korako
OS: Octopus Spring
Pr/Ph: pristane over phytane
PUFA: polyunsaturated fatty acid
rRNA: ribosomal RNA
SAM: Sample Analysis at Mars
SRB: sulfate-reducing bacteria
TVZ: Taupo Volcanic Zone
UV: ultraviolet
YNP: Yellowstone National Park
