# Supplementary material for: An Overview of Lipid Biomarkers in Terrestrial Extreme Environments with Relevance for Mars Exploration
Source: Astrobiology. 2023 Apr 28;23(5):563–604. doi: 10.1089/ast.2022.0083 (PMC10150655; doi:10.1089/ast.2022.0083)
Supplement: Supplemental data [file Suppl_appendix.docx]

**Appendix**

*1. n-alkanes, methylated alkanes and alkenes*


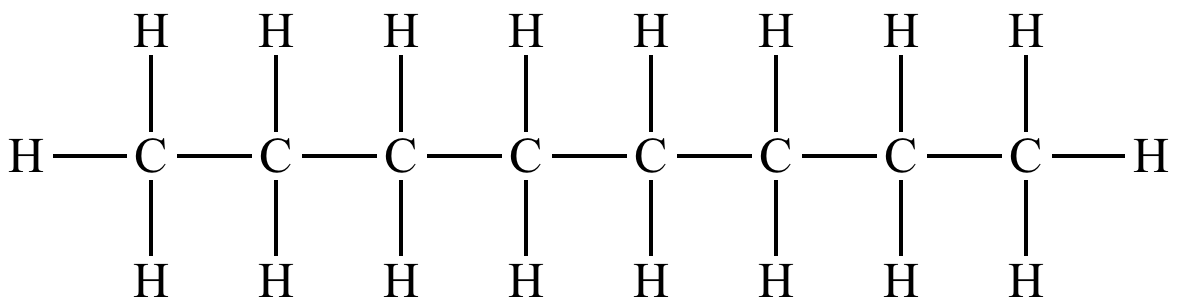


A


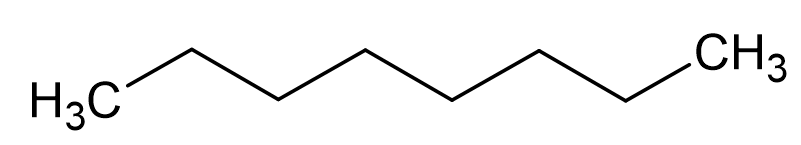


B

C

**A)**
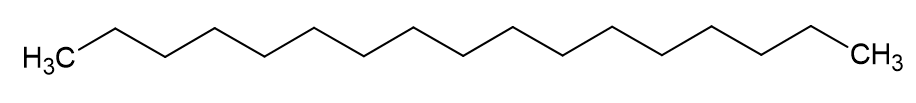
Structure and chemical depiction of **B)** **octane** and **C)** **heptadecane**, both examples of C_8_ and C_17_ *n*-alkanes.


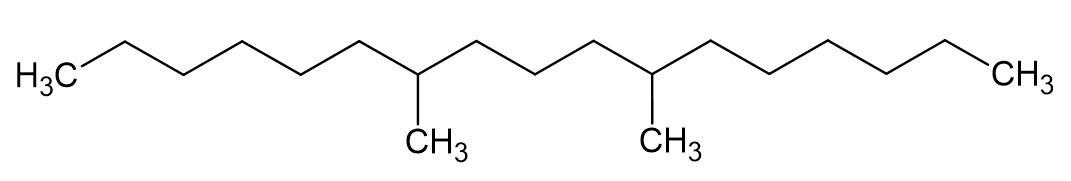


B

A

**A)** **
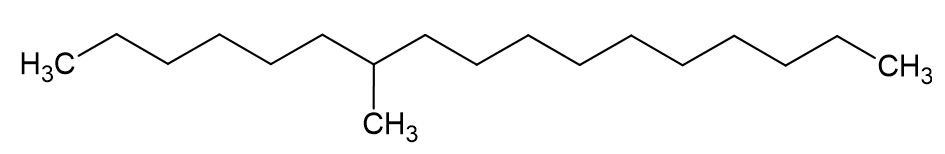
7-Methylheptadecane (Me-C_17_)** and **B)** **7,11-Dimethylheptadecane (DiMe-C_17_)** are examples of branched, methylated alkanes.

**A)**
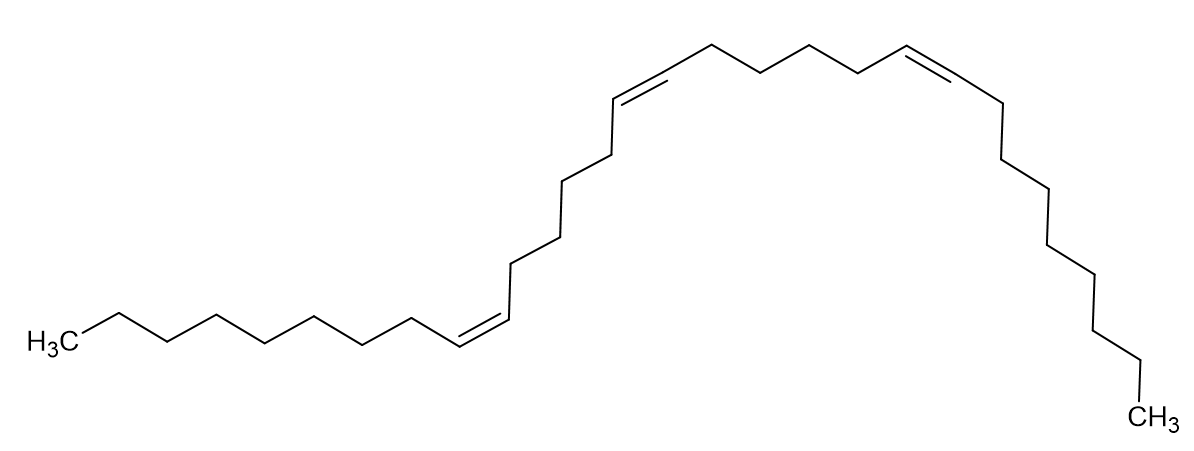

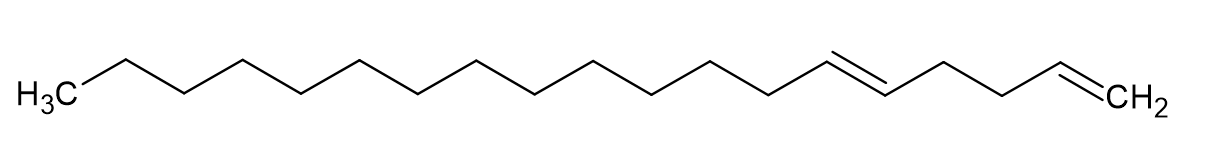

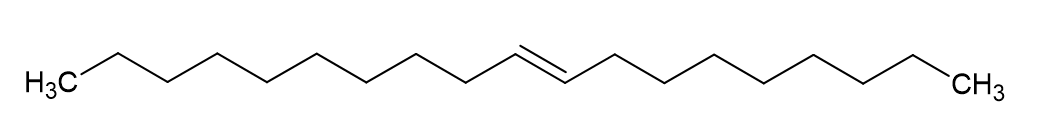
**9-Nonadecene** (**C_19:1_**), **B)** **1,5-Nonadecadiene** (**C_19:2_**) and **C)** **Hentriacontatriene** (**C_31:3_**) are examples of alkenes with one or multiple unsaturations (double bonds).

B

C

A

*2. Alkanoic acids*


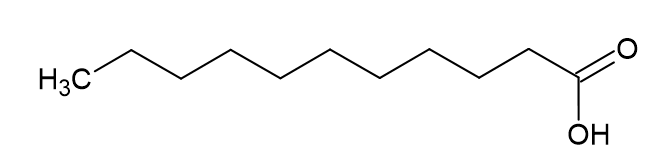


A


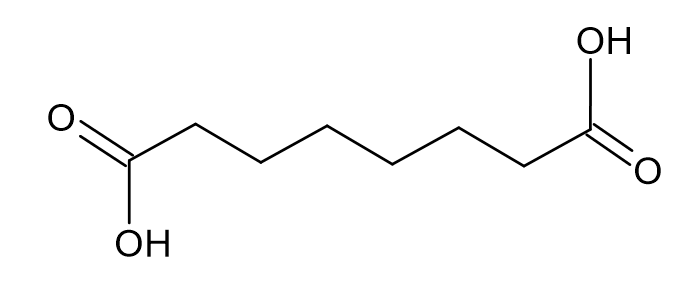


B

**A) Undecanoic acid** (**11:0**), also known as **undecylic acid**, is an example of an alkanoic acid that is long enough to be considered a fatty acid.

**B)** **Octanedioic acid**, also known as **suberic acid**, is an example of a dicarboxylic acid, containing two terminal carboxylic groups (-COOH).


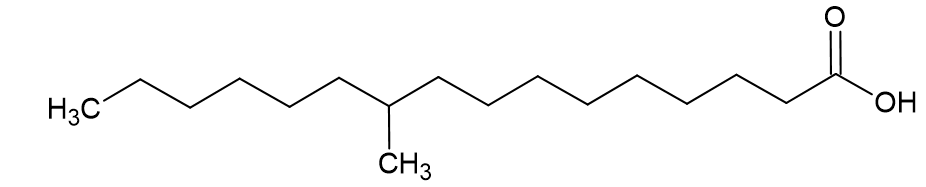


B

A


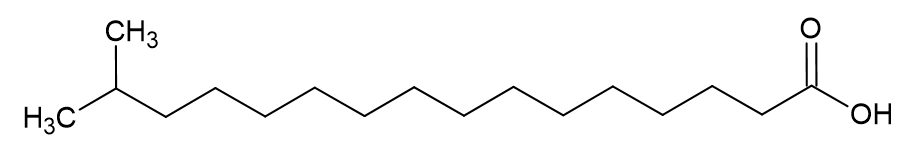


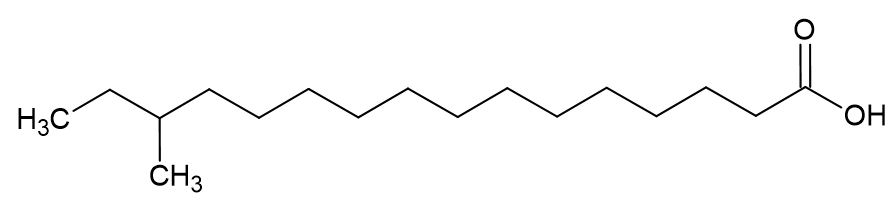


C

These are examples of methyl-branched fatty acids. **A)** is methylated in the 10^th^ carbon starting from the carbon bearing the carboxyl group (Δ notation), and thus, this compound is addressed as **10-Methylhexadecanoic acid** **(10-Me16:0)**.


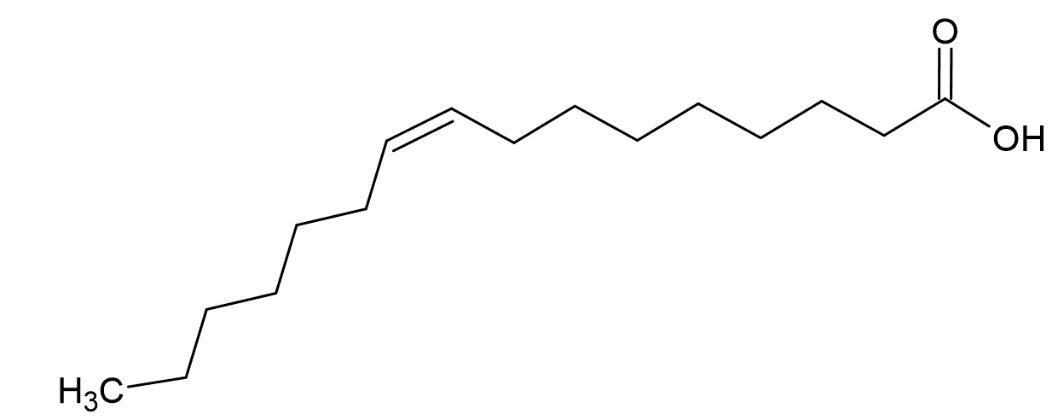
The following two alkanoic acids are **B)** *iso* and **C)** *anteiso* fatty acids, meaning they have the methyl group (-CH_3_) in the second-to-last (*iso*) and third-to-last (*anteiso*) carbons in the alkyl chain. **B)** is called **15-Methylhexadecanoic acid (*iso*-C_17_)** and **C)** is **14-Methylhexadecanoic acid (*anteiso*-C_17_)**.

A


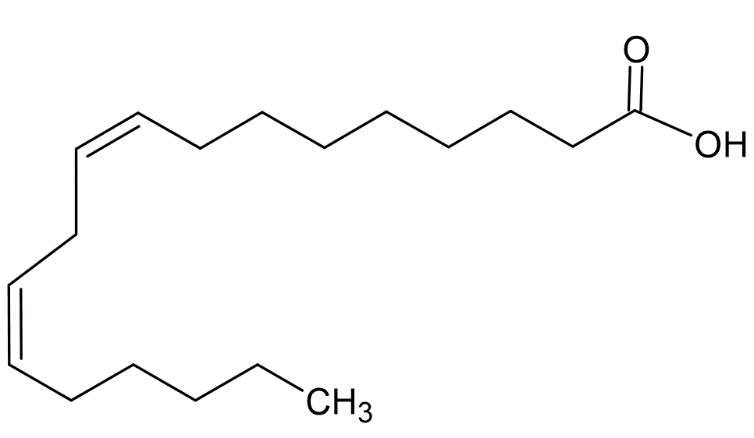


B

**A) 7-Hexadecenoic acid** (**16:1ω7**), also known as **palmitic acid**, has an unsaturation in the 7^th^ carbon starting from the methyl end (16^th^ carbon) according to the ω notation, which is commonly used with unsaturated acids. Unsaturations introduce “bends” in alkyl chains.

**B)** **6,9-Octadecenoic acid** (**18:2ω6,9**), also known as **linoleic acid**. Often, only the first unsaturation is noted: **18:2ω6**.


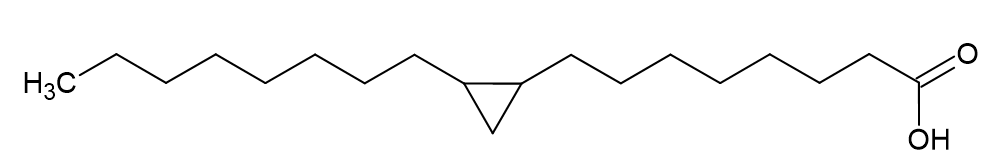

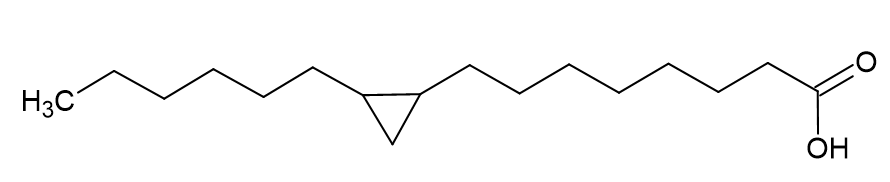


B

A

Cyclopropyl fatty acids contain a cyclopropane group within the alkyl chain.

The IUPAC nomenclature of the **A)** is **2-Hexylcyclopropaneoctanoic acid**, but since the total number of carbon atoms is 17, we abbreviate to **cyclopropyl-C_17_ (*Cy-*C_17_)**. Such is the case for **B)** **2-Octylcyclopropaneoctanoic acid (*Cy-*C_19_)**.

*3. Alkanols and alcohol membrane constituents*


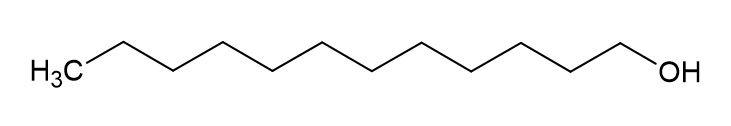


A


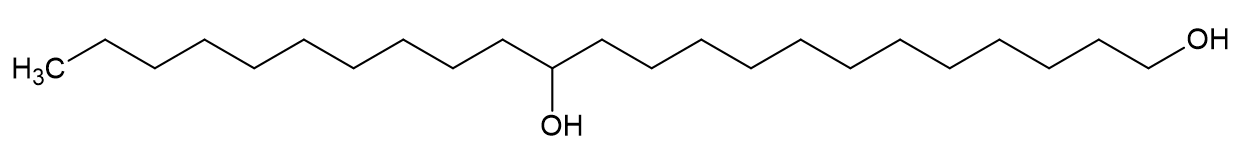


B

**A) 1-Dodecanol** (or *n*-dodecanol) is an alkanol, which is a typical alkyl chain with a hydroxyl group (-OH) in the first carbon. **B) 1, 13-Tricosanediol** is a diol, which contains two hydroxyl groups.


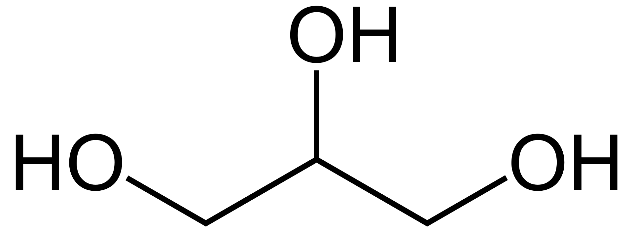


**1,2,3-Propanetriol**, also known as **glycerol**, is the basic triol required for the formation of the amphipathic lipids that form the lipid bilayer.


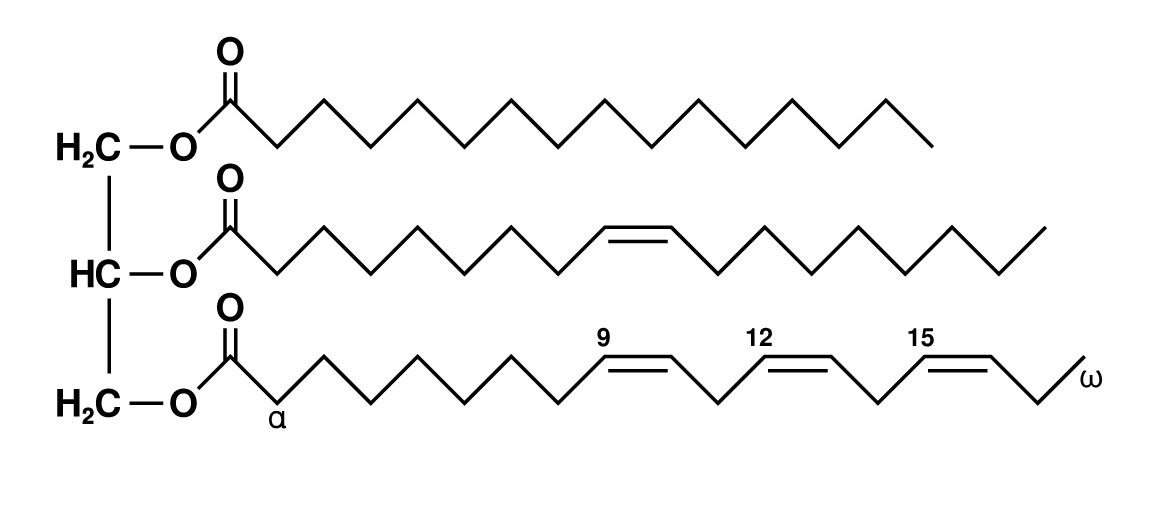


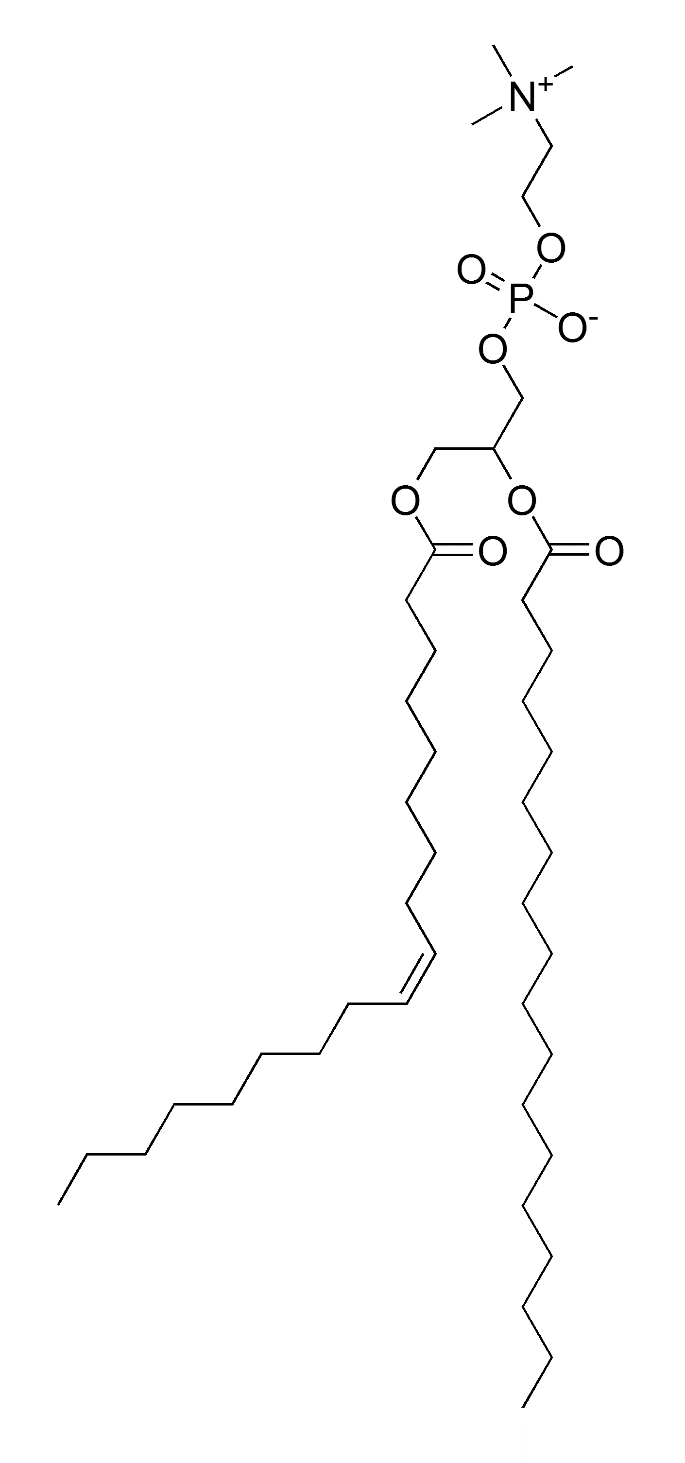


Above is the structure of a **triglyceride**. It consists of a **glycerol** molecule (left, dark green) bound by three ester bonds (light green) to three fatty acids (three alkyl chains on the right side). Some of these bound fatty acids may or may not be unsaturated.

The above compound is a **phospholipid**. In this occasion, the **glycerol** that was ester bound to the three fatty acids is bound to only two of them (in green). The remaining hydroxyl (-OH) group is now bound to a **phosphate** group (in blue) by a phosphoester linkage. This forms the basic structure of a phospholipid.

These molecules can bind further polar groups such as **choline** (in red), which provides an even greater polarity that is counteracted by the apolar alkyl chains. Thus, the full name of the above molecule is **phosphatidylcholine**. This is the basic monomer that upon interaction with other congeners of its kind forms the lipid bilayer, the basis of the plasma membrane.

*4. Isoprenoids*


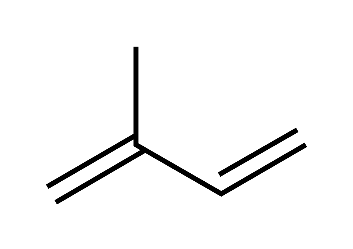

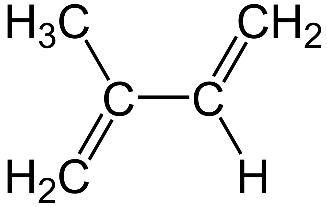


Structure (left) and chemical depiction (right) of **isoprene (C_5_H_8_)**, the basic unit that upon elongation forms highly branched isoprenoidal chains.


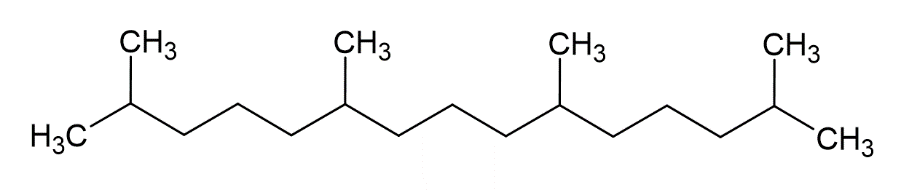


A


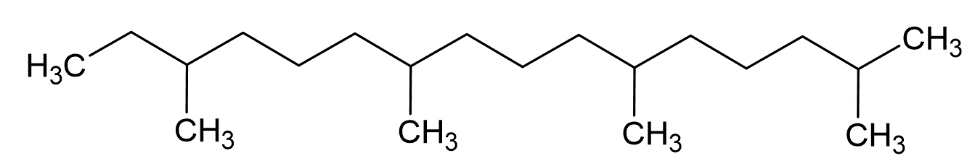


B

**A) 2,6,10,14-Tetramethylpentadecane**, commonly addressed as **pristane**, is a saturated isoprenoid formed by isoprene elongation. It can be derived from the phytol side-chain of chlorophyll.

**B)** **2,6,10,14-Tetramethylhexadecane**, or **phytane**, can be derived from phytol but also from other compounds found in the membranes of Archaea.


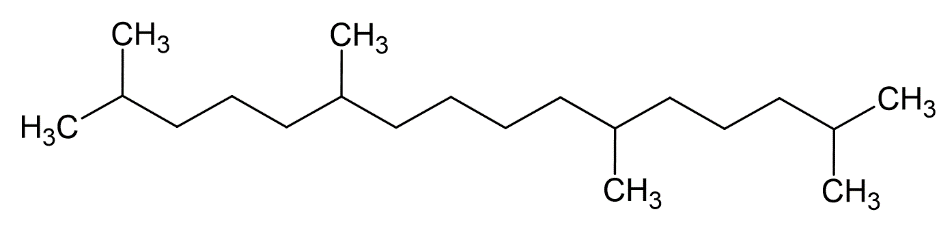


A


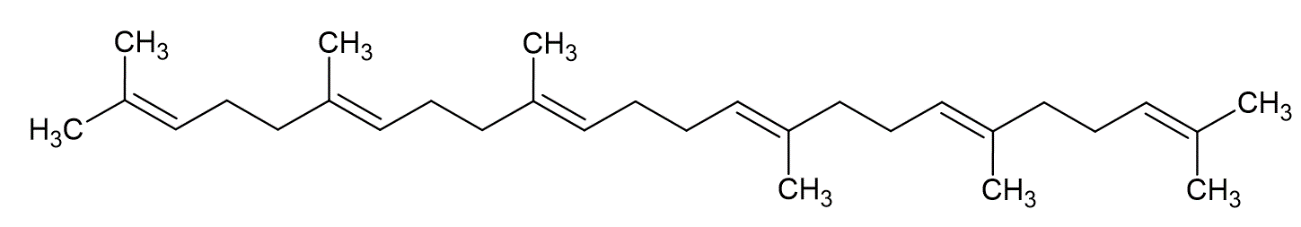


B

**A) 2,6,11,15-Tetramethylhexadecane**, also known as **crocetane**, is an isomer of phytane and a biomarker associated with Archaea or with anaerobic methane oxidation.

**B) Squalene** is also an isoprenoid composed of six isoprene units, also known as a linear triterpenoid and a precursor to all steroids.


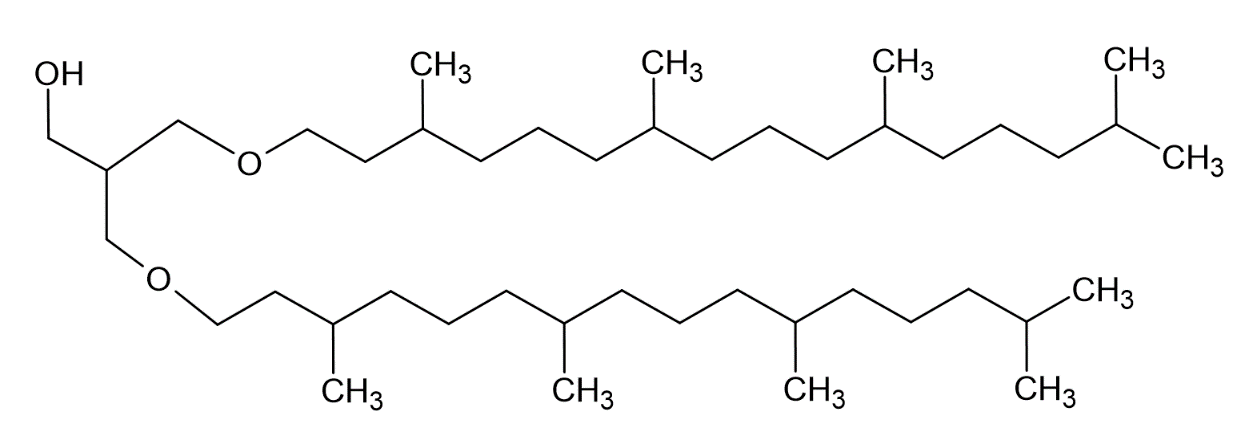

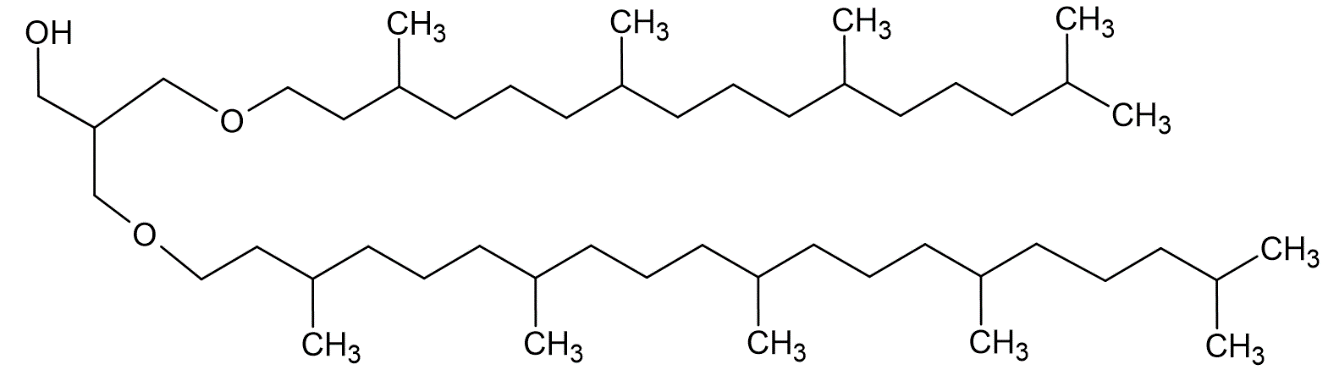

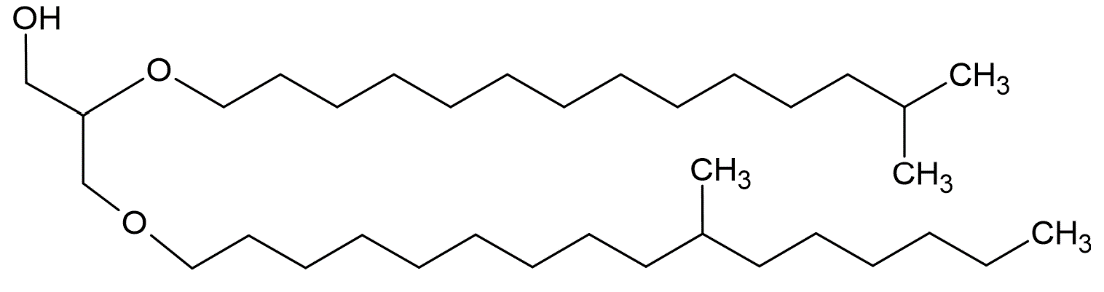


A

B

C

**A) Archaeol** and **B) extended archaeol** are examples of dialkyl glycerol diethers (DGDs). *Dialkyl* because of the two isoprenoid alkyl chains, *glycerol* because they are bound to a glycerol molecule (in green) and *diether* because the isoprenoid chains are bound to the glycerol by ether bonds (O atoms within the green circles) instead of ester bonds. These molecules form the basic structure of the archaeal lipid bilayer, which is more resistant thanks to the ether linkages.

**C)** is a **bacterial DGD**, which still has ether bonds but no isoprenoidal chains. Instead, the C_14_-C_16_ alkyl chains are branched with methyl (-CH_3_) groups.


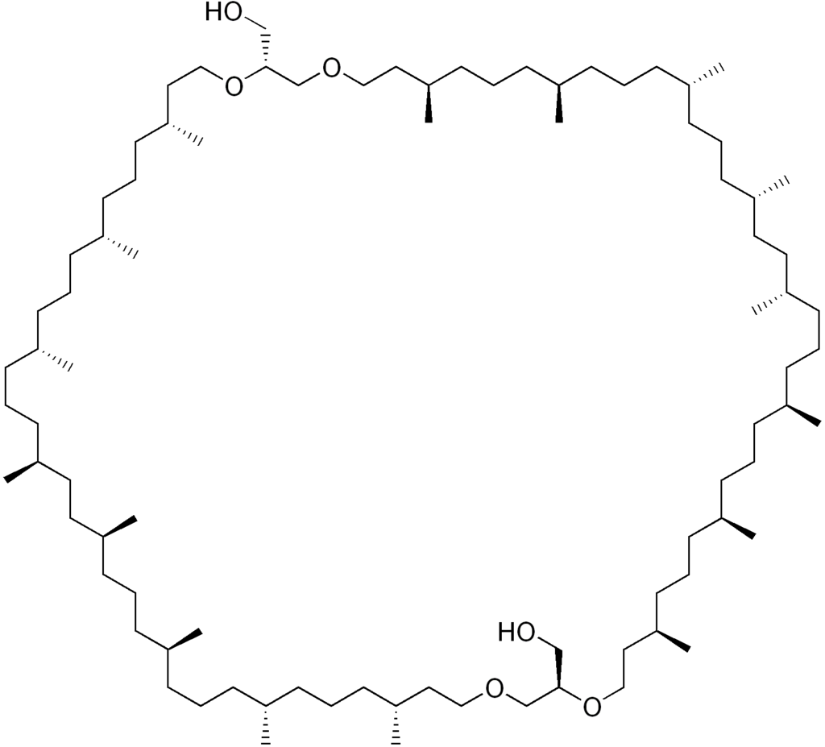


A


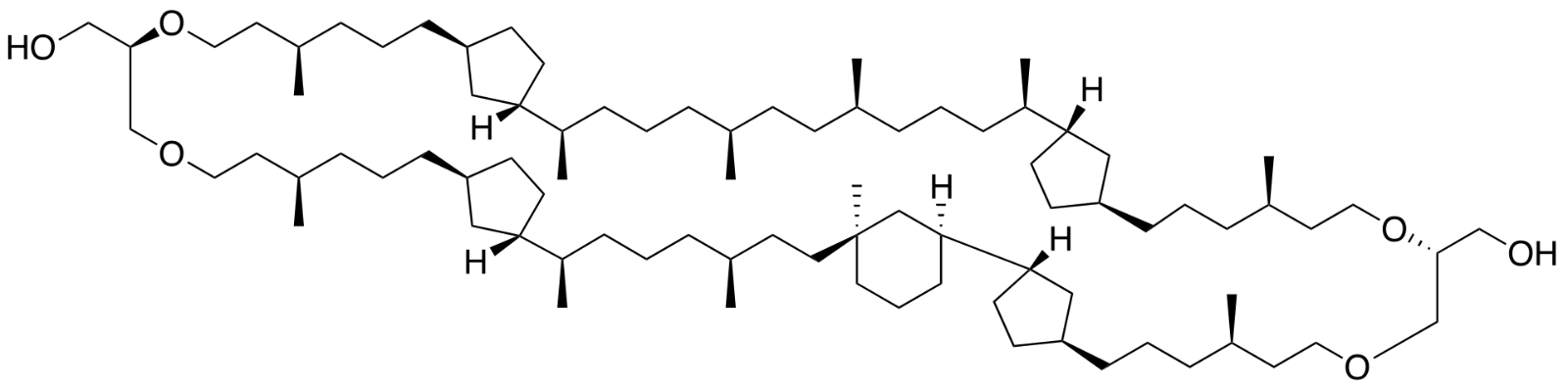


B


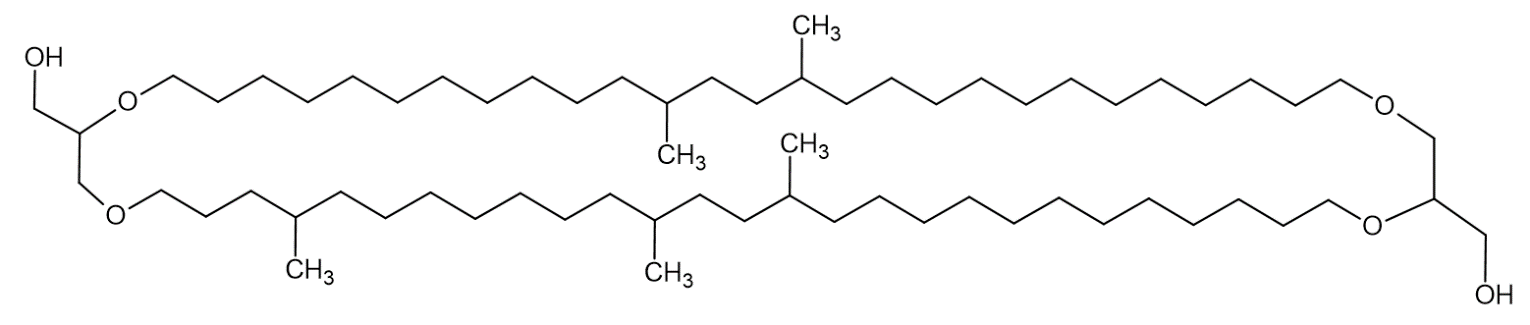


C

**A) Caldarchaeol**, an archaeal glycerol dialkyl glycerol tetraether (GDGT). These large membrane components come together to form lipid monolayers in Archaea, as opposed to typical lipid bilayers found in the domains of Bacteria (via phospholipids) and Archaea (via diether compounds). Unlike DGDs, GDGTs contain two glycerol groups (hence the repetition of *glycerol* in its nomenclature) and four ether bonds (hence *tetraether*). Caldarchaeol is an example of an isoprenoidal GDGT. **B)** **Crenarchaeol** is a more complex isoprenoidal GDGT present in some species of Archaea. It provides more membrane rigidity thanks to its four cyclopentane rings and to an additional cyclohexane.

As DGDs, **C)** is a **bacterial GDGT** with two C_29_-C_30_ branched, alkyl chains that are not of isoprenoidal nature. Some bacteria can display GDGT monolayers.

*5. Carotenoids*


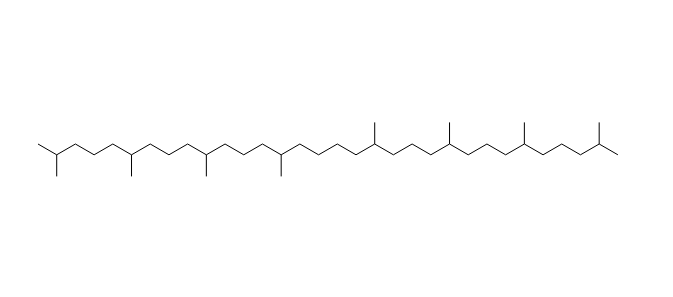

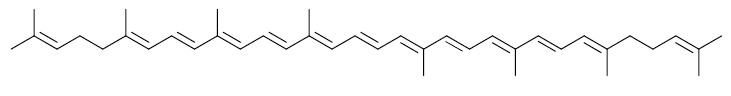


A


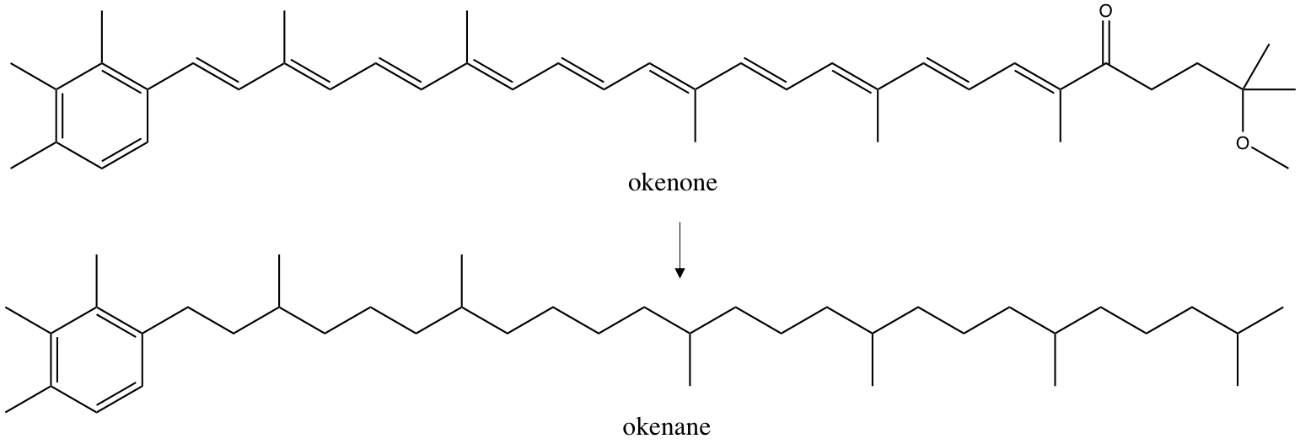


B

The above structures are examples of **carotenoids**, which are organic, light-harvesting pigments of diverse coloration that are common in the membranes of phototrophic microorganisms. Carotenoids are **tetraterpenoids**, meaning they are also isoprenoid compounds formed by the elongation of eight isoprene units. Above there are two types of fresh pigments (top) that upon diagenesis undergo degradation into their saturated products (bottom).

**A) Lycopene** is a red light-reflecting carotenoid that upon saturation (loss of double bonds) evolves into **lycopane** (saturated – no double bonds).

**B) Okenone** is a carotenoid with an aromatic ring (left side of the molecule) and a ketone group (highlighted in red). Upon degradation, okenone loses unsaturations and ketone groups, where **okenane** (saturated alkyl chain) becomes a common degradation product.


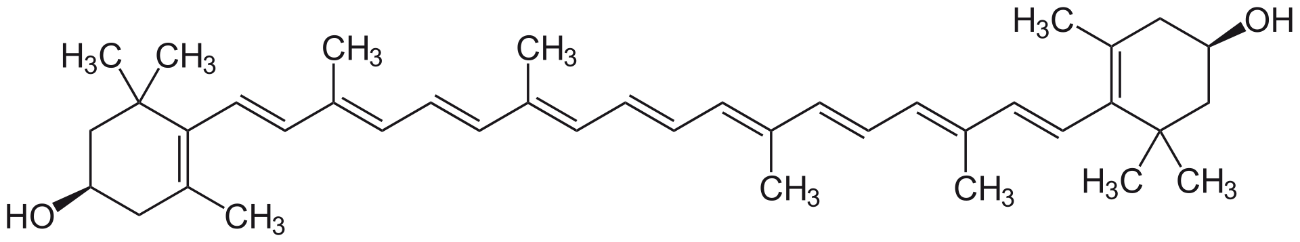


A


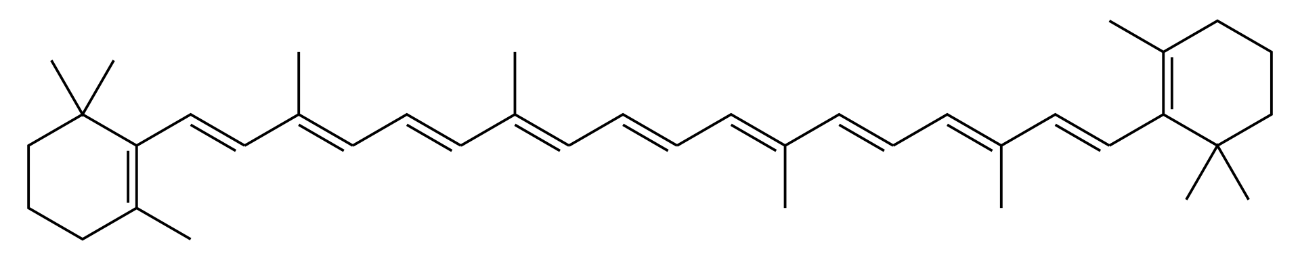


B


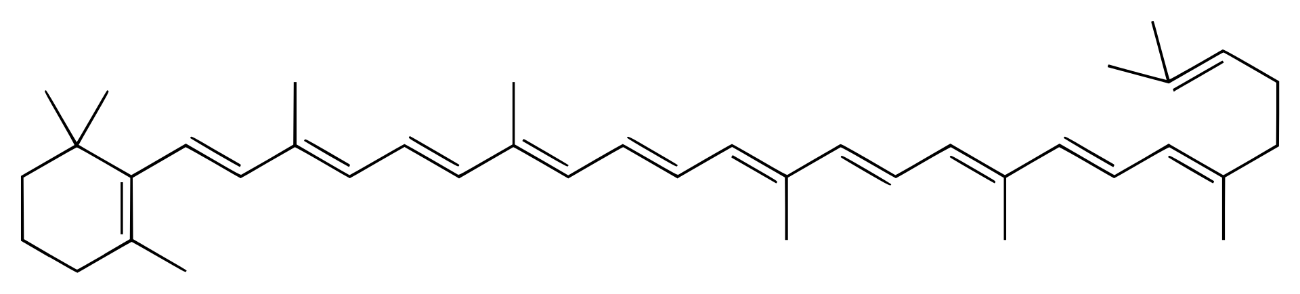


C


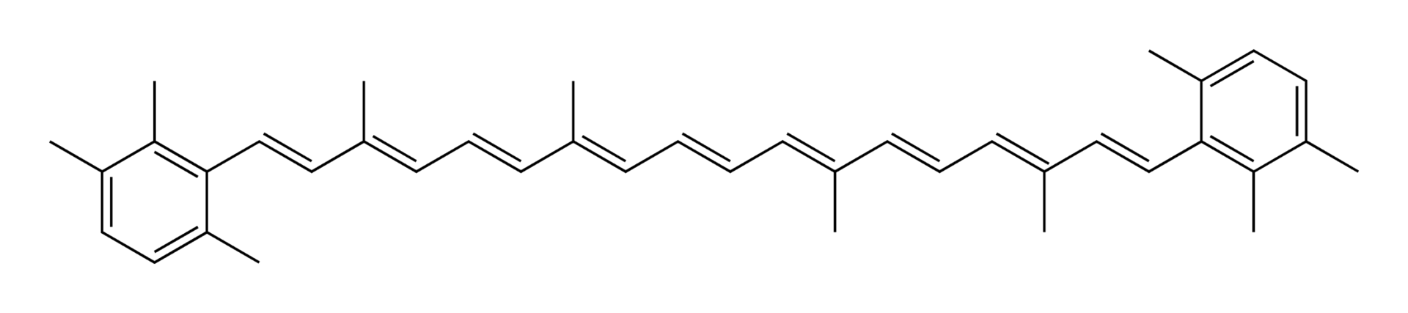


D

Other examples of **carotenoids** and their diverse fragmentation patterns:

**A)** is **zeaxanthin**, where methyl (**-CH_3_**) and hydroxyl (**-OH**) groups are depicted for clarification purposes. Zeaxanthin reflects yellow light and provides a yellow coloration to microorganisms that bear it within their plasma membranes.

**B)** is **β-carotene**, a common pigment that reflects orange light in microbes and especially in higher plants. A similar molecule is **C) γ-carotene**, which absorbs a similar wavelength.

**D)** depicts the more unsaturated **isorenieratene**, produced by green sulfur bacteria.

When carotenoids fragment, it is common to obtain molecular products like aromatic rings (shown in blue), linear isoprenoid chains of varying lengths (in green) or trimethyl aryl compounds (in red) that are composed of a trimethylated aromatic ring and part of an isoprenoid chain.

*6. Hopanoids*


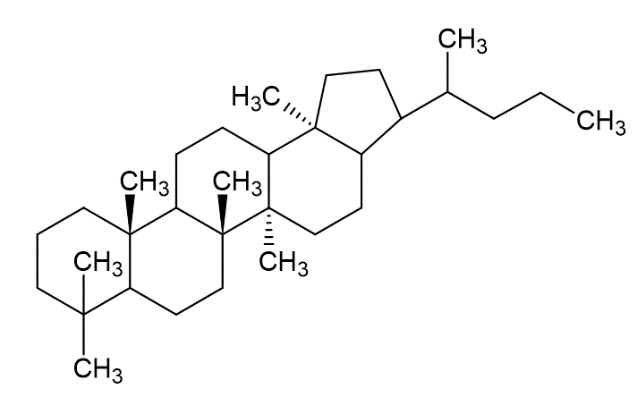

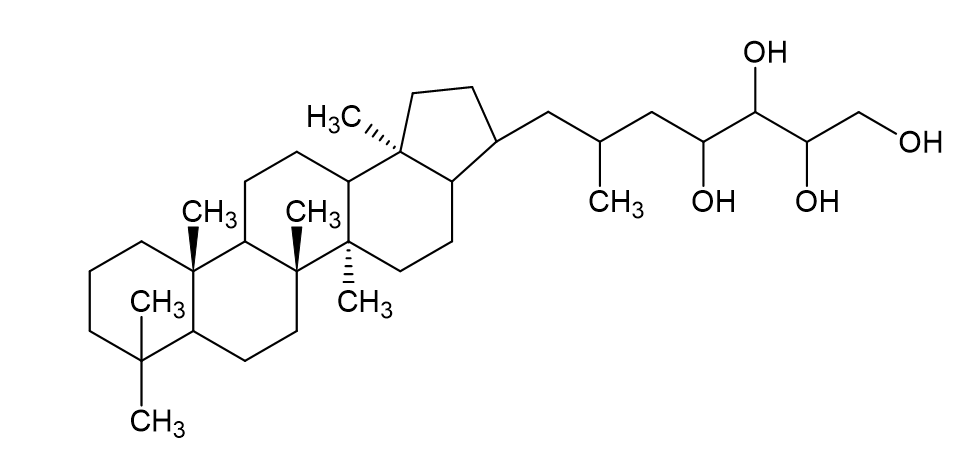

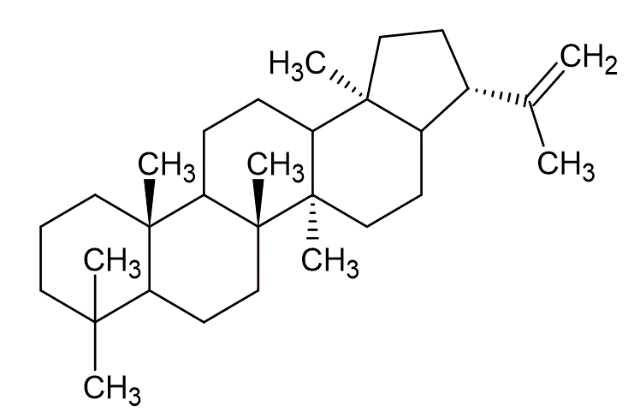

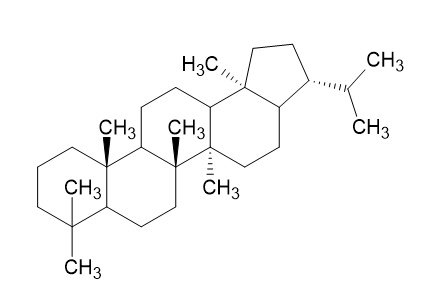


D

A

C

B

Hopanoids are a family of pentacyclic triterpenoids whose occurrence is prominent in bacteria, plants and fungi, and they all share the hydrocarbon skeleton of **A) Hopane**.

**B) Diploptene** is a common hopanoid that only differs from hopane in an unsaturation at carbon 22 (in red). The position of carbon 22, which can be “inwards” (called S epimer) or “outwards” (R epimer) is important when defining the stereochemistry and maturity of hopanes.

**C)** is a **bacteriohopanepolyol (BHP)**, very ubiquitous within bacterial membranes. This congener, due to its four hydroxyl groups (in blue), is called **bacteriohopanetetrol**.

Upon maturity, BHPs lose hydroxyl groups but remain extended (keeping the alkyl chain stemming from carbon 21, highlighted in green). These more mature molecule are addressed as **D) homohopanes**, and the alkyl chain extension is variable. For example, the degraded homohopane in D) has an alkyl chain extension with two more carbons than hopane (in purple), and thus, it is addressed as **bishomohopane**.


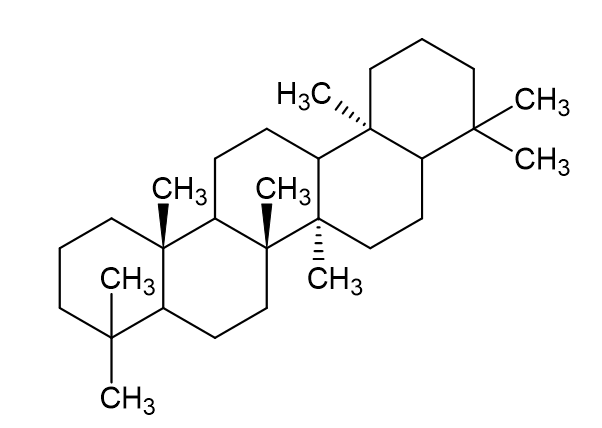

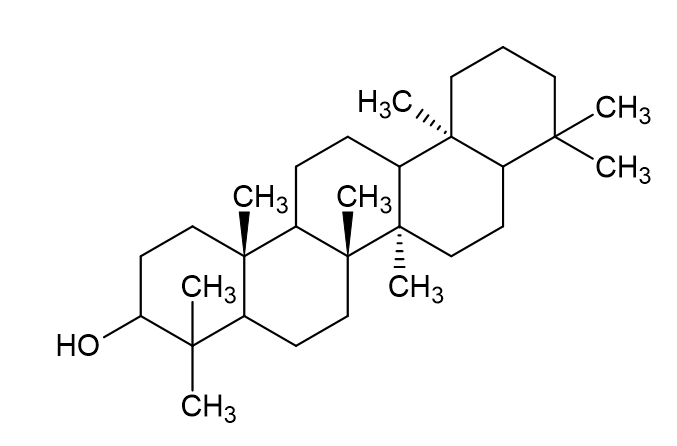


B

Ac

**A) Tetrahymanol** is the precursor molecule of **B) gammacerane**, and they only differ by a hydroxyl group (in red). Tetrahymanol and gammacerane are also pentacyclic triterpenoids with quasi-hopanoid skeletons, where the fifth ring is a cyclohexane rather than a cyclopentane.
